# Supplementary material for: High-efficiency gold recovery by additive-induced supramolecular polymerization of β-cyclodextrin
Source: Nat Commun. 2023 Mar 9;14:1284. doi: 10.1038/s41467-023-36591-0 (PMC9998620; doi:10.1038/s41467-023-36591-0)
Supplement: Supplementary file 1 — Supporting Information [file 41467_2023_36591_MOESM1_ESM.pdf]

Supplementary Information for

High-efficiency gold recovery by additive-induced supramolecular polymerization of β-cyclodextrin

Huang Wu[1,6], Yu Wang[1,6], Chun Tang[1], Leighton O. Jones[1], Bo Song[1], Xiao-Yang Chen[1], Long Zhang[1], Yong Wu[1], Charlotte L. Stern[1], George C. Schatz[1], Wenqi Liu[2✉], and J. Fraser Stoddart[1,3,4,5✉]

[1]*Department of Chemistry, Northwestern University, 2145 Sheridan Road, Evanston, Illinois 60208, United States*

[2]*Department of Chemistry, University of South Florida, 4202 East Fowler Avenue, Tampa, Florida, 33620, United States*

[3]*School of Chemistry, University of New South Wales, Sydney, NSW 2052, Australia*

[4]*Stoddart Institute of Molecular Science, Department of Chemistry, Zhejiang University, Hangzhou 310027, China*

[5]*ZJU-Hangzhou Global Scientific and Technological Innovation Center, Hangzhou 311215, China*

[6]*These authors contributed equally: Huang Wu and Yu Wang*

To whom correspondence should be addressed:

✉E-mail: wenqi@usf.edu ✉E-mail: stoddart@northwestern.edu

Supplementary Note 1. Crystallographic characterization

All crystallographic data are available free of charge from the Cambridge Crystallographic Data Centre (CCDC) via www.ccdc.cam.ac.uk/data_request/cif.

(1) Crystal superstructure of $KAuBr_4 \subset \beta$-CD

(a) Method. Brown block-like crystals were obtained by slow cooling of an aqueous solution of $KAuBr_4$ (100 mM) and β-CD (100 mM) from 90 °C down to room temperature over 6 h. A suitable crystal was selected and mounted on a MITIGEN holder with Paratone oil on an XtaLAB Synergy, Single source at home/near, HyPix diffractometer. The crystals were kept at 100.07 K during the data collection. Using Olex2,[1] the structure was solved with the ShelXT[2] structure solution program using Intrinsic Phasing and refined with the XL[3] refinement package employing Least Squares Minimization. Crystallographic images were produced using Mercury 4.3.0. Distances were measured employing Mercury 4.3.0. The solid-state superstructure of $KAuBr_4 \subset \beta$-CD is shown in Fig. 1f and Supplementary Fig. 1.

(b) Crystal Parameters. $C_{42}H_{70}O_{35}$•$KAuBr_4$•4(H_2O). Mr = 1762.75. Brown sheet (0.256 × 0.197 × 0.058 mm^3). Monoclinic, space group $P2_1$ (no. 4), a = 14.90540(10), b = 15.43740(10), c = 14.91910(10) Å, α = 90.000, β = 119.3100(10), γ = 90.000°, V = 2993.43(4) Å^3, Z = 2, T = 100.07(16) K, μ(CuKα) = 9.299 mm^{-1}, $Dcalc$ = 1.956 g/mm^3, 67088 reflections measured (6.794 ≤ 2Θ ≤ 159.682), 12191 unique (R_{int} = 0.0375, R_{sigma} = 0.0273) which were used in all calculations. The final R_1 was 0.0270 ($I > 2\sigma(I)$) and wR_2 was 0.0711 (all data).

(c) Refinement and solvent treatment details. No special refinement was necessary in the case of solving the solid-state superstructure of $KAuBr_4 \subset \beta$-CD.

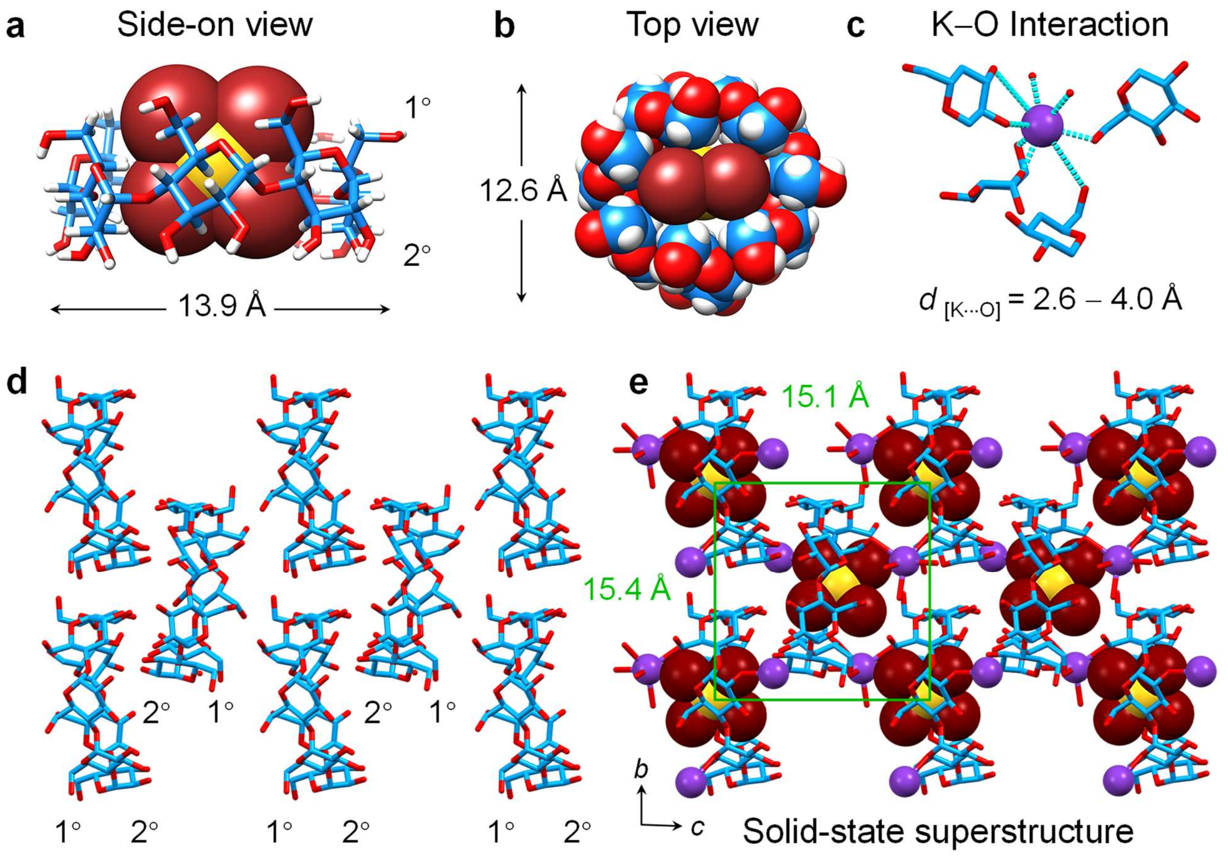

Supplementary Figure 1 | X-Ray single-crystal superstructure of the $KAuBr_4 \subset \beta$-CD complex. (a, b) Capped-stick and space-filling representations of the solid-state superstructures of $KAuBr_4 \subset \beta$-CD. (c) Ball-and capped-stick representation of the K–O interactions, showing that each K^+ ion forms eight sets of $[K^+ \cdots O]$ coordinative bonds with four glucose residues and two water molecules. The K^+ ions are located on both the primary and secondary faces of β-CD tori and interact with four β-CD tori. (d) Capped-stick representation of the packing of the β-CD tori, showing that the two primary faces and two secondary faces of adjacent β-CD tori adopt a displaced staggered stack. (e) Capped-stick and space-filling representation of the crystal packing of β-CD and $KAuBr_4$, showing the relative positions of K^+ cations, $[AuBr_4]^-$ anions, and the β-CD tori. The "1 º " represents the primary face and the "2º " represents the secondary face of β-CD. Solvent molecules have been omitted for the sake of clarity. H white, C skyblue, O red, Br brown, Au yellow.

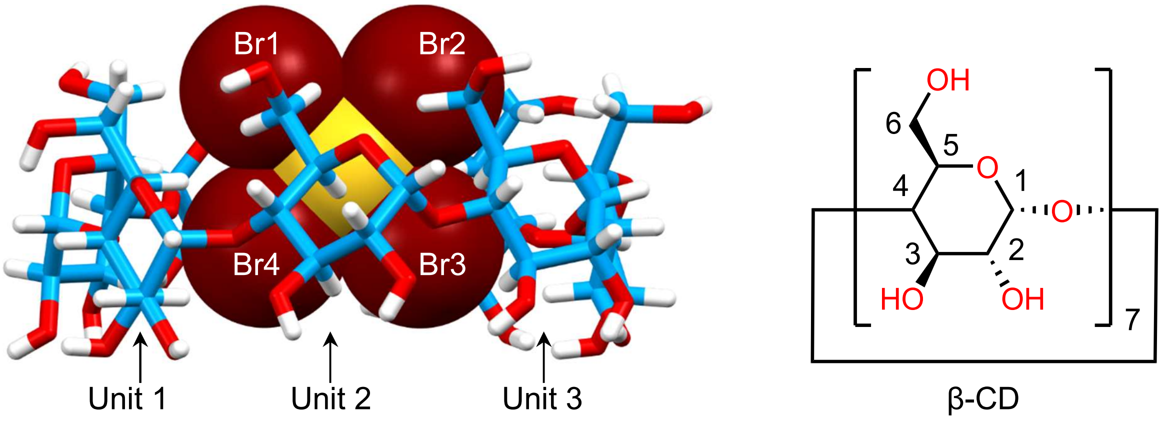

Supplementary Figure 2 | Labeling for $[AuBr_4]^-$ and β-CD in the solid-state superstructure of $KAuBr_4$⊂β-CD. Distances between the selected atoms are presented in Supplementary Table 1.

Supplementary Table 1 | **Intermolecular short distances[a] (Å) between $[AuBr_4]^-$ and the inner protons H-5 and H-6 in each of the seven glucose subunits of β-CD**

Distances[a] / Å	Unit 1	Unit 2	Unit 3	Unit 4	Unit 5	Unit 6	Unit 7
Br1/Br2–H6	3.8–4.2	3.4–4.1	3.2–4.9	4.1–4.2	3.6–3.7	3.5–4.4	3.4
Br1/Br2–H5	3.7	4.6–4.8	3.6	3.3–3.5	3.5	3.6–4.7	3.1
Br3/Br4–H6	4.8	4.4–4.9	4.7	N[b]	4.5	4.8–4.9	4.6
Br3/Br4–H5	3.6	4.4–4.5	3.3	3.3	3.4	3.3–4.7	3.0

[a] Short distance means the distance between two atoms $<$ 5 Å. [b] N means no interaction between two atoms.

(2) Crystal superstructure of $HAuBr_4$•DBC⊂2β-CD

(a) Method. Two molar equivalents of β-CD (500 μL, 20 mM) were added to an aqueous $KAuBr_4$ (500 μL, 10 mM) solution containing 1 M HBr. The resulting solution was added to two 1 mL tubes with volumes of 0.20 and 0.45 mL. The tubes were placed in one 20 mL vial containing DBC (100 μL). The vial was sealed with a cap. Slow vapor diffusion of DBC into the mixture of $KAuBr_4$ and β-CD over the period of 3 days yielded brown single crystals. A suitable crystal was selected and mounted on a MITIGEN holder with Paratone oil on an XtaLAB Synergy, Single source at home/near, HyPix diffractometer. The crystal was kept at 100.15 K during the data collection. Using Olex2,[1] the structure was solved with the ShelXT[2] structure solution program using Intrinsic Phasing and refined with the XL[3] refinement package employing Least Squares Minimization. Crystallographic images were produced using Mercury 4.3.0. Distances were measured employing Mercury 4.3.0. The solid-state superstructure of $HAuBr_4$•DBC⊂2β-CD is shown in Fig. 3 and Supplementary Fig. 3.

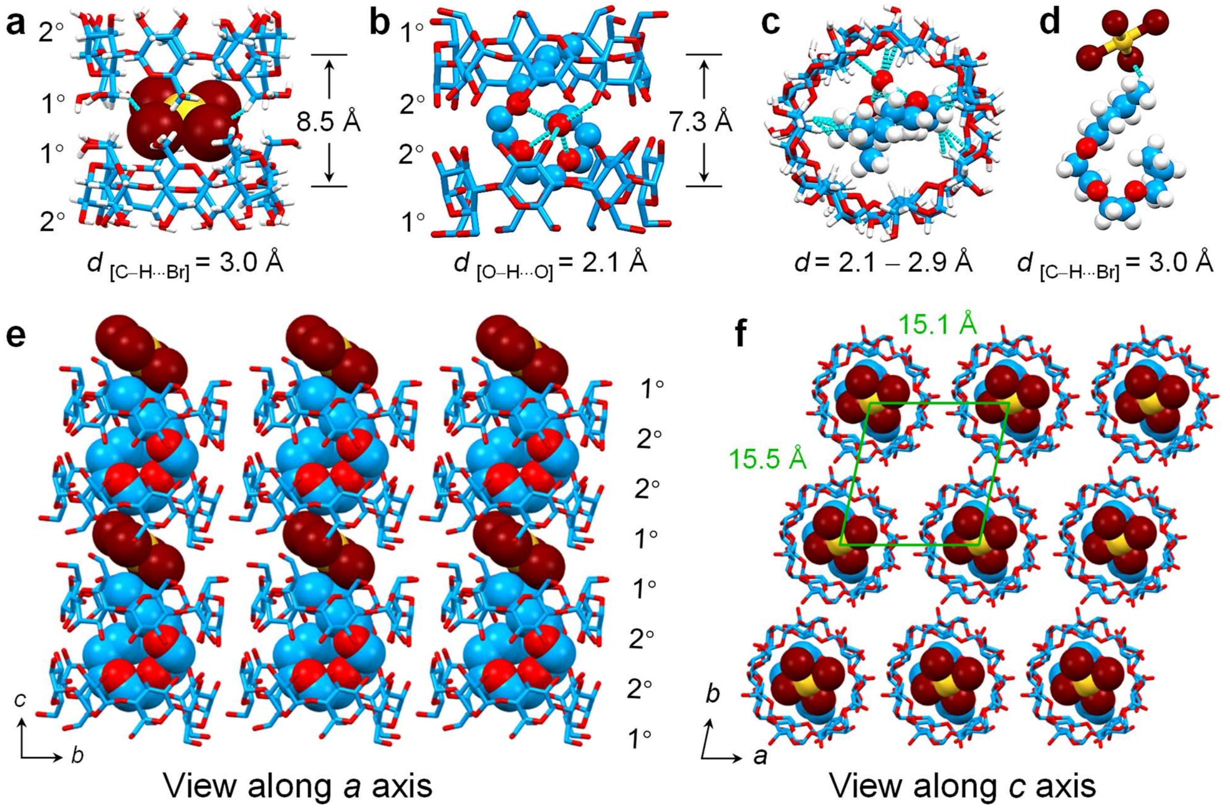

Supplementary Figure 3 | X-Ray single-crystal superstructure of the $HAuBr_4$•DBC⊂2β-CD cocrystal. (a) Capped-stick and space-filling representation of the solid-state superstructure of $[AuBr_4]^-$⊂2β-CD, showing that $[AuBr_4]^-$ is located between the primary faces of the two β-CD tori. (b) Ball- and capped-stick representation of the solid-state superstructure of DBC•H_2O⊂2β-CD, showing that DBC is located between the secondary faces of the two β-CD tori. (c) The top view of the solid-state superstructure of DBC•H_2O⊂2β-CD, showing noncovalent interactions between DBC, H_2O and two β-CD tori. (d) Ball-and-stick representation of the solid-state superstructure and noncovalent interactions between $[AuBr_4]^-$ and DBC. (e) Capped-stick and space-filling representation of the packing of $HAuBr_4$•DBC⊂2β-CD in the *b*-*c* plane, in which the β-CD tori form continuous nanotubes extending along the *c* axis occupied by alternating DBC and $[AuBr_4]^-$ anions. (f) Capped-stick and space-filling representation of the packing of $HAuBr_4$•DBC⊂2β-CD in the *a*-*b* plane, showing relative dispositions of adjacent one-dimensional nanotubes. The "1°" represents the primary face and the "2°" represents the secondary face of β-CD. Solvent molecules have been omitted for the sake of clarity. H white, C skyblue, O red, Br brown, Au yellow.

(b) Crystal Parameters. 2($C_{42}H_{70}O_{35}$)•$HAuBr_4$•$C_{12}H_{26}O_3$•14(H_2O). *Mr* =3258.11. Brown needle (0.1 × 0.01 × 0.01 mm^3). Triclinic, space group *P*1 (no. 1), a = 15.11370(18), b = 15.50618(18), c = 15.7047(2) Å, α = 88.8635(10), β = 81.9417(10), γ = 77.0250(10)°, V = 3550.84(8) Å^3, Z = 1, T = 100.15 K, μ(CuKα) = 4.205 mm^{-1}, *Dcalc* = 1.524 g/mm^3, 136295 reflections measured (7.422 ≤ 2Θ ≤ 160.428), 27552 unique (R_{int} = 0.0665, R_{sigma} = 0.0394) which were used in all calculations. The final R_1 was 0.0818 ($I > 2\sigma(I)$) and wR_2 was 0.2238 (all data).

(c) Refinement and solvent treatment details. Enhanced rigid-bond restraint[4] was applied to partial atoms. The solvent-masking procedure implemented in Olex2 was used to remove the electronic contribution of solvent molecules from the refinement. As the exact solvent content is unknown, only the atoms used in the refinement model and protons that were added for charge balance are reported in the formula here. Total solvent accessible volume / cell = 253.6 Å^3 [7.1%], Total electron count / cell = 91.9.

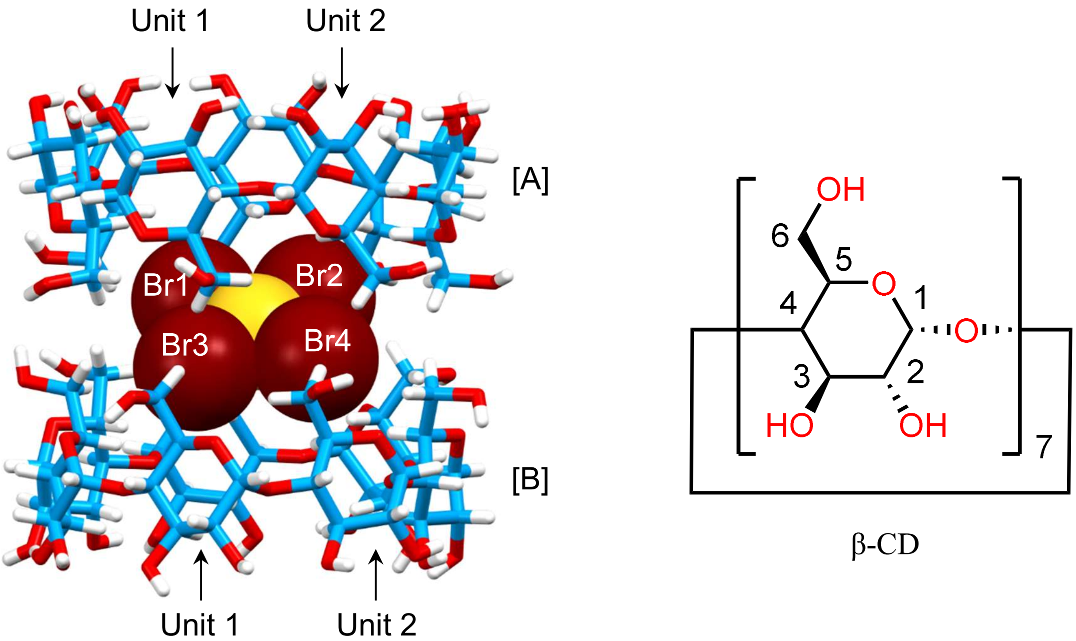

Supplementary Figure 4 | Labeling for $[AuBr_4]^-$ and β-CD in the solid-state superstructure of $HAuBr_4$•DBC⊂2β-CD. The top β-CD is labeled as [A], and the bottom β-CD is labeled as [B]. Distances between the selected atoms are presented in Supplementary Table 2.

Supplementary Table 2 | Intermolecular short distances[a] (Å) between $[AuBr_4]^-$ and the inner protons H-5 and H-6 in each of the seven glucose subunits of the two β-CD tori (A and B)

Distances[a] / Å	Unit 1	Unit 2	Unit 3	Unit 4	Unit 5	Unit 6	Unit 7
Br1/Br2–[A]H6	N[b]	N[b]	3.4–3.7	3.3–3.8	4.5	3.2–3.4	3.5–4.3
Br1/Br2–[A]H5	N[b]	4.9	3.4	3.2	4.7–4.9	3.5	3.5
Br1/Br2–[B]H6	N[b]	N[b]	N[b]	3.0–3.8	3.3–4.2	3.0–3.2	4.8
Br3/Br4–[B]H6	4.2–4.4	3.3–4.9	3.2–3.3	4.4–4.8	N[b]	4.3–4.6	3.3–3.5
Br3/Br4–[B]H5	4.1	3.5–4.8	3.2	4.4	4.9	3.7	3.4
Br3/Br4–[A]H6	3.3–4.7	3.3–3.5	4.1	N[b]	N[b]	N[b]	3.5–3.6

[a] Short distance means the distance between two atoms < 5 Å. [b] N means no interaction between two atoms.

(3) Crystal superstructure of $HAuBr_4$•2($i$$Pr_2O$)⊂2β-CD (Cocrystal A)

(a) Method. Two molar equivalents of β-CD (500 μL, 20 mM) were added to an aqueous $KAuBr_4$ (500 μL, 10 mM) solution containing 1 M HBr. $i$$Pr_2O$ (20 μL) was layered on top of the mixture of $KAuBr_4$ and β-CD. High-quality brown crystals were obtained after liquid-liquid diffusion for 12 h. A suitable crystal was selected and mounted on a MITIGEN holder with Paratone oil on an XtaLAB Synergy, Single source at offset/far, HyPix diffractometer. The crystal was kept at 99.9 K during the data collection. Using Olex2,[1] the structure was solved with the ShelXT[2] structure solution program using Intrinsic Phasing and refined with the XL[3] refinement package employing Least Squares Minimization. Crystallographic images were produced using Mercury 4.3.0. Distances were measured employing Mercury 4.3.0. The solid-state superstructure of $HAuBr_4$•2($i$$Pr_2O$)⊂2β-CD is shown in Fig. 4b and Supplementary Fig. 5.

(b) Crystal Parameters. 2($C_{42}H_{70}O_{35}$)•$HAuBr_4$•2($C_6H_{14}O$)•8(H_2O). Mr = 3136.04. Brown needle (0.1 × 0.02 × 0.01 mm^3). Triclinic, space group $P1$ (no. 1), a = 15.1182(8), b = 15.3034(9), c = 15.5384(8) Å, α = 87.704(4), β = 81.920(4), γ = 76.368(5)°, V = 3458.9(3) Å^3, Z = 1, T = 99.9(3) K, μ(MoKα) = 2.322 mm^{-1}, $Dcalc$ = 1.506 g/cm^3, 63929 reflections measured (4.082° ≤ 2Θ ≤ 60.818°), 27941 unique (R_{int} = 0.2267, R_{sigma} = 0.3202) which were used in all calculations. The final R_1 was 0.1437 (I > 2σ(I)) and wR_2 was 0.3894 (all data).

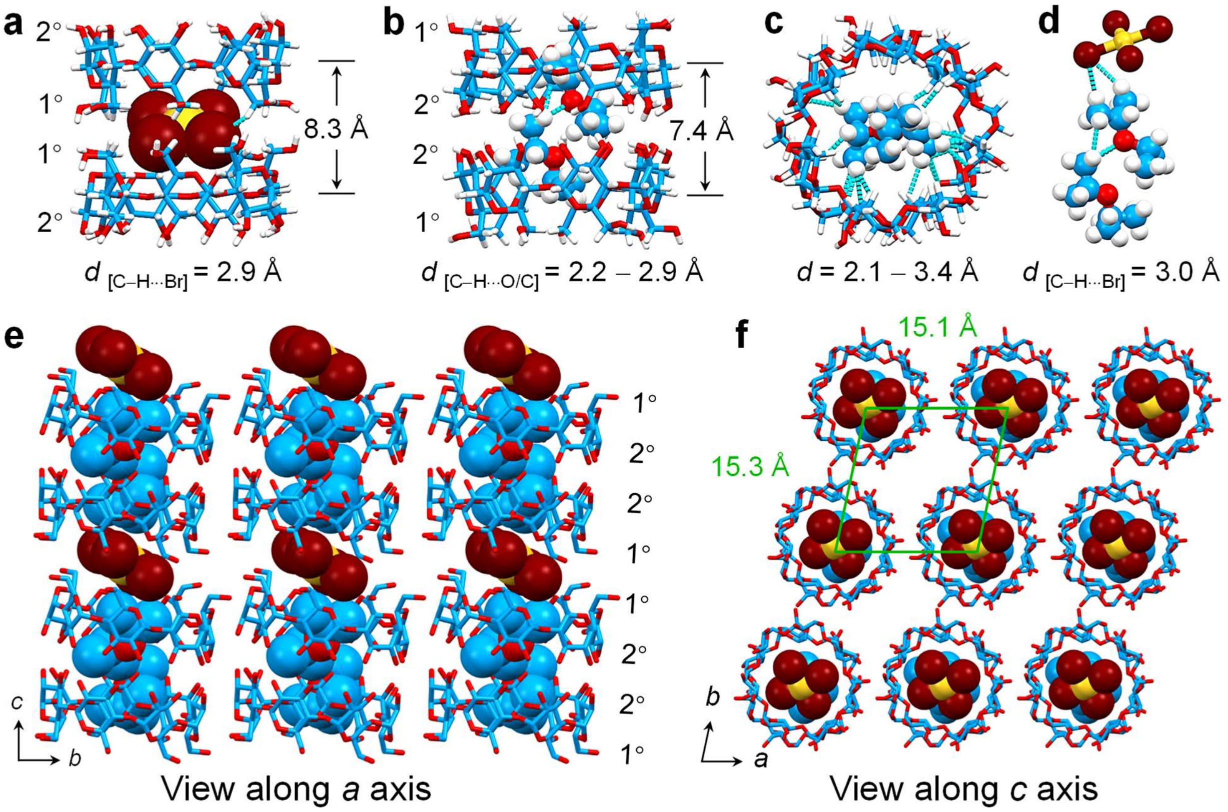

Supplementary Figure 5 | X-Ray single-crystal superstructure of the $HAuBr_4$•2(*i*Pr_2O)⊂2β-CD cocrystal. (a) Capped-stick and space-filling representation of the solid-state superstructure of $[AuBr_4]^-$⊂2β-CD, showing that $[AuBr_4]^-$ is located between the primary faces of the two β-CD tori. (b) Ball- and capped-stick representation of the solid-state superstructure of 2(*i*Pr_2O)⊂2β-CD, showing that two *i*Pr_2O are located between the secondary faces of the two β-CD tori. (c) The top view of the solid-state superstructure of 2(*i*Pr_2O)⊂2β-CD, showing the noncovalent interactions between two *i*Pr_2O and two β-CD tori. (d) Ball-and-stick representation of the solid-state superstructure and noncovalent interactions between two *i*Pr_2O and $[AuBr_4]^-$. (e) Capped-stick and space-filling representation of the packing of $HAuBr_4$•2(*i*Pr_2O)⊂2β-CD in the *b*-*c* plane, in which the β-CD tori form continuous nanotubes extending along the *c* axis occupied by alternating *i*Pr_2O and $[AuBr_4]^-$. (f) Capped-stick and space-filling representation of the packing of $HAuBr_4$•2(*i*Pr_2O)⊂2β-CD in the *a*-*b* plane, showing the relative dispositions of adjacent one-dimensional nanotubes. The "1° " represents the primary face and the "2° " represents the secondary face of β-CD. Solvent molecules have been omitted for the sake of clarity. H white, C skyblue, O red, Br brown, Au yellow.

(c) Refinement and solvent treatment details. Enhanced rigid-bond restraint[4] was applied to partial atoms. The solvent-masking procedure implemented in Olex2 was used to remove the electronic contribution of solvent molecules from the refinement. As the exact solvent content is unknown, only the atoms used in the refinement model and protons that were added for charge balance are reported in the formula here. Total solvent accessible volume / cell = 393.6 $Å^3$ [11.4%], Total electron count / cell = 145.2.

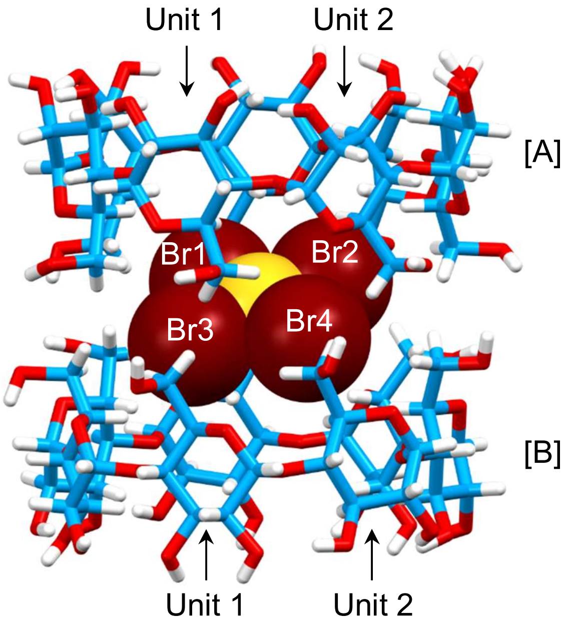

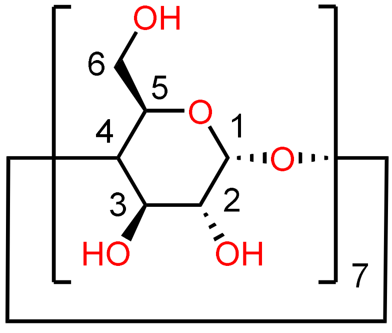

β-CD

Supplementary Figure 6 | Labeling for $[AuBr_4]^-$ and β-CD in the solid-state superstructure of $HAuBr_4$•2($i$$Pr_2O$)⊂2β-CD. The top β-CD is labeled as [A], and the bottom β-CD is labeled as [B]. Distances between the selected atoms are presented in Supplementary Table 3.

Supplementary Table 3 | Intermolecular short distances[a] (Å) between $[AuBr_4]^-$ and the inner protons H-5 and H-6 in each of the seven glucose subunits of the two β-CD tori (A and B)

Distances[a] / Å	Unit 1	Unit 2	Unit 3	Unit 4	Unit 5	Unit 6	Unit 7
Br1/Br2–[A]H6	N[b]	4.3–4.8	3.3–4.0	3.4–4.8	4.5–4.6	3.2–3.4	4.0–4.4
Br1/Br2–[A]H5	N[b]	4.4	3.3	3.6	4.4	3.7	3.6
Br1/Br2–[B]H6	N[b]	N[b]	4.2	3.2–3.4	3.1–4.5	3.2–3.3	N[b]
Br3/Br4–[B]H6	4.2–4.9	3.3–3.7	3.3–3.7	4.9	N[b]	3.7–4.3	3.3–3.5
Br3/Br4–[B]H5	4.5–4.8	3.2	3.4	4.8	4.9	3.5	3.2
Br3/Br4–[A]H6	3.3–4.3	2.9–3.7	4.8	N[b]	N[b]	N[b]	3.1–3.3

[a] Short distance means the distance between two atoms ＜5 Å. [b] N means no interaction between two atoms.

(4) Crystal superstructure of $0.5(HAuBr_4)\subset 2\beta$-CD (Cocrystal B)

(a) Method. Two molar equivalents of β-CD (500 μL, 20 mM) were added to an aqueous $KAuBr_4$ (500 μL, 10 mM) solution containing 1 M HBr in a 3 mL vial without the cap. *i*Pr_2O (20 μL) was layered carefully on top of the mixture of $KAuBr_4$ and β-CD. High-quality brown crystals were obtained after liquid-liquid diffusion for 3 days. A suitable crystal was selected and mounted on a MITIGEN holder with Paratone oil on an XtaLAB Synergy, Single source at offset/far, HyPix diffractometer. The crystal was kept at 100.0 K during the data collection. Using Olex2,[1] the structure was solved with the ShelXT[2] structure solution program using Intrinsic Phasing and refined with the XL[3] refinement package employing Least Squares Minimization. Crystallographic images were produced using Mercury 4.3.0. Distances were measured employing Mercury 4.3.0. The solid-state superstructure of $0.5(HAuBr_4)\subset 2\beta$-CD is shown in Fig. 4c and Supplementary Fig. 7.

(b) Crystal Parameters. $2(C_{42}H_{70}O_{35})\bullet 0.5(HAuBr_4)\bullet 23(H_2O)$. M_r = 2943.12. Brown needle (0.05 × 0.01 × 0.01 mm^3). Monoclinic, space group $P2_1$ (no. 4), a = 15.7110(7), b = 24.3931(7), c = 18.9812(7) Å, α = 90.000, β = 108.544(4), γ = 90.000°, V = 6896.7(5) Å^3, Z = 2, T= 100.0(3) K, μ(Mo Kα)= 1.228 mm^{-1}, *Dcalc* = 1.417 g/cm^3, 168898 reflections measured (4.066° ≤ 2Θ ≤ 50.696°), 25231 unique (R_{int} = 0.1822, R_{sigma} = 0.1014) which were used in all calculations. The final R_1 was 0.1150 ($I > 2\sigma(I)$) and wR_2 was 0.3237 (all data).

(c) Refinement and solvent treatment details. Enhanced rigid-bond restraint[4] was applied to partial atoms. The solvent-masking procedure implemented in Olex2 was used to remove the electronic contribution of solvent molecules from the refinement. As the exact solvent content is unknown, only the atoms used in the refinement model and protons that were added for charge balance are reported in the formula here. Total solvent accessible volume / cell = 808.2 Å^3 [11.7%], Total electron count / cell = 217.1.

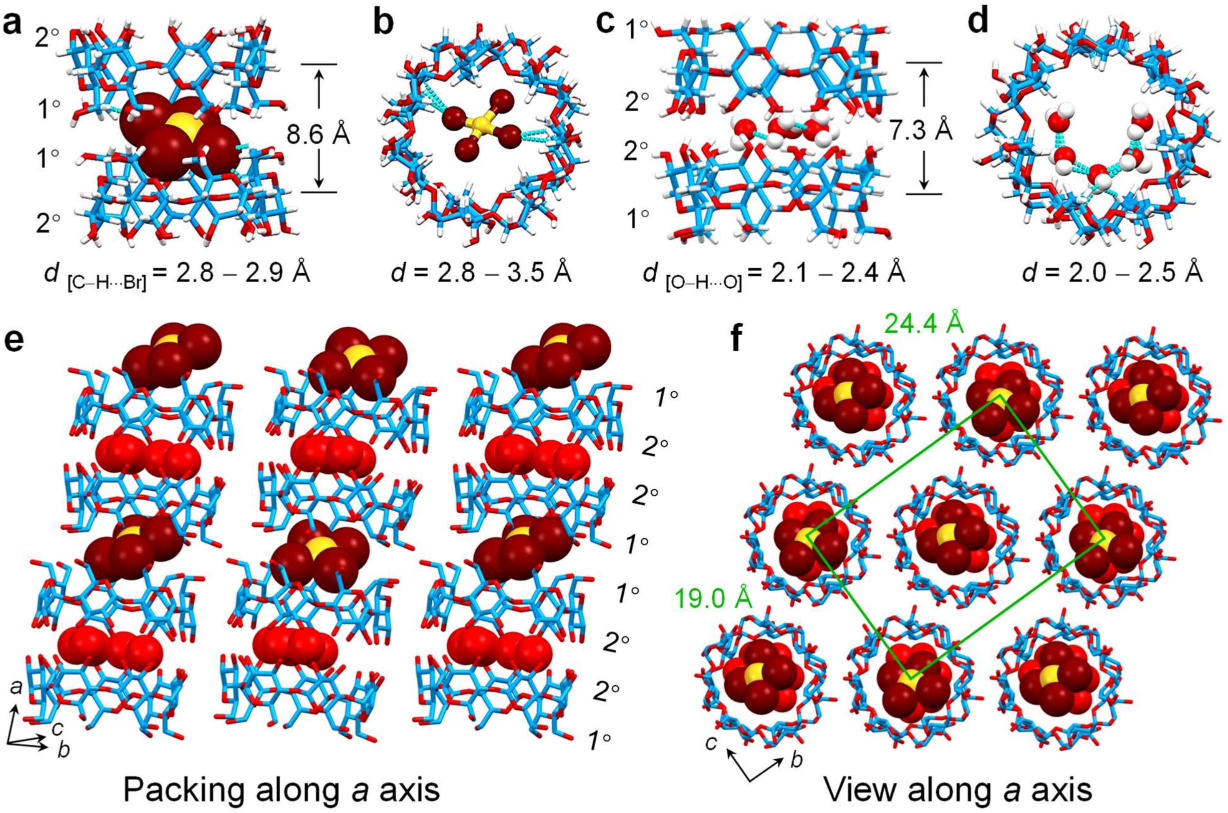

Supplementary Figure 7 | X-Ray single-crystal superstructure of the 0.5($HAuBr_4$)⊂2β-CD cocrystal, in which the occupation of $[AuBr_4]^-$ is 50%. (a) Capped-stick and space-filling representation of the solid-state superstructure of 0.5$[AuBr_4]^-$⊂2β-CD, showing that $[AuBr_4]^-$ is located between the primary faces of the two β-CD tori. (b) The top view of the solid-state superstructure of 0.5$[AuBr_4]^-$⊂2β-CD, showing the noncovalent interactions between $[AuBr_4]^-$ and two β-CD tori. (c) Ball- and capped-stick representation of the solid-state superstructure between H_2O and β-CD tori, showing that the five H_2O molecules are located between the secondary faces of the two β-CD tori. (d) The top view of the solid-state superstructure between H_2O and β-CD tori, showing the noncovalent interactions between the five H_2O molecules and two β-CD tori. (e) Capped-stick and space-filling representation of the packing of 0.5($HAuBr_4$)⊂2β-CD extending along the *a* axis, in which the β-CD tori form continuous nanotubes occupied by alternating H_2O and $[AuBr_4]^-$. (f) Capped-stick and space-filling representation of the packing of 0.5($HAuBr_4$)⊂2β-CD in the *b*-*c* plane, showing relative dispositions of adjacent one-dimensional β-CD nanotubes. The "1°" represents the primary face and the "2°" represents the secondary face of β-CD. Solvent molecules have been omitted for the sake of clarity. H white, C skyblue, O red, Br brown, Au yellow.

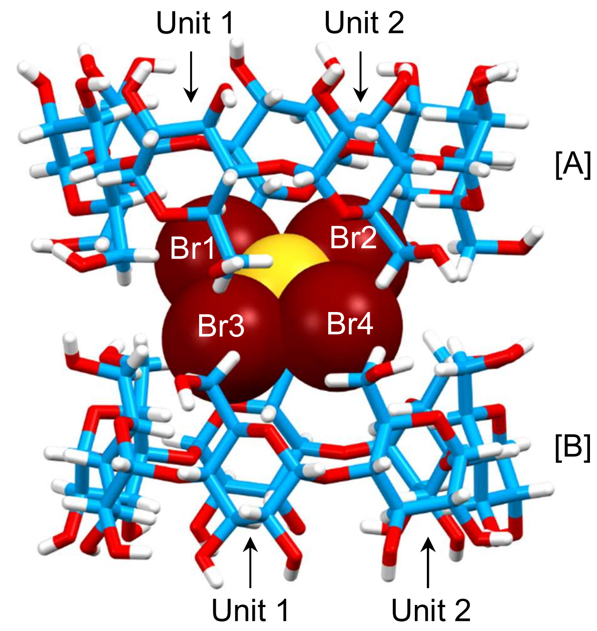

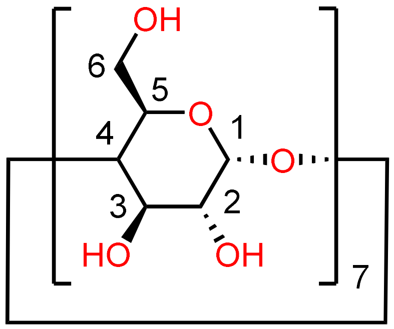

β-CD

Supplementary Figure 8 | Labeling for $[AuBr_4]^-$ and β-CD in the solid-state superstructure of $0.5(HAuBr_4)\subset 2\beta$-CD. The top β-CD is labeled as [A], and the bottom β-CD is labeled as [B]. Distances between the selected atoms are presented in Supplementary Table 4.

Supplementary Table 4 | Intermolecular short distances[a] (Å) between $[AuBr_4]^-$ and the inner protons H-5 and H-6 in each of the seven glucose subunits of the two β-CD tori (A and B)

Distances[a] / Å	Unit 1	Unit 2	Unit 3	Unit 4	Unit 5	Unit 6	Unit 7
Br1/Br2–[A]H6	N[b]	4.9	3.1–3.6	3.0–3.4	3.4–4.5	3.5	3.4–3.8
Br1/Br2–[A]H5	4.8	4.9	3.2	2.9	4.1–4.4	3.1	3.2
Br1/Br2–[B]H6	N[b]	N[b]	N[b]	3.4–4.2	3.2–4.5	3.2–3.3	N[b]
Br3/Br4–[B]H6	4.7	4.6	3.4–3.8	4.1–4.4	4.6–4.7	3.5–4.0	2.8–3.1
Br3/Br4–[B]H5	N[b]	4.6	3.9	4.2	4.5	3.4	3.6
Br3/Br4–[A]H6	3.1–4.4	3.4–3.6	3.9–4.7	N[b]	N[b]	N[b]	3.2–3.4

[a] Short distance means the distance between two atoms < 5 Å. [b] N means no interaction between two atoms.

Supplementary Table 5 | Crystallographic data for the complexes formed between β-CD and $[AuBr_4]^-$ before and after adding additives

Complex	$KAuBr_4 \subset \beta$-CD	$KAuBr_4 \bullet$DBC $\subset 2\beta$-CD	$KAuBr_4 \bullet 2(i Pr_2O)$ $\subset 2\beta$-CD	$0.5(HAuBr_4) \subset 2\beta$-CD
Empirical formula	$C_{42}H_{70}O_{35} \bullet KAuBr_4 \bullet 4(H_2O)$	$2(C_{42}H_{70}O_{35}) \bullet HAuBr_4 \bullet C_{12}H_{26}O_3 \bullet 14(H_2O)$	$2(C_{42}H_{70}O_{35}) \bullet HAuBr_4 \bullet 2(C_6H_{14}O) \bullet 8(H_2O)$	$2(C_{42}H_{70}O_{35}) \bullet 0.5(HAuBr_4) \bullet 23(H_2O)$
Formula weight	1762.75	3258.11	3136.04	2943.12
T / K	100.07(16)	100.15	99.9(3)	100.0(3)
Crystal system	monoclinic	triclinic	triclinic	monoclinic
Space group	$P2_1$	$P1$	$P1$	$P2_1$
a / Å	14.90540(10)	15.11370(18)	15.1182(8)	15.7110(7)
b / Å	15.43740(10)	15.50618(18)	15.3034(9)	24.3931(7)
c / Å	14.91910(10)	15.7047(2)	15.5384(8)	18.9812(7)
α / °	90	88.8635(10)	87.704(4)	90
β / °	119.3100(10)	81.9417(10)	81.920(4)	108.544(4)
γ / °	90	77.0250(10)	76.368(5)	90
V / Å^3	2993.43(4)	3550.84(8)	3458.9(3)	6896.7(5)
Z	2	1	1	2
ρ_{calcd} / g cm^{-3}	1.956	1.524	1.506	1.417
μ / mm^{-1}	9.299	4.205	2.322	1.228
F (000)	1760	1686.0	1620.0	3088.0
goodness-of-fit on F^2	1.067	1.068	1.017	1.208
R_1 [$I > 2\sigma(I)$]	0.0270	0.0818	0.1437	0.1150
wR_2 [all data]	0.0711	0.2238	0.3894	0.3237
CCDC No.	2206843	2206844	2206845	2206846

Supplementary Note 2 ^{1}H NMR titration

^{1}H NMR Titrations were performed in D_2O containing 0.5 M DBr at 25 ˚C. Aliquots from a stock solution containing the appropriate amount of $KAuBr_4$, K_2PdBr_4, or K_2PtBr_4 were added sequentially to an NMR tube containing the β-CD or γ-CD, and a ^{1}H NMR spectrum was acquired after each addition. The titration isotherms were fitted to a 1:1 receptor-substrate binding model using Thordarson's equations[5] at http://app.supramolecular.org/bindfit/.

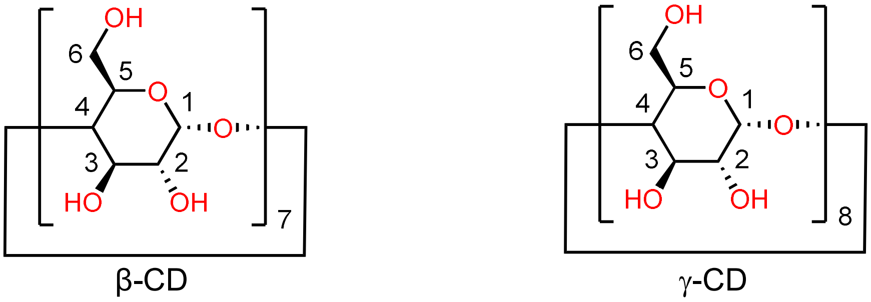

Supplementary Figure 9 | The structural formulas for β-CD and γ-CD with labels

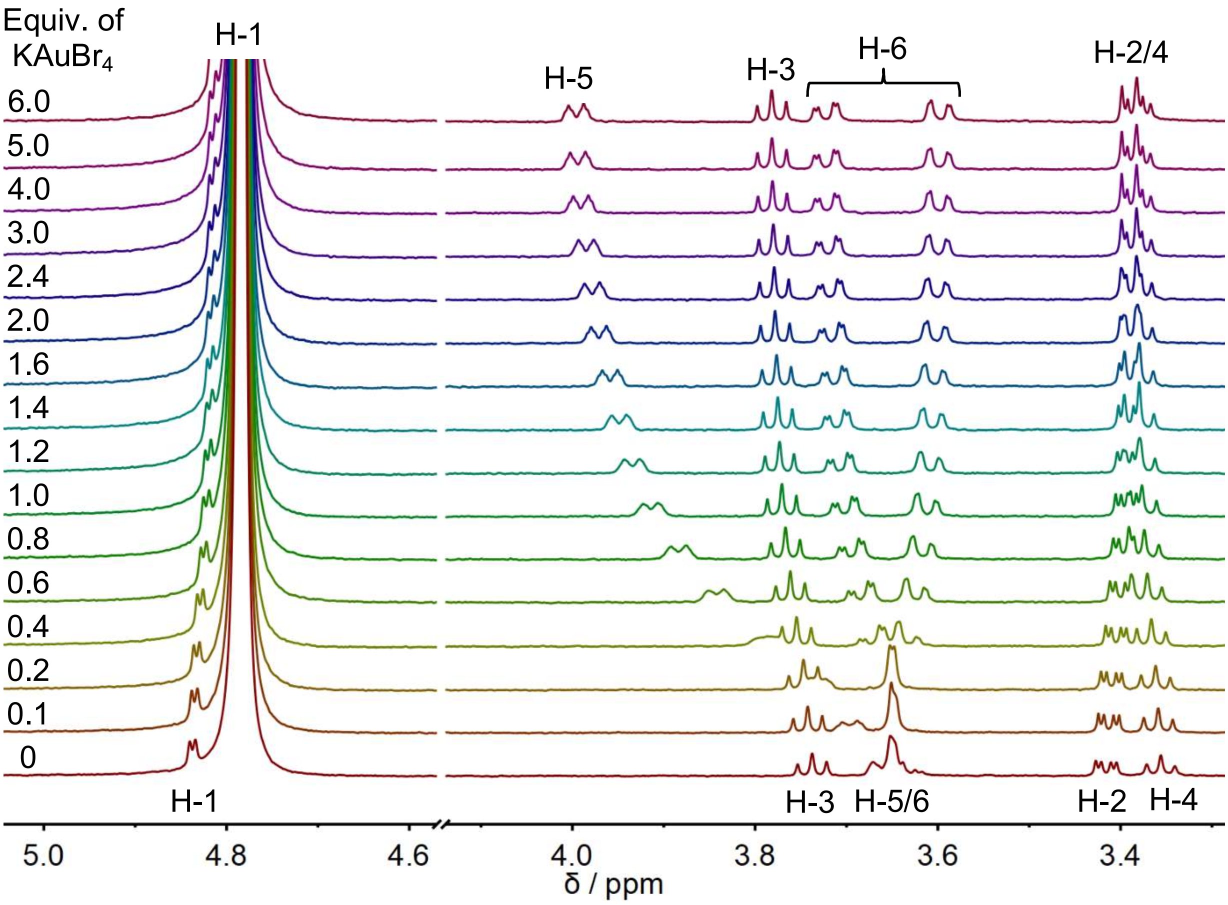

Supplementary Figure 10 | ^{1}H NMR Spectra (600 MHz, D_2O containing 0.5 M DBr, 25 ˚C) of β-CD (0.25 mM) titrated with $KAuBr_4$ (25 mM)

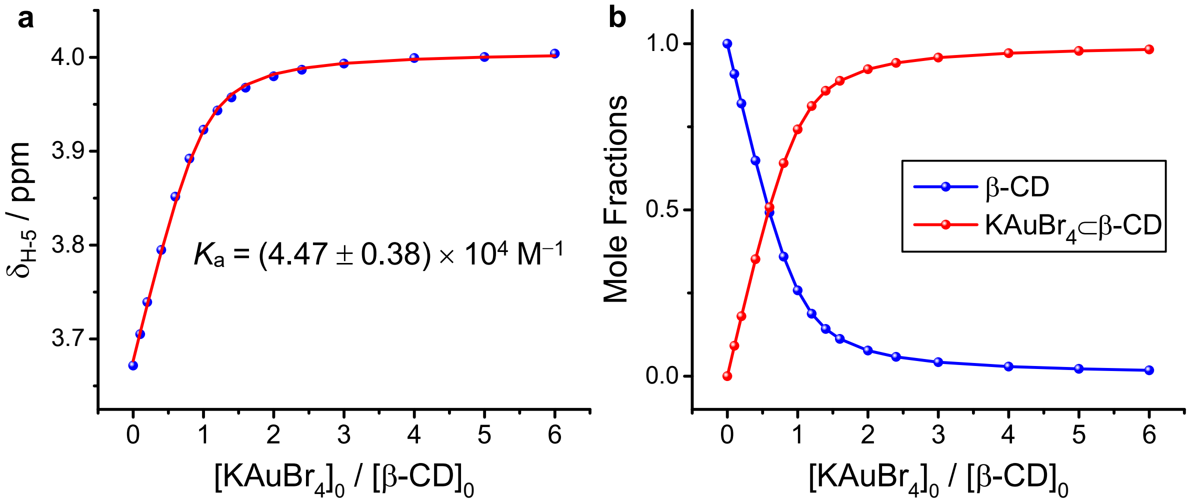

Supplementary Figure 11 | (a) Titration isotherm created by monitoring changes in the chemical shift of H-5 in β-CD, caused by the stepwise addition of $KAuBr_4$ at 25 ˚C. Red line is the result of curve fitting using a 1:1 receptor-substrate binding model. (b) Mole fractions are based on the fitting results, indicating that the concentration of the free β-CD undergoes a continuous decrease (blue trace), while the concentration of $KAuBr_4$⊂β-CD complex undergoes a continuous increase (red trace).

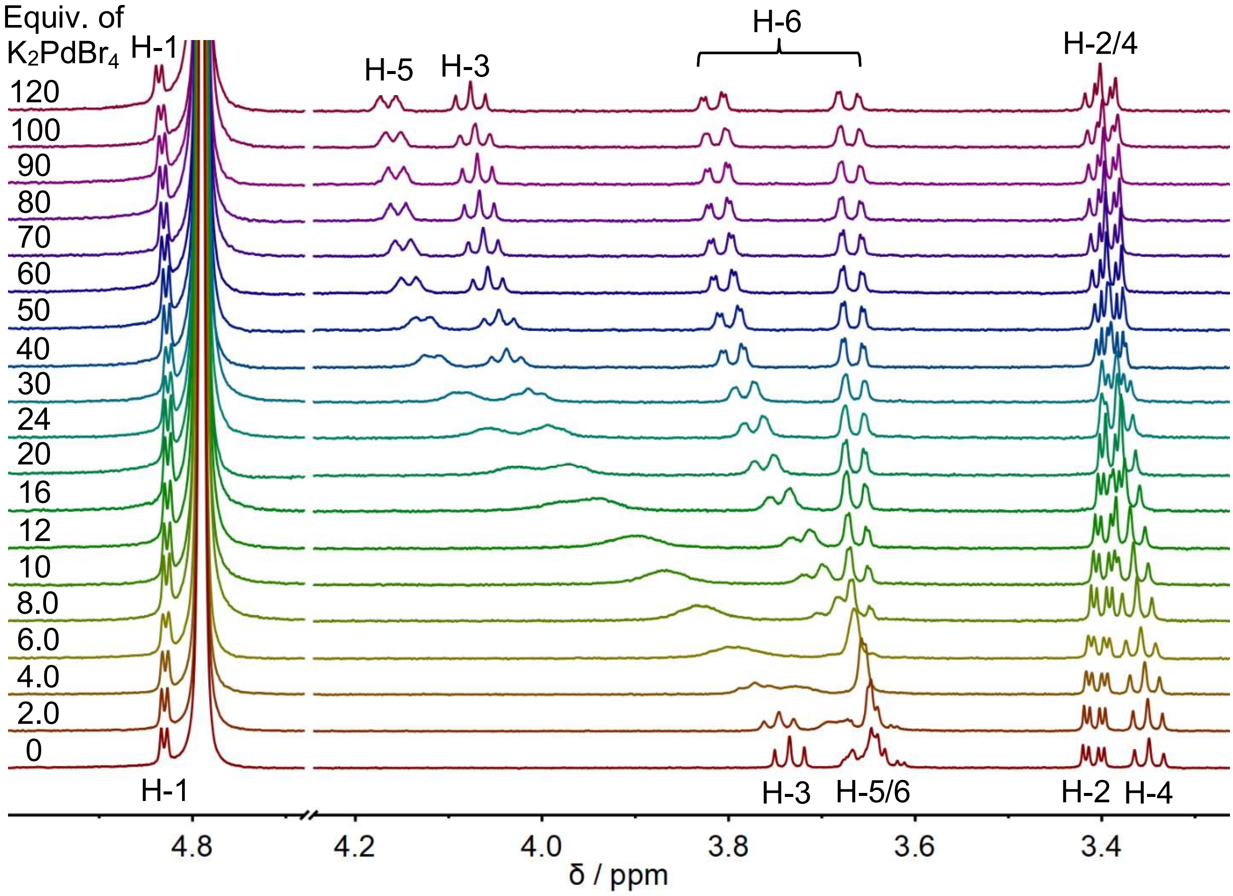

Supplementary Figure 12 | ^{1}H NMR Spectra (600 MHz, D_2O containing 0.5 M DBr, 25 ˚C) of β-CD (0.5 mM) titrated with K_2PdBr_4 (100 mM)

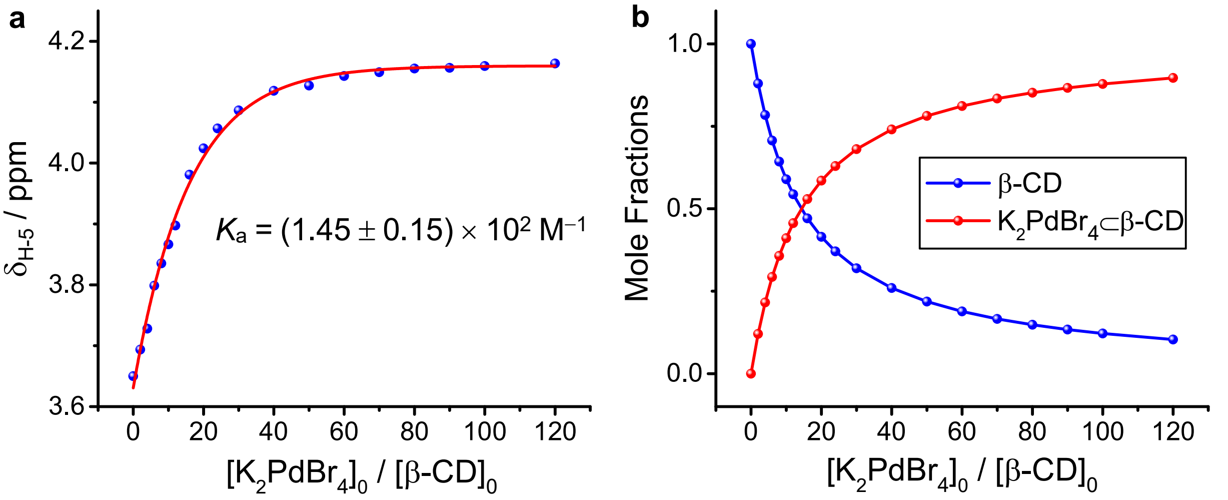

Supplementary Figure 13 | (a) Titration isotherm created by monitoring changes in the chemical shift of H-5 in β-CD, caused by the stepwise addition of K_2PdBr_4 at 25 ˚C. Red line is the result of curve fitting using a 1:1 receptor-substrate binding model. (b) Mole fractions are based on the fitting results, indicating that the concentration of the free β-CD undergoes a continuous decrease (blue trace), while the concentration of $K_2PdBr_4 \subset$β-CD complex undergoes a continuous increase (red trace).

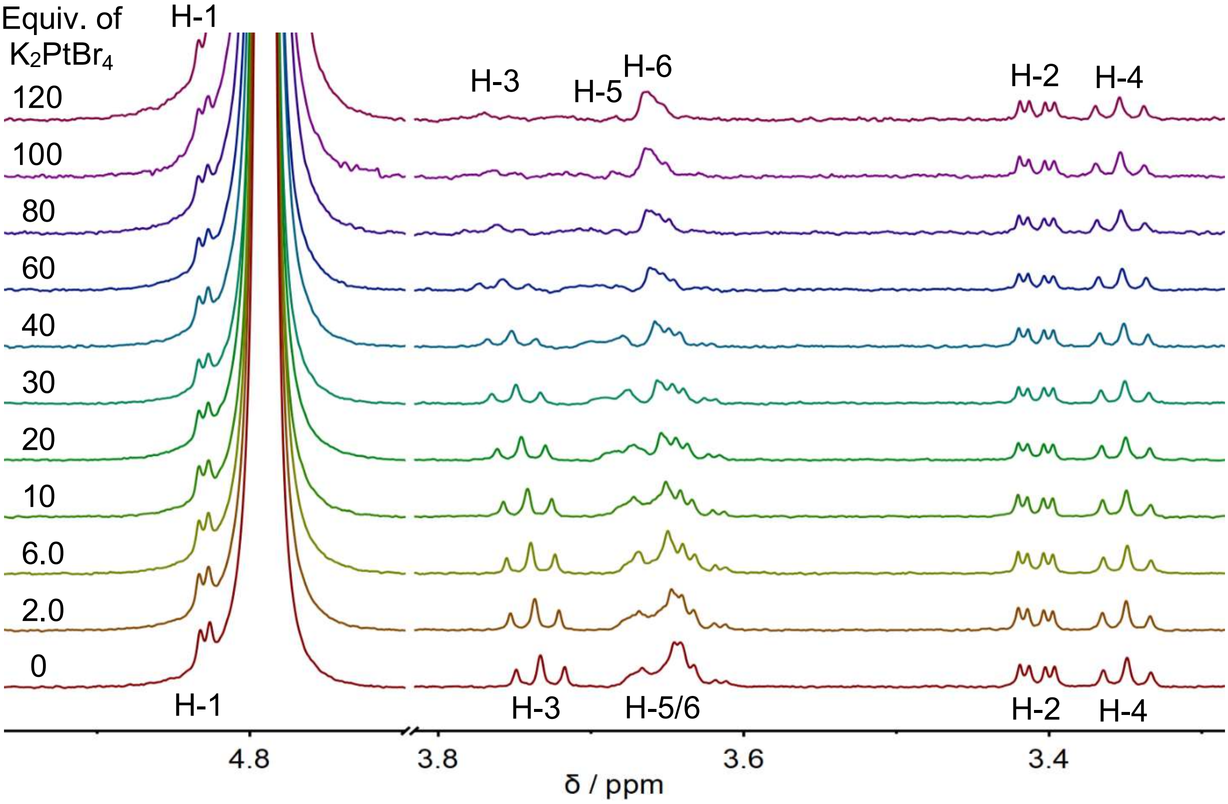

Supplementary Figure 14 | ^{1}H NMR Spectra (600 MHz, D_2O containing 0.5 M DBr, 25 ˚C) of β-CD (0.15 mM) titrated with a saturated K_2PtBr_4 (11.34 mM) aqueous solution. In order to avoid the error caused by volume dilution, we did not continue to increase the number of equivalents of the K_2PtBr_4 during the titration

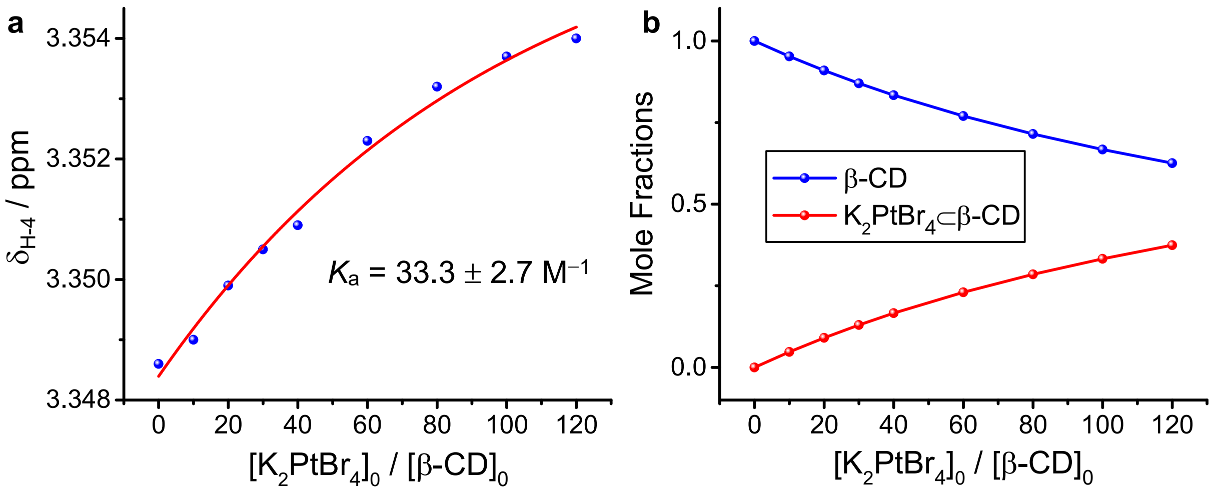

Supplementary Figure 15 | (a) Titration isotherm created by monitoring changes in the chemical shift of H-4 in β-CD, caused by the stepwise addition of K_2PtBr_4 at 25 ˚C. Red line is the result of curve fitting using a 1:1 receptor-substrate binding model. (b) Mole fractions are based on the fitting results, indicating that the concentration of the free β-CD undergoes a continuous decrease (blue trace), while the concentration of K_2PtBr_4⊂β-CD complex undergoes a continuous increase (red trace).

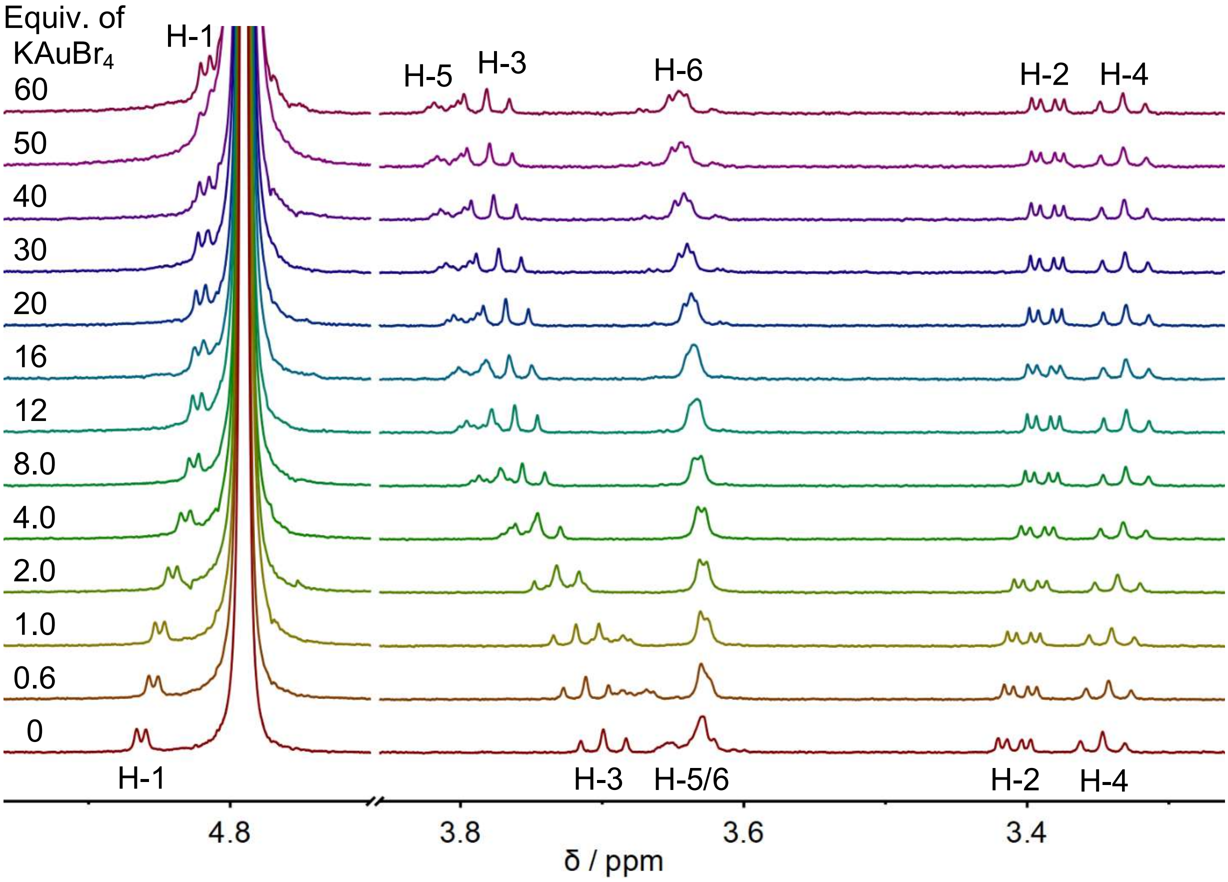

Supplementary Figure 16 | ^{1}H NMR Spectra (600 MHz, D_2O containing 0.5 M DBr, 25 ˚C) of γ-CD (0.25 mM) titrated with $KAuBr_4$ (100 mM)

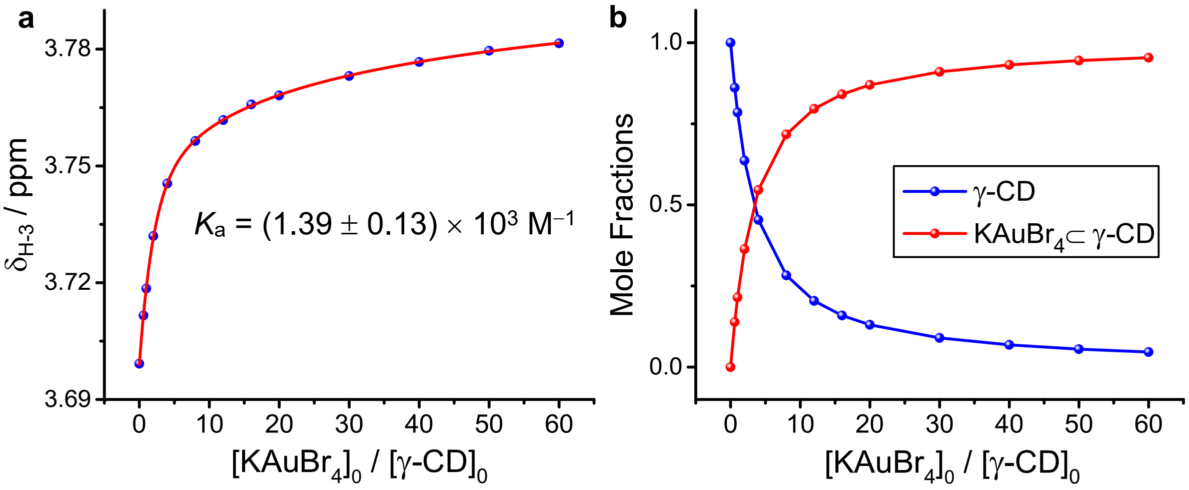

Supplementary Figure 17 | (a) Titration isotherm created by monitoring changes in the chemical shift of H-3 in γ-CD, caused by the stepwise addition of $KAuBr_4$ at 25 ˚C. Red line is the result of curve fitting using a 1:1 receptor-substrate binding model. (b) Mole fractions are based on the fitting results, indicating that the concentration of the free γ-CD undergoes a continuous decrease (blue trace), while the concentration of $KAuBr_4$⊂γ-CD complex undergoes a continuous increase (red trace).

Supplementary Note 3. High-resolution mass spectrometry

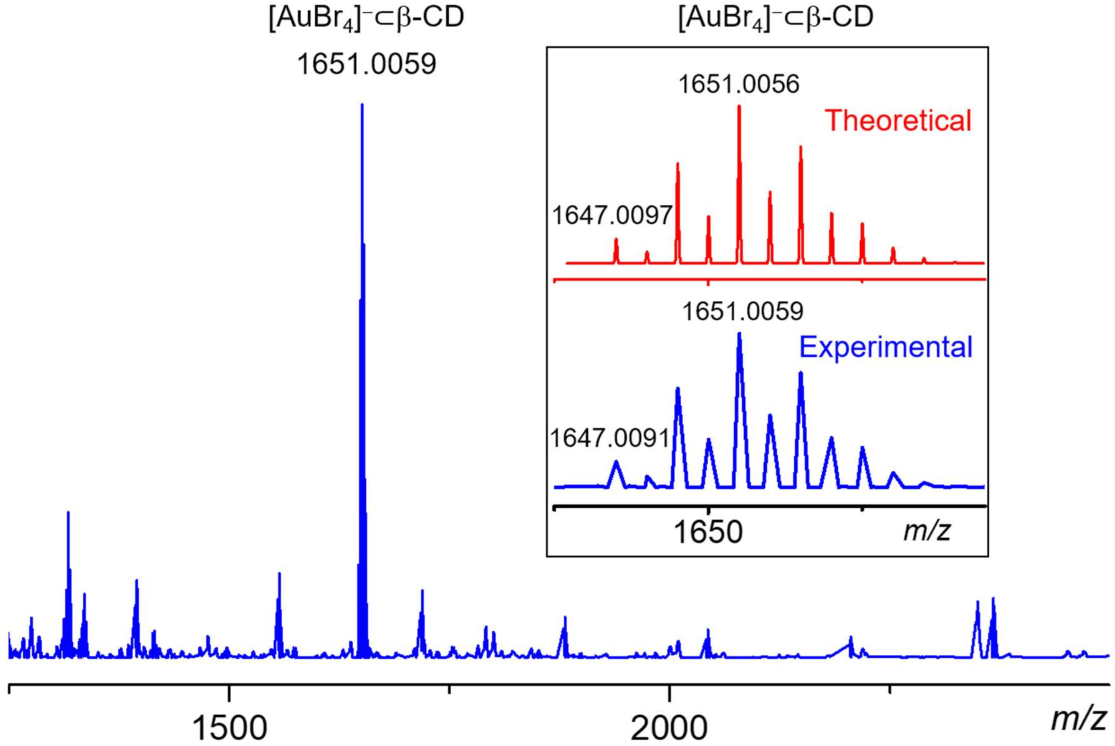

Supplementary Figure 18 | HRMS of $KAuBr_4$⊂β-CD. Inset shows the theoretical (red) and experimental (blue) isotope patterns for one negatively charged state of $KAuBr_4$⊂β-CD.

Supplementary Note 4. UV-Vis Absorption spectroscopy

The concentrations of $[AuBr_4]^-$ in aqueous solutions were determined by UV-Vis absorption spectroscopy employing the absorbance of $[AuBr_4]^-$ at λ = 381 or 253 nm. The intensity of the UV-Vis absorption band at λ = 381 nm correlates (Supplementary Fig. 19) linearly with the concentration of $[AuBr_4]^-$ over the range from 50 to 150 µM in H_2O with $R^2 = 0.999$. The intensity of the UV-Vis absorption band at λ = 253 nm correlates (Supplementary Fig. 20) linearly with the concentration of $[AuBr_4]^-$ over the range from 2 to 10 µM in H_2O with $R^2 = 0.999$.

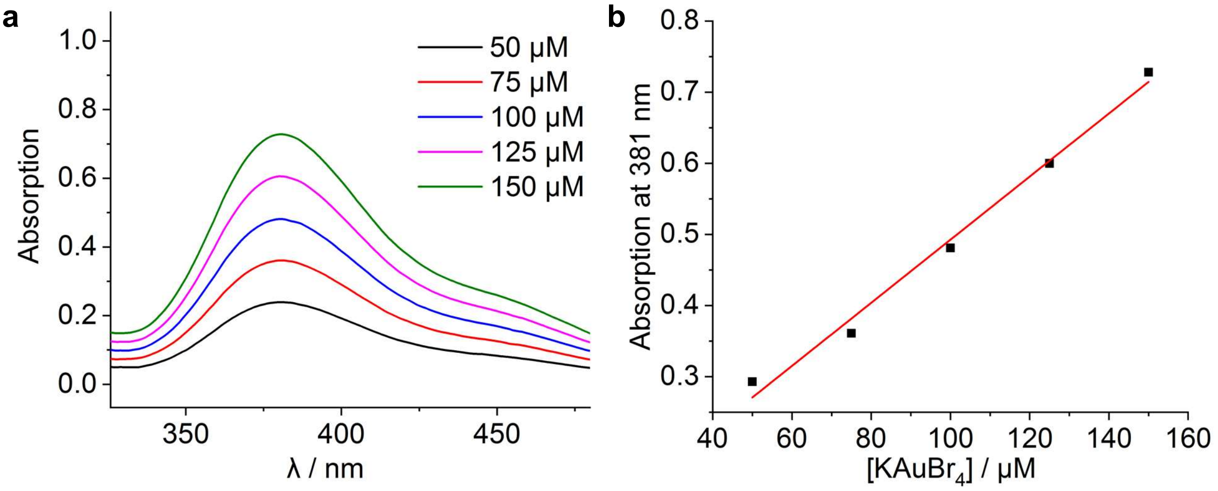

Supplementary Figure 19 | (a) UV-Vis Absorption spectra of $[AuBr_4]^-$ anions, recording over a range of concentrations from 50 to 150 µM in H_2O. (b) The intensity of the absorption band at λ = 381 nm correlates linearly with the concentration of $[AuBr_4]^-$ with $R^2 = 0.999$.

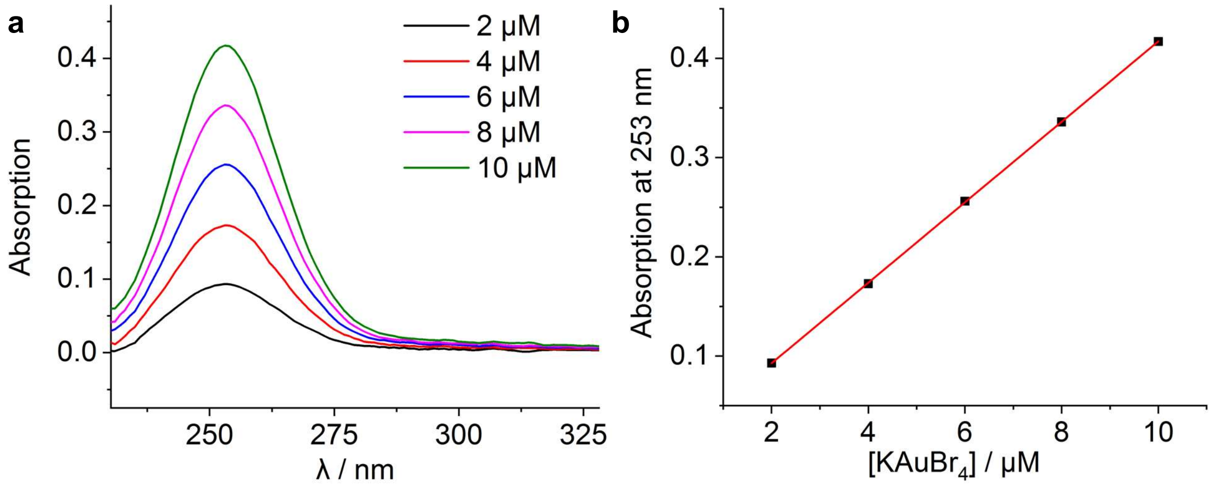

Supplementary Figure 20 | (a) UV-Vis Absorption spectra of $[AuBr_4]^-$ anions, recording over a range of concentrations from 2 to 10 µM in H_2O. (b) The intensity of the absorption band at λ = 253 nm correlates linearly with the concentration of $[AuBr_4]^-$ with $R^2 = 0.999$.

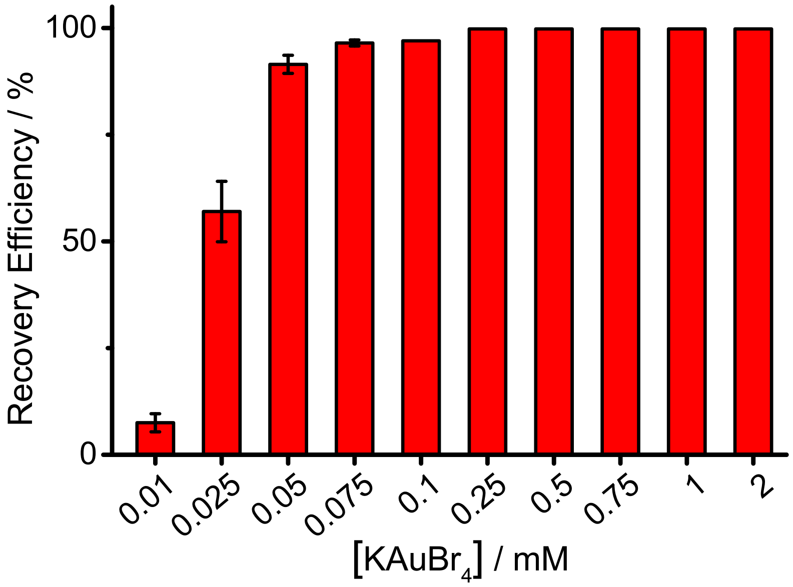

Supplementary Figure 21 | Effect of changes in concentration of $[AuBr_4]^-$ on gold-recovery efficiency, when adding 0.1% (v/v) of DBC to the aqueous solution of $KAuBr_4{\subset}\beta$-CD complex. The concentration of HBr was 1 M in all aqueous solutions. The error bar represents the standard deviation of two independent measurements.

Supplementary Note 5. Powder X-ray diffraction analysis

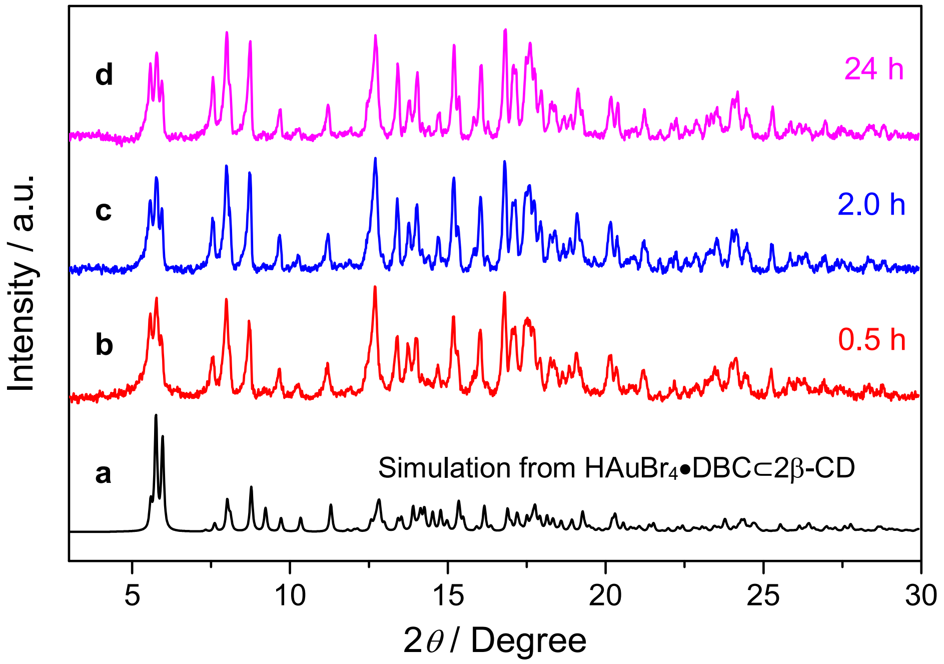

Supplementary Figure 22 | Powder X-ray diffraction patterns of microcrystals obtained by adding DBC to aqueous solutions of β-CD and $[AuBr_4]^-$ anion. (a) Simulation derived from the single-crystal X-ray crystallographic data for $HAuBr_4{\bullet}DBC{\subset}2\beta$-CD cocrystal. (b–d) The synthetic $HAuBr_4{\bullet}DBC{\subset}2\beta$-CD microcrystals after allowing them to stand in the mother liquid for 0.5, 2.0 and 24 h, respectively.

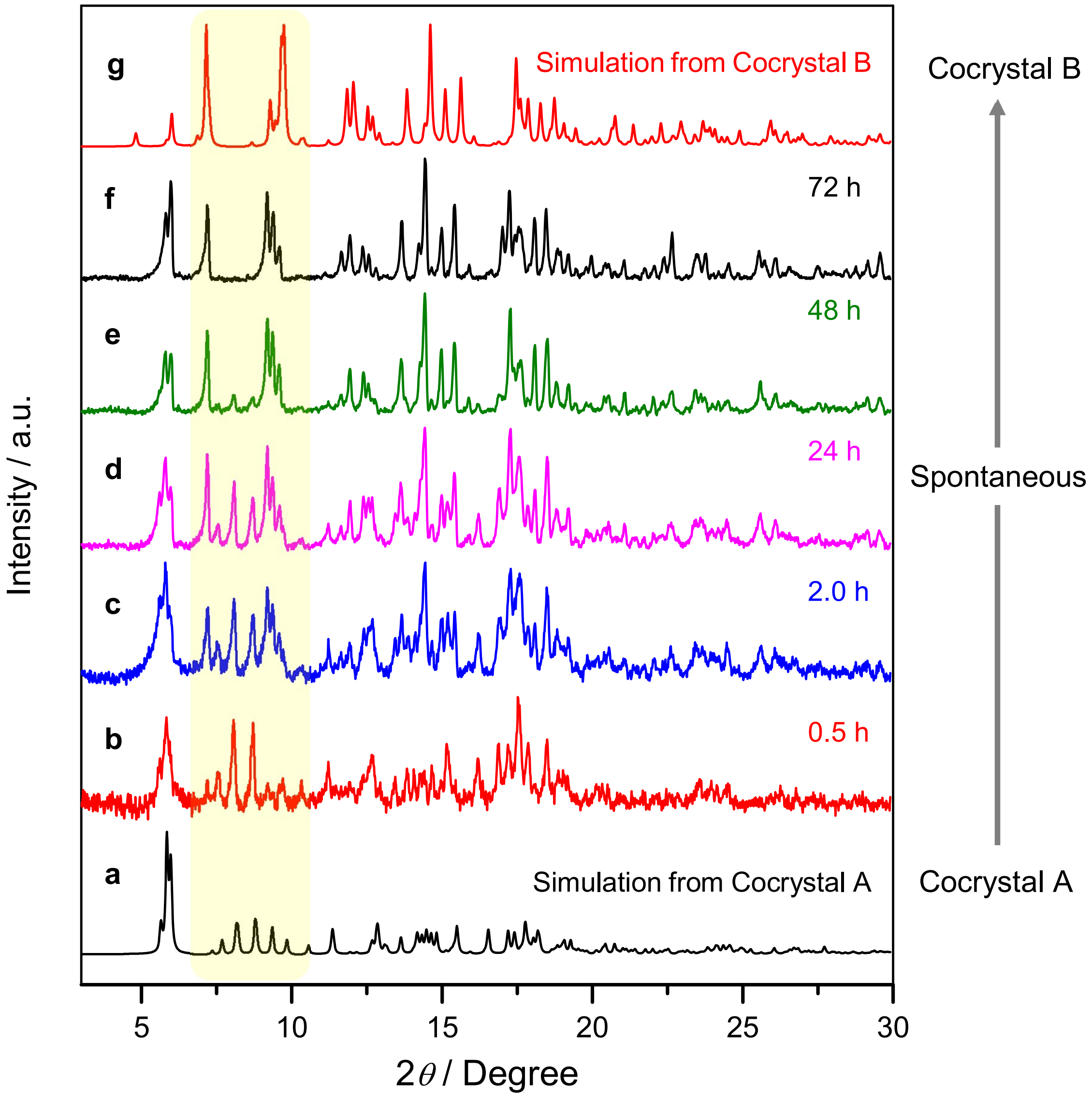

Supplementary Figure 23 | Powder X-ray diffraction patterns of microcrystals obtained by adding *i*Pr_2O to aqueous solutions of β-CD and $[AuBr_4]^-$ anion. (a) Simulation derived from the single-crystal X-ray crystallographic data for $HAuBr_4$•2(*i*Pr_2O)⊂2β-CD cocrystal, defined as cocrystal A**.** (b−f) The synthetic $HAuBr_4$•2(*i*Pr_2O)⊂2β-CD microcrystals after allowing them to stand in the mother liquid for 0.5, 2.0, 24, 48, and 72 h. (g) Simulation derived from the single-crystal X-ray crystallographic data for 0.5($HAuBr_4$)⊂2β-CD cocrystal, defined as cocrystal B. This phenomenon demonstrates the fact that the $HAuBr_4$•2(*i*Pr_2O)⊂2β-CD co-precipitate changes from cocrystal A to cocrystal B over time. The highlighted region with yellow shows the changes in characteristic peaks of cocrystals A and B over time.

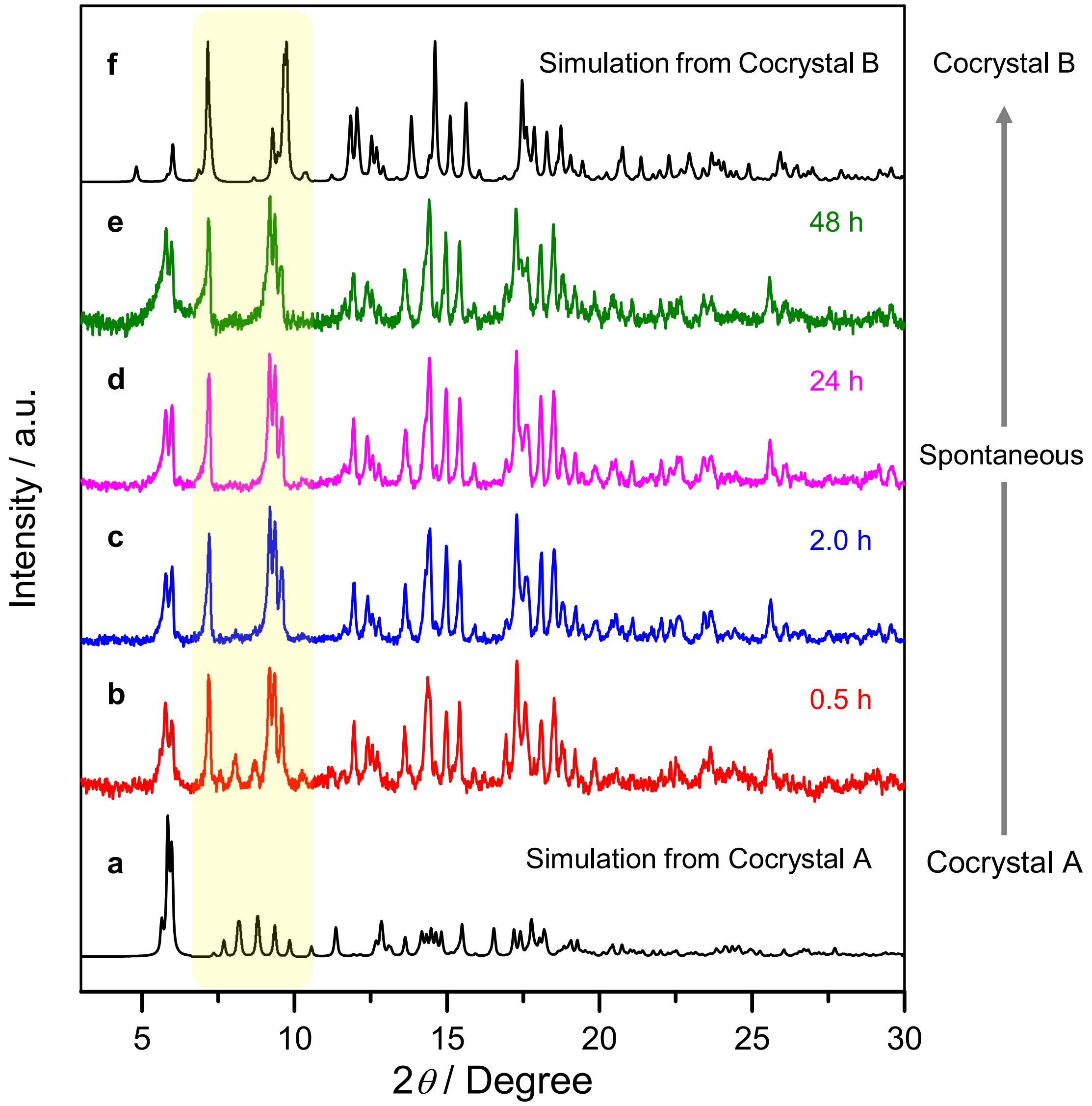

Supplementary Figure 24 | Powder X-ray diffraction patterns of microcrystals obtained by adding hexane to aqueous solutions of β-CD and $[AuBr_4]^-$ anion. (a) Simulation derived from the single-crystal X-ray crystallographic data for cocrystal A. (b–e) The synthetic microcrystals obtained by adding hexane to the solution of $[AuBr_4]^- \subset$β-CD after allowing them to stand in the mother liquid for 0.5, 2.0, 24 and 48 h. (f) Simulation derived from the single-crystal X-ray crystallographic data for cocrystal B. This phenomenon demonstrates the fact that the microcrystals change from cocrystal A to cocrystal B over time. The highlighted region with yellow shows the changes in characteristic peaks of cocrystals A and B over time.

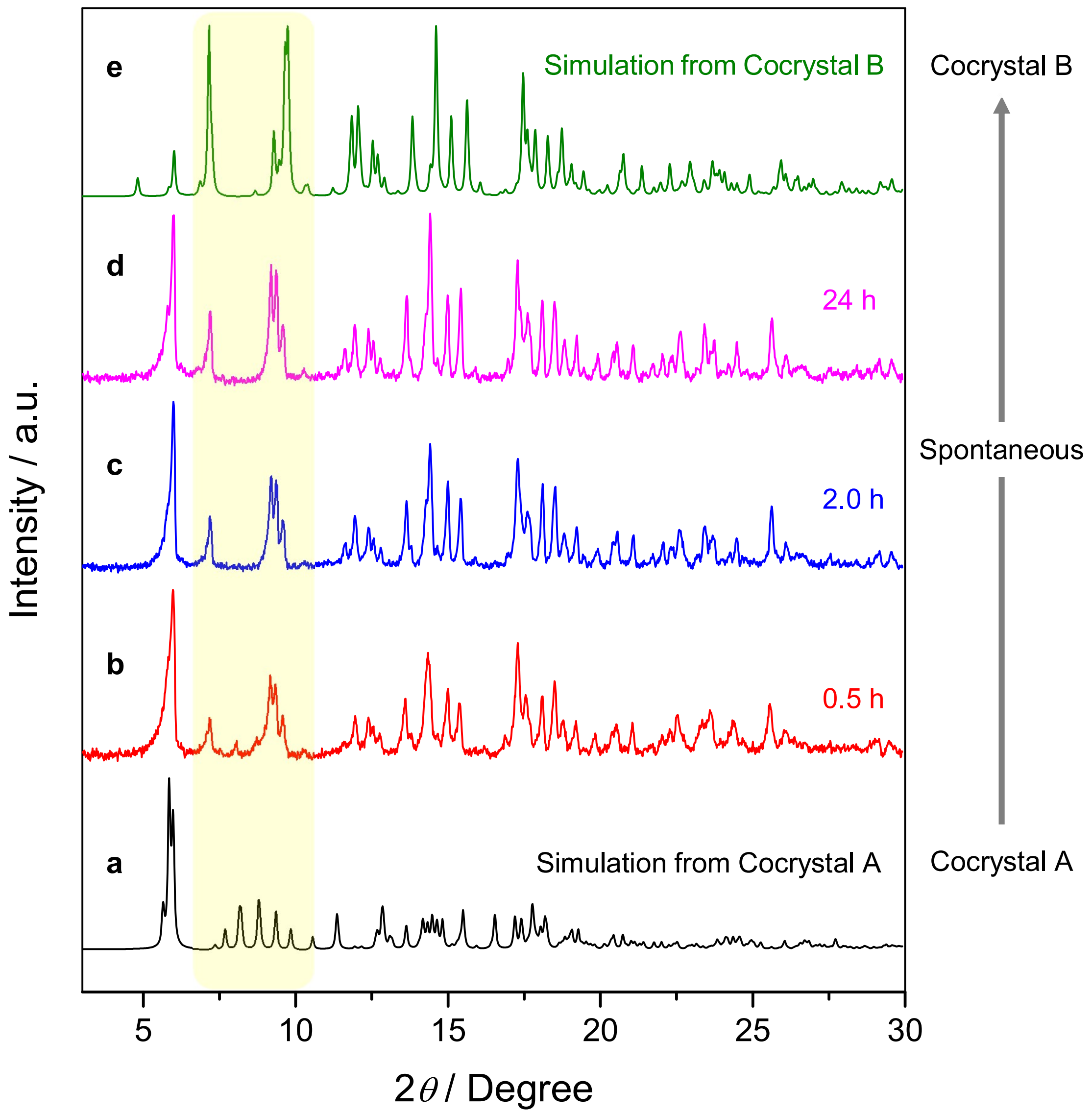

Supplementary Figure 25 | Powder X-ray diffraction patterns of microcrystals obtained by adding CH_2Cl_2 to aqueous solutions of β-CD and $[AuBr_4]^-$ anion. (a) Simulation derived from the single-crystal X-ray crystallographic data for cocrystal A. (b–d) The synthetic microcrystals obtained by adding CH_2Cl_2 to the solution of $[AuBr_4]^-\subset$β-CD after allowing them to stand in the mother liquid for 0.5, 2.0, and 24 h. (e) Simulation derived from the single-crystal X-ray crystallographic data for cocrystal B. This phenomenon demonstrates the fact that the microcrystals change from cocrystal A to cocrystal B over time. The highlighted region with yellow shows the changes in characteristic peaks of cocrystals A and B over time.

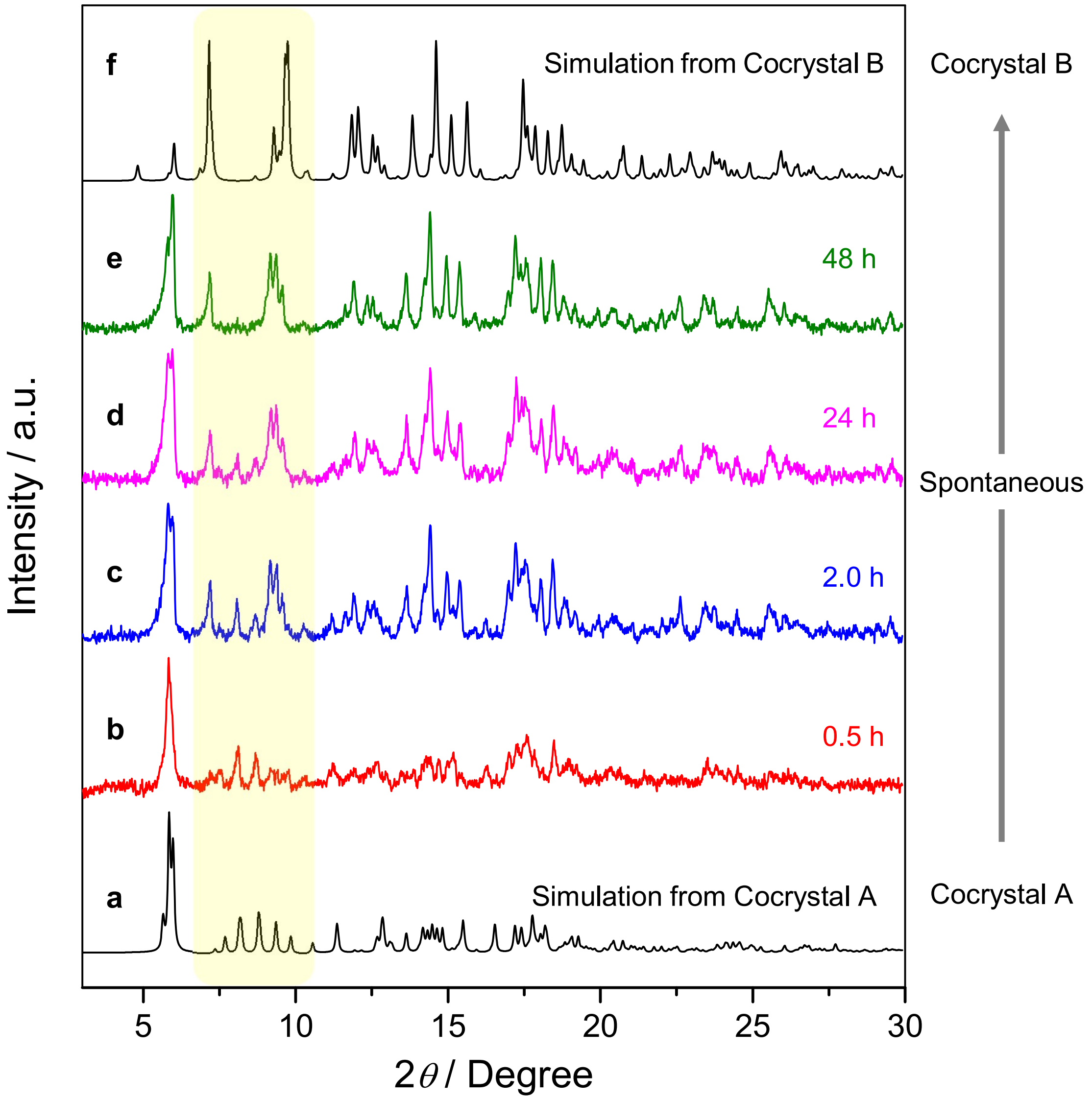

Supplementary Figure 26 | Powder X-ray diffraction patterns of microcrystals obtained by adding $CHCl_3$ to aqueous solutions of β-CD and $[AuBr_4]^-$ anion. (a) Simulation derived from the single-crystal X-ray crystallographic data for cocrystal A. (b–e) The synthetic microcrystals obtained by adding $CHCl_3$ to the solution of $[AuBr_4]^- \subset$β-CD after allowing them to stand in the mother liquid for 0.5, 2.0, 24, and 48 h. (f) Simulation derived from the single-crystal X-ray crystallographic data for cocrystal B. This phenomenon demonstrates the fact that the microcrystals change from cocrystal A to cocrystal B over time. The highlighted region with yellow shows the changes in characteristic peaks of cocrystals A and B over time.

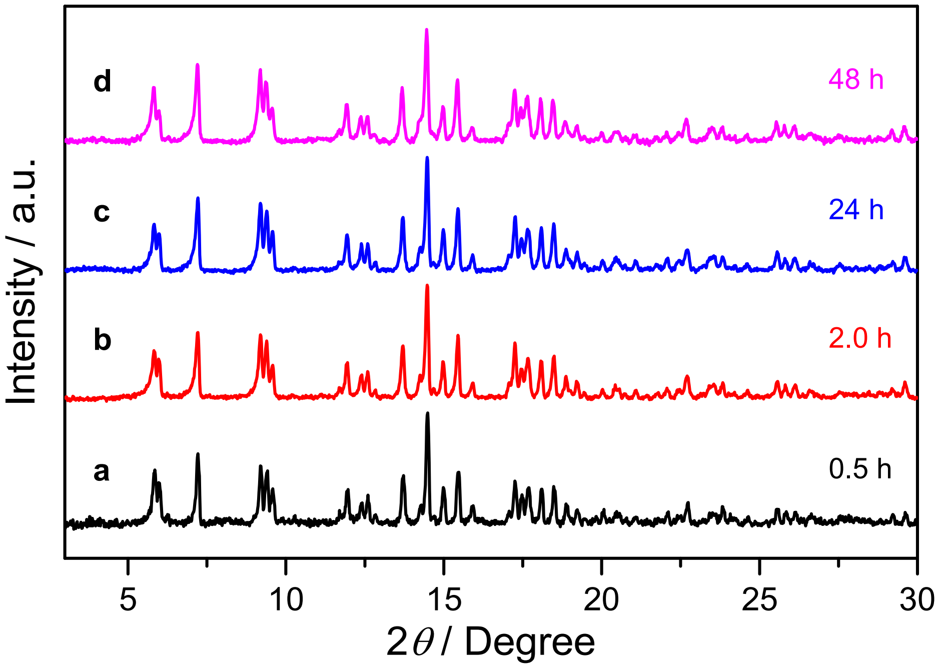

Supplementary Figure 27 | Powder X-ray diffraction patterns of the microcrystals were obtained by adding benzene to aqueous solutions of β-CD and $[AuBr_4]^-$ anion. (a–d) The synthetic microcrystals were obtained by adding benzene to the solution of $[AuBr_4]^-$⊂β-CD after allowing them to stand in the mother liquid for 0.5, 2.0, 24, and 48 h. This phenomenon demonstrates the fact that the microcrystals haven't undergone a cocrystal-to-cocrystal transformation.

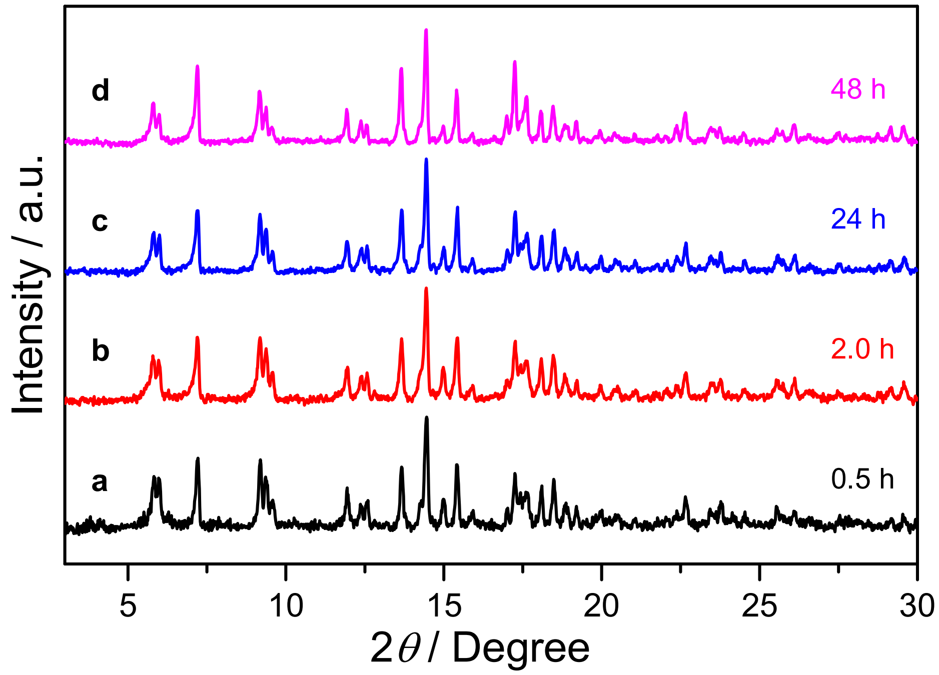

Supplementary Figure 28 | Powder X-ray diffraction patterns of microcrystals obtained by adding toluene to aqueous solutions of β-CD and $[AuBr_4]^-$ anion. (a–d) The synthetic cocrystals were obtained by adding toluene to the solution of $[AuBr_4]^-$⊂β-CD after allowing them to stand in the mother liquid for 0.5, 2.0, 24, and 48 h. This phenomenon demonstrates the fact that the microcrystals haven't undergone a cocrystal-to-cocrystal transformation.

Supplementary Note 6. X-Ray photoelectron spectroscopy

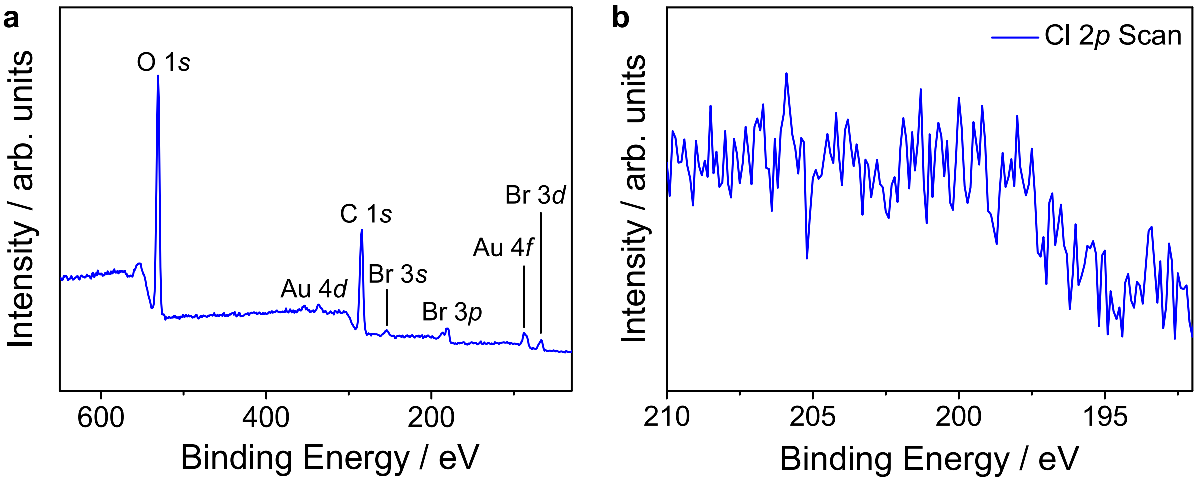

Supplementary Figure 29 | X-Ray photoelectron spectra of the microcrystals obtained by adding CH_2Cl_2 to aqueous solutions of β-CD and $[AuBr_4]^-$ anion, followed by filtration, washing with H_2O and air drying. (a) Full scan spectrum of the microcrystals, (b) narrow scan of Cl 2*p*.

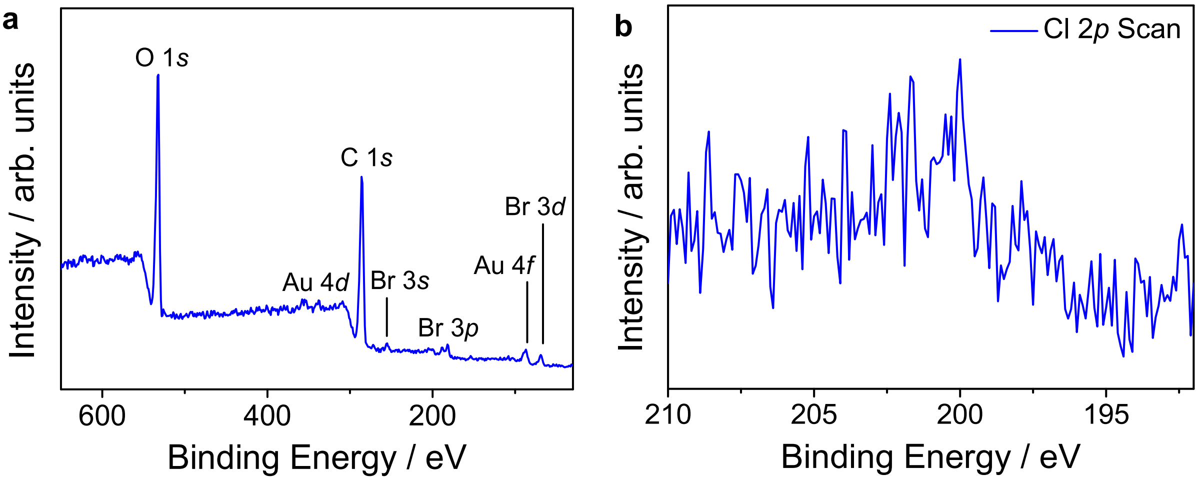

Supplementary Figure 30 | X-Ray photoelectron spectra of the microcrystals obtained by adding $CHCl_3$ to aqueous solutions of β-CD and $[AuBr_4]^-$ anion, followed by filtration, washing with H_2O and air drying. (a) Full scan spectrum of the microcrystals, (b) narrow scan of Cl 2*p*.

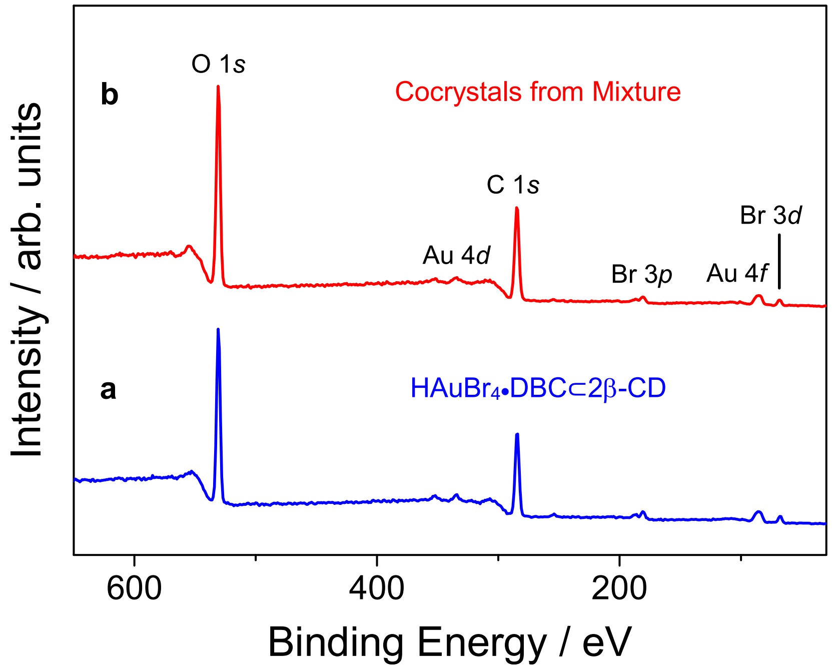

Supplementary Figure 31 | X-Ray photoelectron spectra of (a) the $HAuBr_4$•DBC⊂2β-CD microcrystals, and (b) the microcrystals obtained by adding DBC and β-CD to a leaching solution of electronic waste.

Supplementary Note 7. Fourier-transform infrared spectroscopy

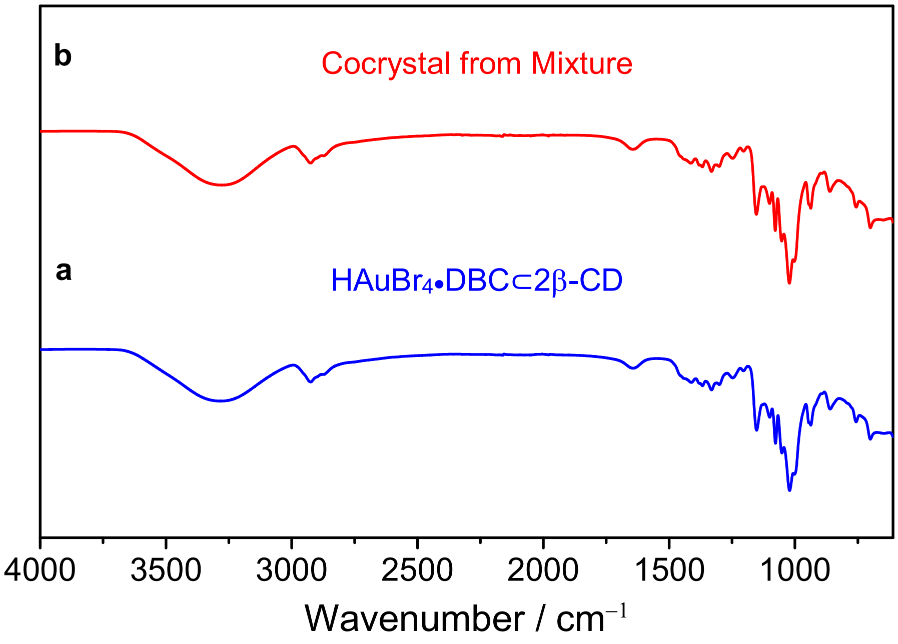

Supplementary Figure 32 | Fourier-transform infrared (FTIR) spectra of (a) $HAuBr_4$•DBC⊂2β-CD cocrystals, and (b) the cocrystals obtained by adding β-CD and DBC to a gold-bearing leaching solution of electronic waste.

Supplementary Note 8. ICP-MS Analysis

Quantification of gold (Au) was accomplished using ICP-MS on acidified samples. Specifically, samples (100 µL) designated for Au analysis were digested in 10 mL 2.0% HNO_3 and 2.0% HCl (v/v) aqueous solution. A quantitative standard was made using a 100 µg/mL Au elemental standard (Inorganic Ventures, Christiansburg, VA, USA), which was used to create a 100 ng/g Au standard in 2.0% HNO_3 and 2.0% HCl (v/v) in a total sample volume of 50 mL. A solution of 2.0% HNO_3 and 2.0% HCl (v/v) was used as the calibration blank. All the gold-recovery experiments were independently duplicated. Supplementary Tables 6 and 7 show one set of data. The average gold-recovery efficiencies with standard deviations are presented in Fig. 5g and Supplementary Fig. 33.

Supplementary Table 6 | Effect of changes in concentration of $[AuBr_4]^-$ on the efficiency of recovering gold from mixture solution

Concentration of $[AuBr_4]^-$ / mM	Avg. Au / ppb	Au in Filtrate / µg	Total Au / µg	Au-Recovery Efficiency
0.005	34.901	0.873	0.928	0.060
0.01	36.380	0.910	1.856	0.510
0.025	22.983	0.575	4.640	0.876
0.05	24.123	0.603	9.280	0.935
0.075	30.130	0.753	13.920	0.946
0.1	42.046	1.051	18.559	0.943
0.25	43.627	1.091	46.399	0.976
0.5	23.450	0.586	92.797	0.994
0.75	36.907	0.923	139.196	0.993
1	48.028	1.201	185.594	0.994

Supplementary Table 7 | Changes in the efficiency of recovering gold from mixture solution with respect to adding different additives in real time

Different Additives	Avg. Au / ppb	Au in Filtrate / μg	Total Au / μg	Au-Recovery Efficiency
Hexane	398.941	7.979	774.014	0.990
Benzene	130.093	2.602	774.014	0.997
Chloroform	117.617	2.352	774.014	0.997
Dichloromethane	509.513	10.190	774.014	0.987
Toluene	22.016	0.440	774.014	0.999
Isopropyl Ether	188.471	3.769	774.014	0.995
Dibutyl Carbitol	59.700	1.194	774.014	0.998

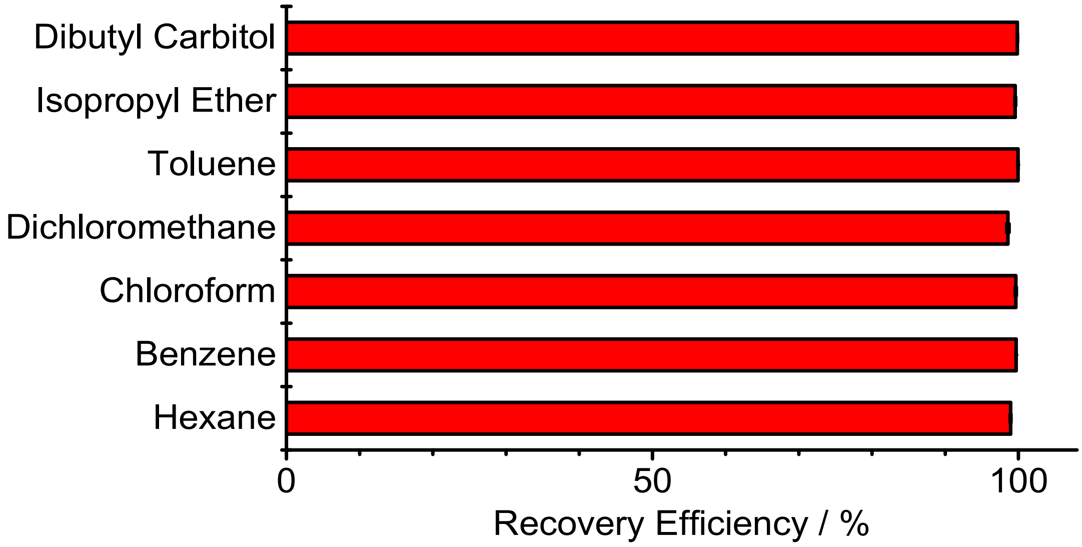

Supplementary Figure 33 | Changes in the efficiency of recovering gold from mixture solution with respect to adding different additives. After adding β-CD and 0.1% (v/v) of different additives to leaching solutions of gold-bearing electronic scrap, co-precipitates were formed and filtered within 1 min, and the gold-recovery efficiencies were determined by ICP-MS. These results indicate that all the additives can recover ~99% gold from mixture solutions when co-precipitates were separated before the cocrystal-to-cocrystal transformation. The error bar represents the standard deviation of two independent measurements.

Supplementary Note 9. Scanning electron microscopy

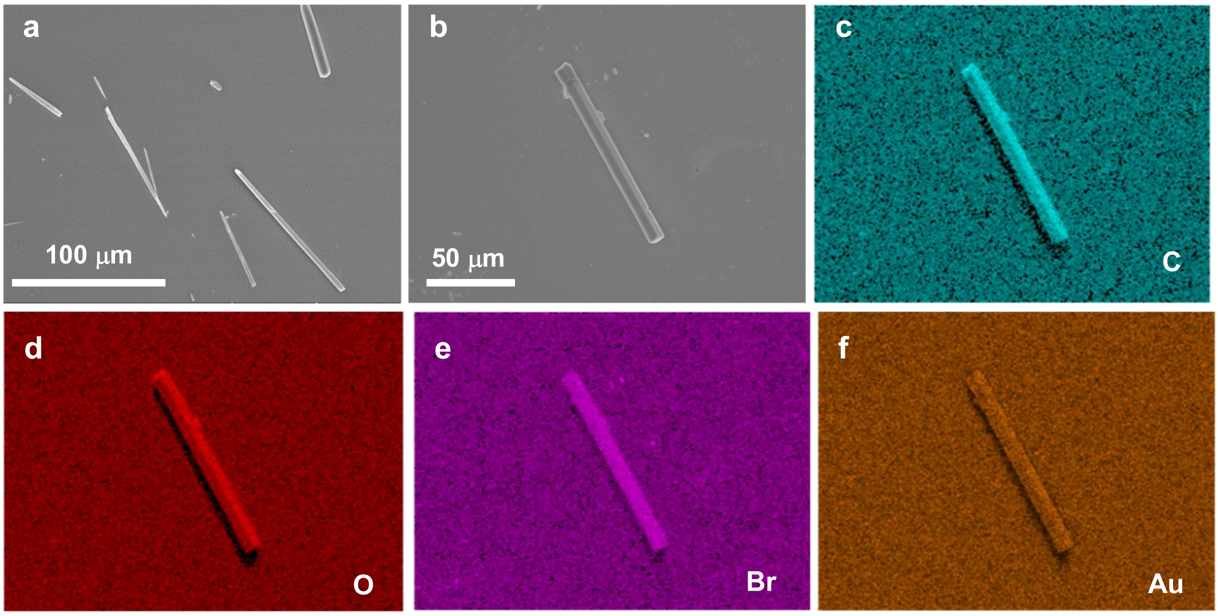

Supplementary Figure 34 | (a) SEM images of the $HAuBr_4$•DBC⊂2β-CD microcrystals obtained by adding DBC to an aqueous solution of $[AuBr_4]^-$⊂β-CD complex. (b–f) SEM-EDS Elemental maps of the $HAuBr_4$•DBC⊂2β-CD microcrystals, showing all the component elements well distributed in the microrods.

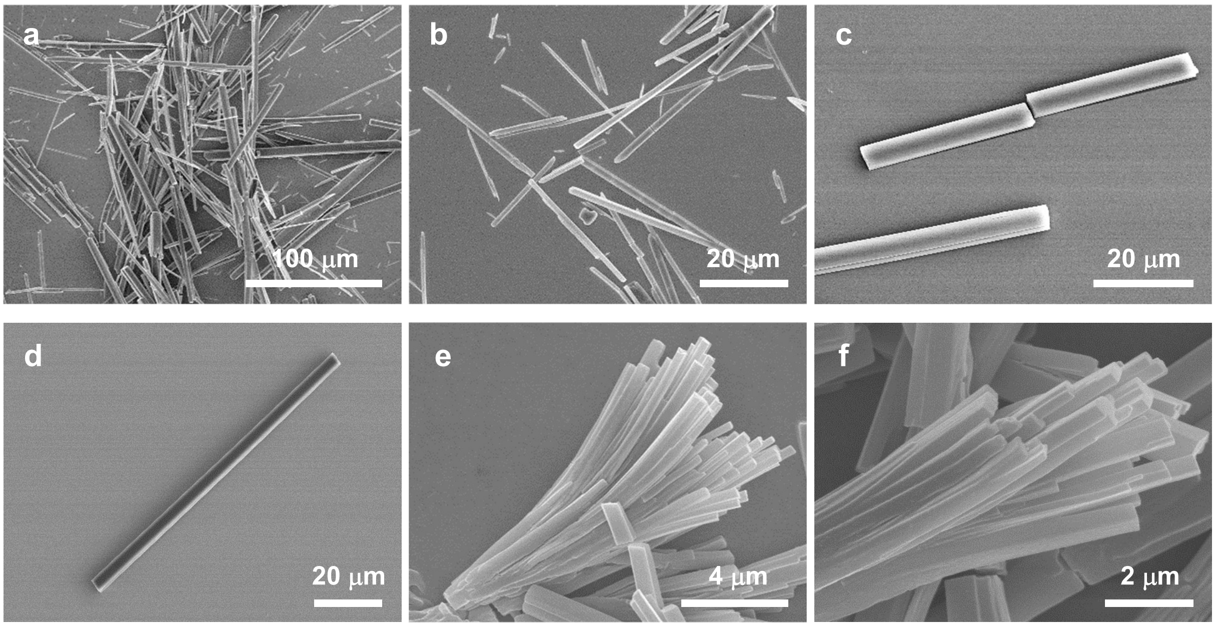

Supplementary Figure 35 | SEM images of the $HAuBr_4$•DBC⊂2β-CD microcrystals obtained by adding β-CD and DBC to a leaching solution of electronic waste, illustrating (a–d) the rod-like microstructures, and (e, f) some large crystals are made up of nanorods.

Supplementary Note 10. Theoretical calculations

(1) Visualization of noncovalent interaction

Independent gradient model (IGM) analysis is an approach[6] based on promolecular density—an electron density model prior to molecule formation—to identify and isolate intermolecular interactions. Strong polar attractions and Van der Waals contacts are visualized as an iso-surface with blue and green colors, respectively. Single-crystal superstructures were used as input files. The binding surface was calculated using the Multiwfn 3.6 program[7] through function 20 (visual study of weak interaction) and visualized by Chimera software[8].

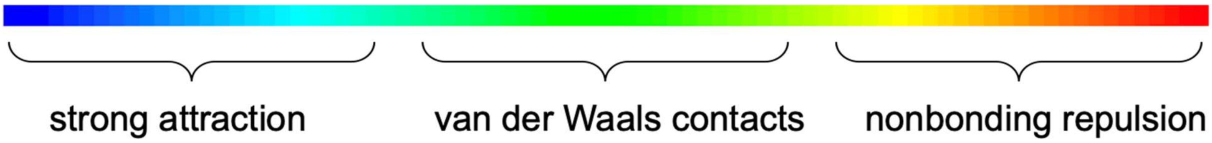

Supplementary Figure 36 | Color-coded sign $(\lambda_2)_\rho$ scale bar

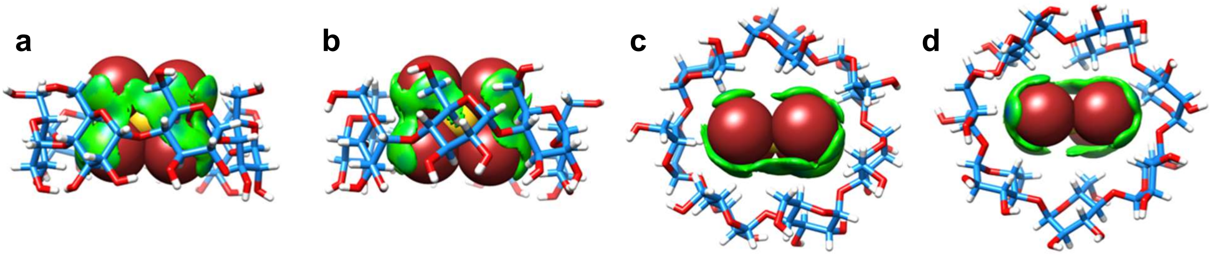

Supplementary Figure 37 | (a, b) Side-on and (c, d) top-down views of tubular (β-CD) and space-filling ($[AuBr_4]^-$) representations of $[AuBr_4]^-\subset\beta$-CD, showing that intermolecular binding iso-surface in $KAuBr_4\subset\beta$-CD complex. Δκ inter (ρ) = 0.003 a.u. Iso-surfaces are colored according to a BGR scheme over the range $-0.05 < \mathrm{sign}(\lambda_2)\rho < +0.05$ a.u. H white, C skyblue, O red, Br brown, and Au yellow.

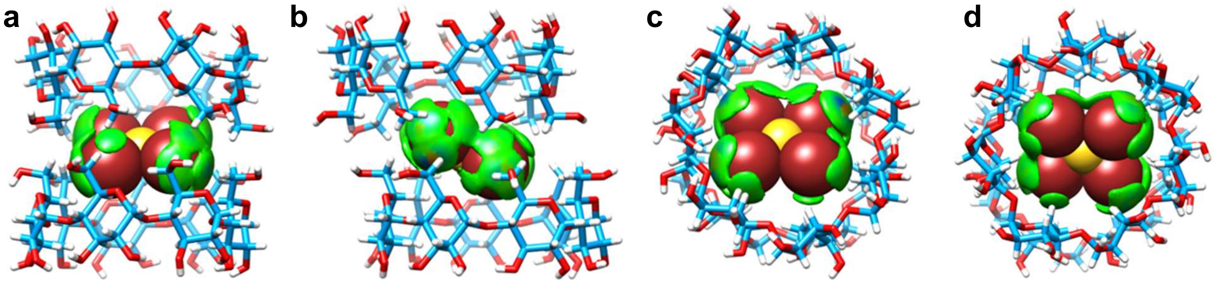

Supplementary Figure 38 | (a, b) Side-on and (c, d) top-down views of tubular (β-CD) and space-filling ($[AuBr_4]^-$) representations of $[AuBr_4]^-\subset2\beta$-CD, showing that intermolecular binding iso-surface in $HAuBr_4$•DBC⊂2β-CD cocrystal. Δκ inter (ρ) = 0.003 a.u. Iso-surfaces are colored according to a BGR scheme over the range $-0.05 < \mathrm{sign}(\lambda_2)\rho < +0.05$ a.u. H white, C skyblue, O red, Br brown, and Au yellow.

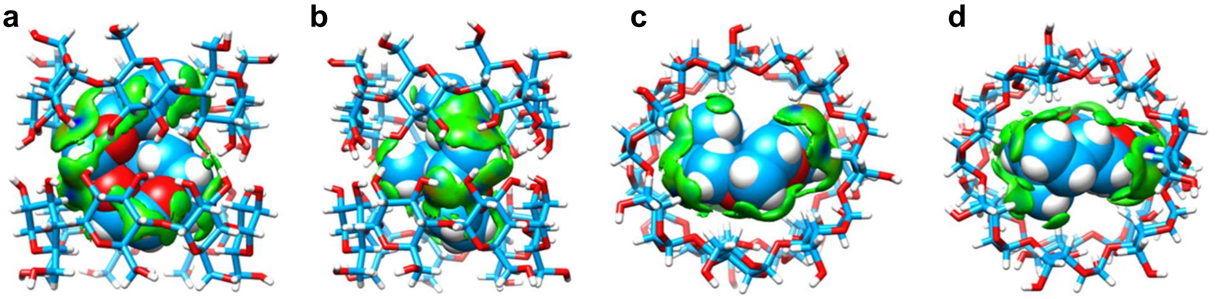

Supplementary Figure 39 | (a, b) Side-on and (c, d) top-down views of tubular (β-CD) and space-filling (DBC) representations of DBC⊂2β-CD, showing that intermolecular binding iso-surface in $HAuBr_4$•DBC⊂2β-CD cocrystal. Δκ inter (ρ) = 0.003 a.u. Iso-surfaces are colored according to a BGR scheme over the range –0.05 < sign(λ_2)ρ < +0.05 a.u. H white, C skyblue, and O red.

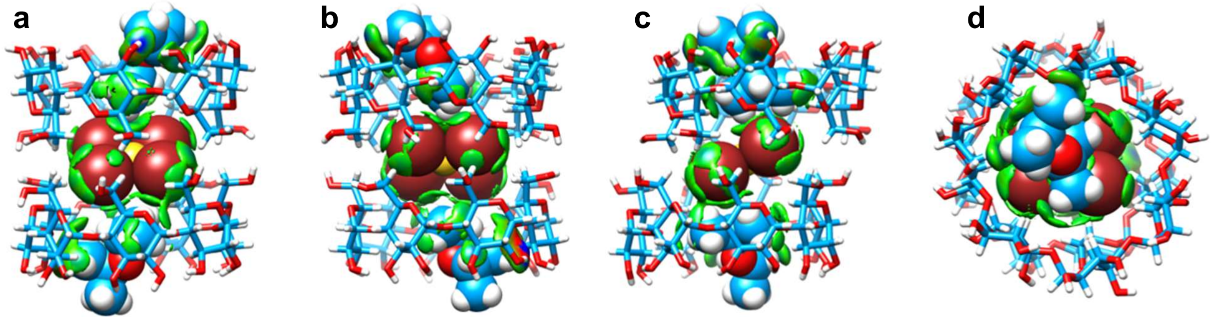

Supplementary Figure 40 | (a–c) Side-on and (d) top-down views of tubular (β-CD) and space-filling ($[AuBr_4]^-$ and $i$$Pr_2O$) representations of $HAuBr_4$•2($i$$Pr_2O$)⊂2β-CD cocrystal, showing that intermolecular binding iso-surface. Δκ inter (ρ) = 0.003 a.u. Iso-surfaces are colored according to a BGR scheme over the range –0.05 < sign(λ_2)ρ < +0.05 a.u. H white, C skyblue, O red, Br brown, and Au yellow.

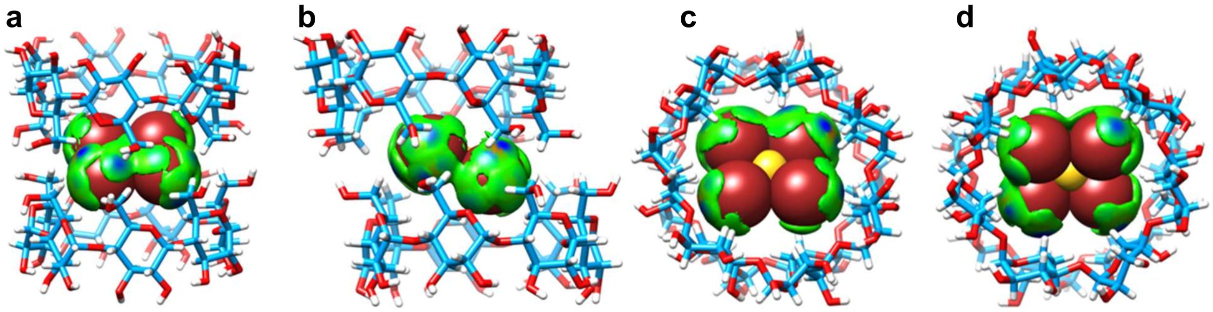

Supplementary Figure 41 | (a, b) Side-on and (c, d) top-down views of tubular (β-CD) and space-filling ($[AuBr_4]^-$) representations of $[AuBr_4]^-$⊂2β-CD, showing that intermolecular binding iso-surface in $HAuBr_4$•2($i$$Pr_2O$)⊂2β-CD cocrystal. Δκ inter (ρ) = 0.003 a.u. Iso-surfaces are colored according to a BGR scheme over the range –0.05 < sign(λ_2)ρ < +0.05 a.u. H white, C skyblue, O red, Br brown, and Au yellow.

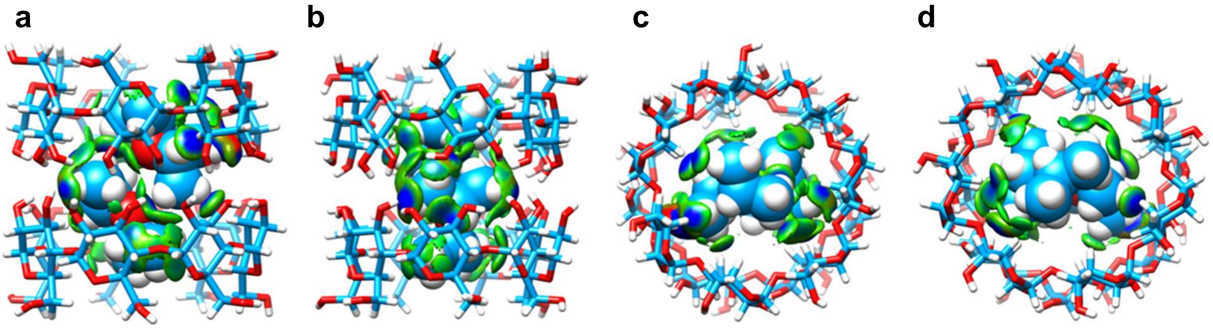

Supplementary Figure 42 | (a, b) Side-on and (c, d) top-down views of tubular (β-CD) and space-filling (two iPr$_2$O) representations of 2(iPr$_2$O)⊂2β-CD, showing that intermolecular binding iso-surface in HAuBr$_4$•2(iPr$_2$O)⊂2β-CD cocrystal. Δκ inter (ρ) = 0.003 a.u. Iso-surfaces are colored according to a BGR scheme over the range –0.05 < sign(λ$_2$)ρ < +0.05 a.u. H white, C skyblue, and O red.

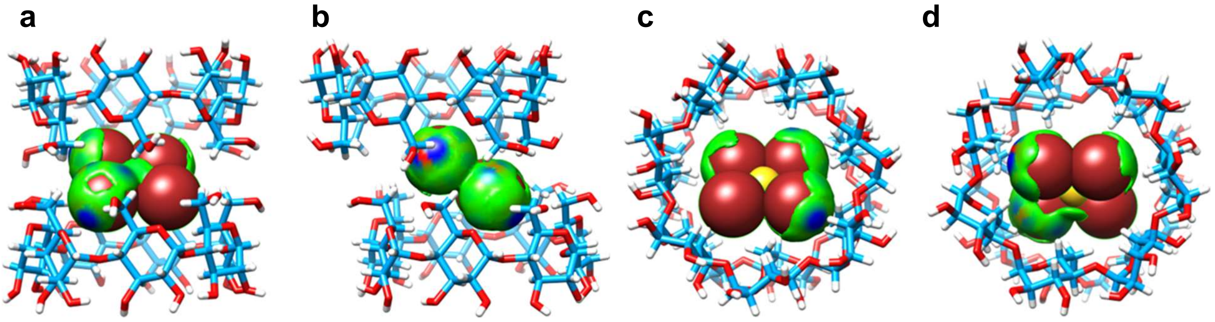

Supplementary Figure 43 | (a, b) Side-on and (c, d) top-down views of tubular (β-CD) and space-filling ([AuBr$_4$]$^-$) representations of [AuBr$_4$]$^-$⊂2β-CD, showing that intermolecular binding iso-surface in 0.5(HAuBr$_4$)⊂2β-CD cocrystal. Δκ inter (ρ) = 0.003 a.u. Iso-surfaces are colored according to a BGR scheme over the range –0.05 < sign(λ$_2$)ρ < +0.05 a.u. H white, C skyblue, O red, Br brown, and Au yellow.

(2) Binding energy

The superstructures from the single-crystal X-ray diffraction were used for the density functional theory (DFT) calculations in the Orca program[9] (version 4.1.2) using the hybrid generalized gradient approximation (GGA) Becke three-parameter Lee-Yang-Parr[10] (B3LYP) functional, the Ahlrich's double zeta basis set with a polarization function[11] Def2-SVP, and Grimme's third generation atom-pairwise dispersion correction with Becke Johnson damping[12] (D3BJ); an integration grid of four was used throughout. To further speed up the DFT optimizations, the Coulomb integral and numerical chain-of-sphere integration for the HF exchange[13,14] (RIJCOSX) method was applied with the Def2/J auxiliary basis[15]. All calculations were single points and ran in vacuum. The binding energies were computed as ΔE_b = products – reactants, where products are the complexes and the reactants are the individual molecules and ions.

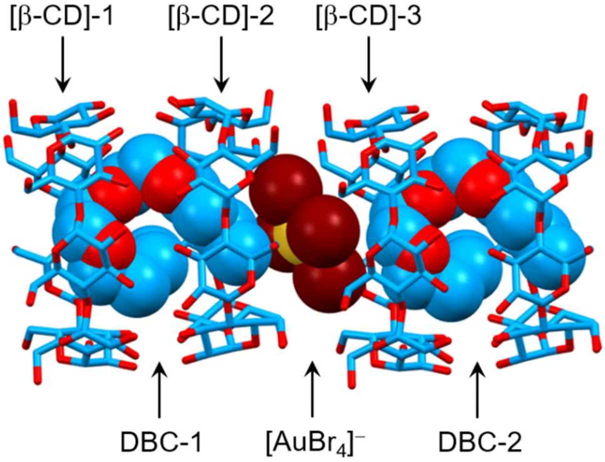

Supplementary Figure 44 | Tubular and space-filling representation, showing the nearest neighbors around a central $[AuBr_4]^-$ anion in the crystal superstructure of $HAuBr_4$•DBC⊂2β-CD. Each β-CD, $[AuBr_4]^-$ and DBC is given a label, and the binding energies between the selected species are presented in Supplementary Table 8.

Supplementary Table 8 | Results of DFT calculation for the binding energies in $HAuBr_4$•DBC⊂2β-CD cocrystal

Entry	Electronic Energy E_{DFT} / Hartree	Binding Energy ΔE_b / kcal mol^{-1}
$[AuBr_4]^-$···[β-CD]-2	–14700.19637	–32.44
$[AuBr_4]^-$···[β-CD]-3	–14700.20786	–39.64
[β-CD]-2···$[AuBr_4]^-$···[β-CD]-3	–18969.56797	–93.03
$[AuBr_4]^-$···DBC-1	–11128.27065	–8.32
$[AuBr_4]^-$···DBC-2	–11128.27038	–8.15
DBC-1···$[AuBr_4]^-$···DBC-2	–11825.67981	–16.10
DBC-1···[β-CD]-1	–4966.737547	–35.61
DBC-1···[β-CD]-2	–4966.720184	–30.37
[β-CD]-1···DBC-1···[β-CD]-2	–9236.132379	–110.79
[β-CD]-2···[β-CD]-3 (Primary faces)	–8538.596235	–23.32
[β-CD]-1···[β-CD]-2 (Secondary faces)	–8538.634395	–47.27
$[AuBr_4]^-$···[β-CD]-1···DBC-1	–15397.60983	–42.92
$[AuBr_4]^-$···[β-CD]-2···DBC-1	–15397.65162	–74.80
$[AuBr_4]^-$···[β-CD]-2···[β-CD]-3···DBC-1	–19667.0234	–129.85
$[AuBr_4]^-$···[β-CD]-2···[β-CD]-3···DBC-1···DBC-2	–20364.48552	–170.87

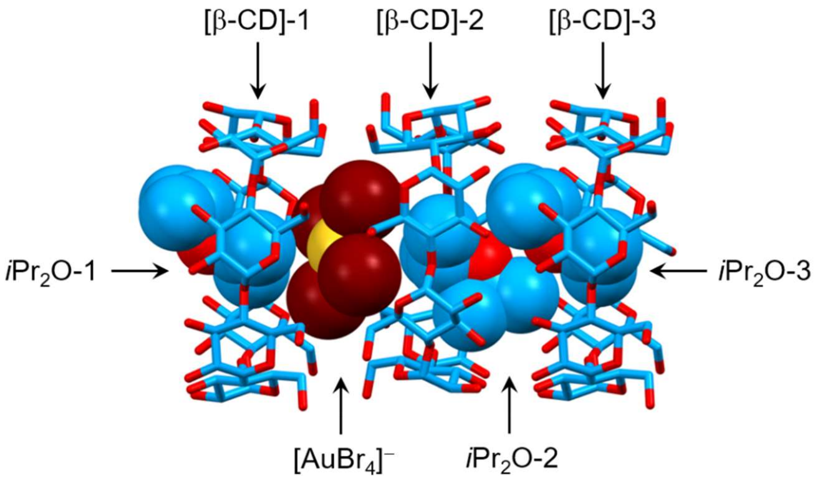

Supplementary Figure 45 | Tubular and space-filling representation showing the nearest neighbors around a $[AuBr_4]^-$ anion in the crystal superstructure of $HAuBr_4$•2($i$$Pr_2O$)⊂2β-CD. Each β-CD, $[AuBr_4]^-$ and $i$$Pr_2O$ are given a label, and the binding energies between the selected species are presented in Supplementary Table 9.

Supplementary Table 9 | Results of DFT calculation for the binding energies in $HAuBr_4$•2($i$$Pr_2O$)⊂2β-CD cocrystal

Entry	Electronic Energy E_{DFT} / Hartree	Binding Energy ΔE_b / kcal mol^{-1}
$[AuBr_4]^-$···[β-CD]-1	–14700.15893	–32.47
$[AuBr_4]^-$···[β-CD]-2	–14700.24900	–32.74
[β-CD]-1···$[AuBr_4]^-$···[β-CD]-2	–18969.58363	–84.15
$[AuBr_4]^-$···$i$$Pr_2O$-1	–10742.54258	–3.96
$[AuBr_4]^-$···$i$$Pr_2O$-2	–10742.52625	–7.18
$i$$Pr_2O$-1···$[AuBr_4]^-$···$i$$Pr_2O$-2	–11054.21382	–10.82
$i$$Pr_2O$-1···[β-CD]-1	–4580.967062	–20.45
[β-CD]-1···[β-CD]-2 (Primary faces)	–8538.63553	–25.42
[β-CD]-2···[β-CD]-3 (Secondary faces)	–8538.656367	–38.49
$[AuBr_4]^-$···[β-CD]-1···$i$$Pr_2O$-1	–15011.87652	–54.94

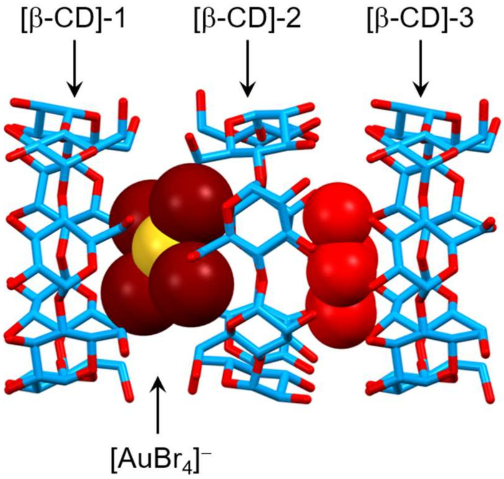

Supplementary Figure 46 | Tubular and space-filling representation showing the nearest neighbors around a $[AuBr_4]^-$ anion in the crystal superstructure of 0.5 ($HAuBr_4$)⊂2β-CD. Each β-CD and $[AuBr_4]^-$ is given a label, and the binding energies between the selected species are presented in Supplementary Table 10. The red balls represent the O atom in H_2O molecules, which are located in the lattice space between the secondary faces of the two β-CD tori.

Supplementary Table 10 | Results of DFT calculation for the binding energies in 0.5($HAuBr_4$)⊂2β-CD cocrystal

Entry	Electronic Energy E_{DFT} / Hartree	Binding Energy ΔE_b / kcal mol^{-1}
$[AuBr_4]^-$···[β-CD]-1	–14700.16752	–35.97
$[AuBr_4]^-$···[β-CD]-2	–14700.16005	–29.21
[β-CD]-1···$[AuBr_4]^-$···[β-CD]-2	–18969.54003	–82.46
[β-CD]-1···[β-CD]-2 (Primary faces)	–8538.630479	–23.17
[β-CD]-2···[β-CD]-3 (Secondary faces)	–8538.638149	–27.98

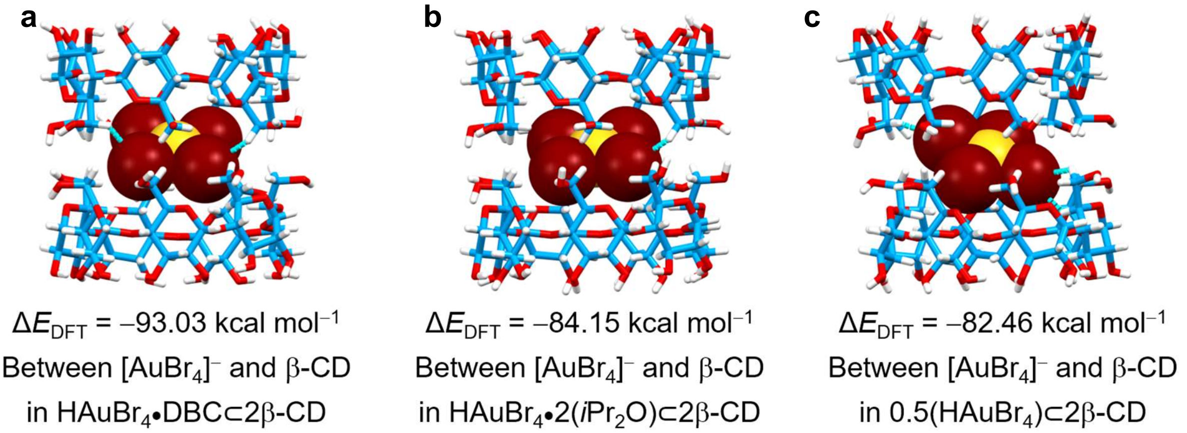

Supplementary Figure 47 | (a–c) The DFT calculated binding energy between β-CD and $[AuBr_4]^-$ in the three cocrystals.

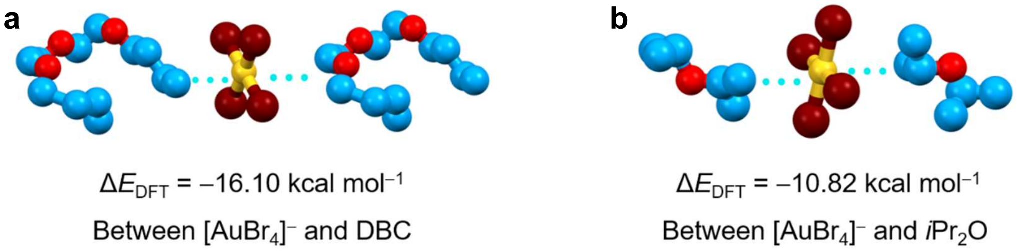

Supplementary Figure 48 | The DFT calculated binding energy between (a) $[AuBr_4]^-$ and DBC in $HAuBr_4$•DBC⊂2β-CD cocrystal, (b) $[AuBr_4]^-$ and *i*Pr_2O in $HAuBr_4$•2(*i*Pr_2O)⊂2β-CD cocrystal.

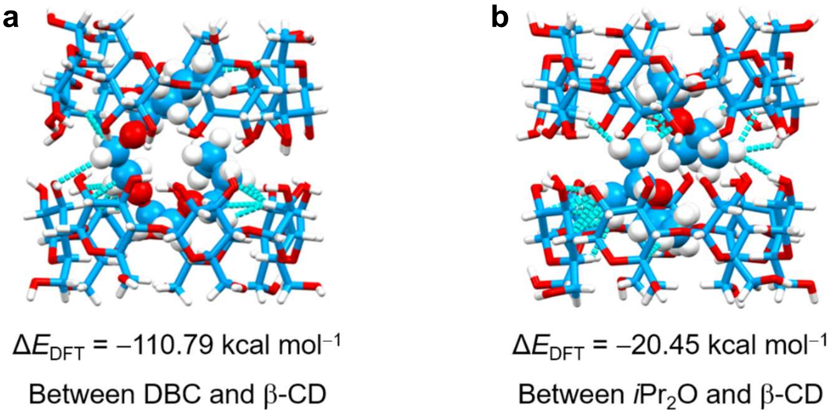

Supplementary Figure 49 | The DFT calculated binding energy between (a) DBC and β-CD in $HAuBr_4$•DBC⊂2β-CD cocrystal, (b) *i*Pr_2O and β-CD in $HAuBr_4$•2(*i*Pr_2O)⊂2β-CD cocrystal.

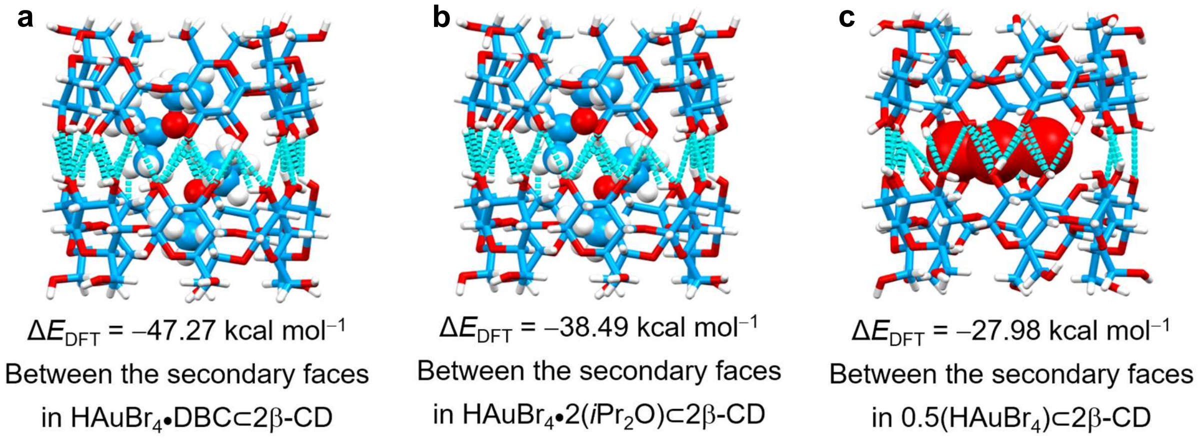

Supplementary Figure 50 | (a–c) The DFT calculated binding energy between the secondary faces of the two neighboring β-CD tori in the three cocrystals. The red balls represent the O atom in H_2O molecules, which are located in the lattice space between the secondary faces of the two β-CD tori.

Supplementary Note 11. Thermogravimetric analysis

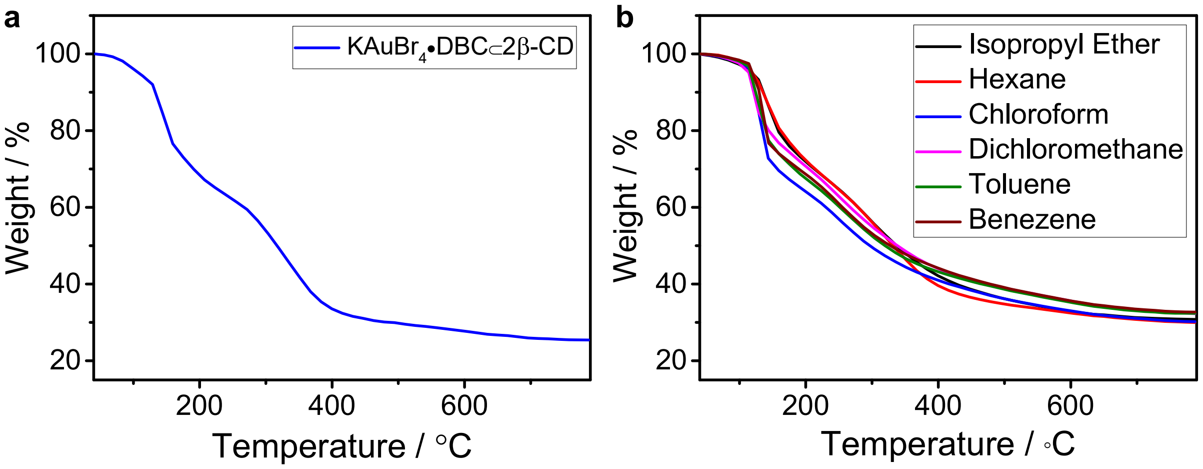

Supplementary Figure 51 | Thermogravimetric analysis (TGA) of (a) $KAuBr_4$•DBC⊂2β-CD and (b) the co-precipitates obtained by adding different additives to the aqueous solutions of β-CD and $[AuBr_4]^-$ anion

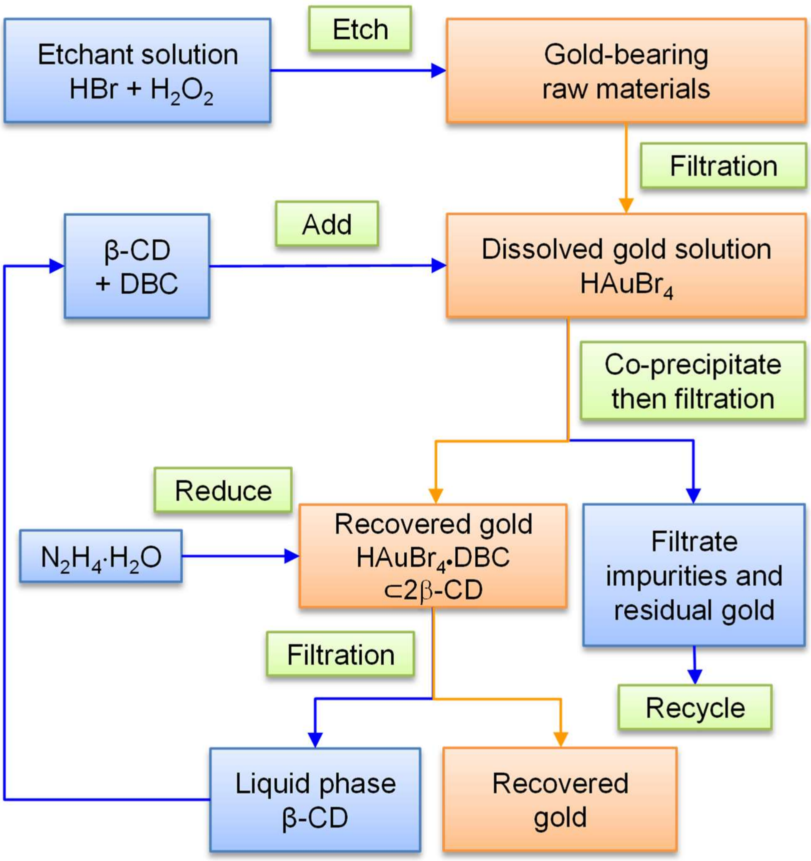

Supplementary Figure 52 | Gold-recovery flow diagram, which is proposed based on the additive-induced supramolecular polymerization of β-CD and $[AuBr_4]^-$ anions. Light orange arrows and boxes indicate the flow direction for the recovery of gold.

Supplementary References

1. Dolomanov, O. V., *et al.* OLEX2: A complete structure solution, refinement and analysis program. *J. Appl. Cryst.* **42**, 339−341 (2009).

2. Sheldrick, G. M. SHELXT—Integrated space-group and crystal-structure determination. *Acta. Cryst.* **A71**, 3−8 (2015).

3. Sheldrick, G. M. A short history of SHELX. *Acta. Cryst.* **A64**, 112−122 (2008).

4. Thorn, A., Dittrich, B., Sheldrick, G. M. Enhanced rigid-bond restraints. *Acta. Cryst.* **A68**, 448–451 (2012)

5. Thordarson, P. Determining association constants from titration experiments in supramolecular chemistry. *Chem. Soc. Rev.* **40**, 1305–1323 (2011)

6. Lefebvre, C., *et al.* Accurately extracting the signature of intermolecular interactions present in the NCl plot of the reduced density gradient versus electron density. *Phys. Chem. Chem. Phys.* **19**, 17928–17936 (2017).

7. Lu, T., Chen, F. Multiwfn: A multifunctional wavefunction analyzer. *J. Comput. Chem.* **33**, 580–592 (2012).

8. Pettersen, E. F., *et al.* UCSF Chimera−A visualization system for exploratory research and analysis. *J. Comput. Chem.* **25**, 1605–1612 (2004).

9. Neese, F. The ORCA program system. *Wiley Interdiscip. Rev.: Comput. Mol. Sci.* **2**, 73−78 (2012).

10. Becke, A. D. Density functional thermochemistry III. The Role of exact exchange. *J. Chem. Phys.* **98**, 5648−5652 (1993).

11. Weigend, F., Ahlrichs, R. Balanced basis sets of split valence, triple zeta valence and quadruple zeta valence quality for H to Rn: Design and assessment of accuracy. *Phys. Chem. Chem. Phys.* **7**, 3297−3305 (2005).

12. Grimme, S., Ehrlich, S., Goerigk, L. Effect of the damping function in dispersion corrected density functional theory. *J. Comp. Chem.* **32**, 1456−1465 (2011).

13. Neese, F. An improvement of the resolution of the identity approximation for the calculation of the Coulomb matrix. *J. Comp. Chem.* **24**, 1740−1747 (2003).

14. Izsák, R.; Neese, F. An overlap fitted chain of spheres exchange method. *J. Chem. Phys.* **135**, 144105 (2011).

15. Weigend, F. Accurate Coulomb-fitting basis sets for H to Rn. *Phys. Chem. Chem. Phys.* **8**, 1057−1065 (2006).
