# Supplementary material for: High-efficiency gold recovery by additive-induced supramolecular polymerization of β-cyclodextrin
Source: Nat Commun. 2023 Mar 9;14:1284. doi: 10.1038/s41467-023-36591-0 (PMC9998620; doi:10.1038/s41467-023-36591-0)
Supplement: Supplementary file 3 — Description of Additional Supplementary Items [file 41467_2023_36591_MOESM3_ESM.pdf]

Description of Additional Supplementary Items

Title: Supplementary Movie 1
Description: Additives-Induced Cocrystallization of $KAuBr_4$•DBC⊂2β-CD

Title: Supplementary Data 1
Description: Checkcif File for $KAuBr_4$⊂β-CD Cocrystal

Title: Supplementary Data 2
Description: Checkcif File for $HAuBr_4$•DBC⊂2β-CD Cocrystal

Title: Supplementary Data 3
Description: Checkcif File for $HAuBr_4$•2(*i*Pr_2O)⊂2β-CD Cocrystal (Cocrystal A)

Title: Supplementary Data 4
Description: Checkcif File for 0.5($HAuBr_4$)⊂2β-CD Cocrystal (Cocrystal B)
