# Supplementary material for: High-efficiency gold recovery by additive-induced supramolecular polymerization of β-cyclodextrin
Source: Nat Commun. 2023 Mar 9;14:1284. doi: 10.1038/s41467-023-36591-0 (PMC9998620; doi:10.1038/s41467-023-36591-0)

checkCIF/PLATON report

Structure factors have been supplied for datablock(s) cx2175

THIS REPORT IS FOR GUIDANCE ONLY. IF USED AS PART OF A REVIEW PROCEDURE FOR PUBLICATION, IT SHOULD NOT REPLACE THE EXPERTISE OF AN EXPERIENCED CRYSTALLOGRAPHIC REFEREE.

No syntax errors found. CIF dictionary Interpreting this report

Datablock: cx2175

Bond precision:	C-C = 0.0082 A		Wavelength=1.54184
Cell:	a=14.9054(1)	b=15.4374(1)	c=14.9191(1)
	alpha=90	beta=119.310(1)	gamma=90
Temperature:	100 K		

	Calculated	Reported
Volume	2993.43(5)	2993.43(4)
Space group	P 21	P 1 21 1
Hall group	P 2yb	P 2yb
Moiety formula	C42 H74 K O37, Au Br4, 2(H2 O)	C42 H74 K O37, Au Br4, 2(H2 O)
Sum formula	C42 H78 Au Br4 K O39	C42 H78 Au Br4 K O39
Mr	1762.72	1762.75
Dx,g cm-3	1.956	1.956
Z	2	2
Mu (mm-1)	9.299	9.299
F000	1760.0	1760.0
F000’	1751.88	
h,k,lmax	19,19,19	18,19,18
Nref	13057[6777]	12191
Tmin,Tmax	0.166,0.583	0.603,1.000
Tmin’	0.062	

Correction method= # Reported T Limits: Tmin=0.603 Tmax=1.000
AbsCorr = MULTI-SCAN

Data completeness= 1.80/0.93 Theta(max)= 79.841

R(reflections)= 0.0270(12112) wR2(reflections)= 0.0711(12191)

S = 1.067 Npar= 818

The following ALERTS were generated. Each ALERT has the format
test-name_ALERT_alert-type_alert-level.
Click on the hyperlinks for more details of the test.

Alert level B

```
PLAT035_ALERT_1_B _chemical_absolute_configuration Info  Not Given     Please Do !
PLAT414_ALERT_2_B Short Intra D-H..H-X       H25      ..H28A     .       1.89 Ang.
                                                   x,y,z  =     1_555 Check
PLAT415_ALERT_2_B Short Inter D-H..H-X       H7       ..H24      .       1.94 Ang.
                                                x,-1+y,z  =     1_545 Check
PLAT416_ALERT_2_B Short Intra D-H..H-D       H4       ..H8       .       1.67 Ang.
                                                   x,y,z  =     1_555 Check
PLAT416_ALERT_2_B Short Intra D-H..H-D       H34      ..H36A     .       1.58 Ang.
                                                x,y,-1+z  =     1_554 Check
PLAT417_ALERT_2_B Short Inter D-H..H-D       H14      ..H39A     .       1.92 Ang.
                                                   x,y,z  =     1_555 Check
PLAT417_ALERT_2_B Short Inter D-H..H-D       H14      ..H39B     .       2.05 Ang.
                                                   x,y,z  =     1_555 Check
PLAT420_ALERT_2_B D-H Bond Without Acceptor  O18      --H18      .     Please Check
PLAT420_ALERT_2_B D-H Bond Without Acceptor  O24      --H24      .     Please Check
PLAT420_ALERT_2_B D-H Bond Without Acceptor  O39      --H39A     .     Please Check
PLAT934_ALERT_3_B Number of (Iobs-Icalc)/Sigma(W) > 10 Outliers ..          3 Check
```

Alert level C

```
SHFSU01_ALERT_2_C  The absolute value of parameter shift to su ratio > 0.05
            Absolute value of the parameter shift to su ratio given   0.054
            Additional refinement cycles may be required.
PLAT220_ALERT_2_C NonSolvent   Resd 1  O   Ueq(max)/Ueq(min) Range        3.4 Ratio
PLAT222_ALERT_3_C NonSolvent Resd 1  H   Uiso(max)/Uiso(min) Range        4.4 Ratio
PLAT342_ALERT_3_C Low Bond Precision on  C-C Bonds ...............     0.0082 Ang.
PLAT415_ALERT_2_C Short Inter D-H..H-X       H38      ..H39A     .       2.04 Ang.
                                                 1+x,y,z  =     1_655 Check
PLAT417_ALERT_2_C Short Inter D-H..H-D       H4       ..H23      .       2.14 Ang.
                                         1-x,-1/2+y,1-z  =     2_646 Check
PLAT751_ALERT_4_C Bond    Calc     2.22000, Rep  2.2234(14) ......  Senseless s.u.
              K1       -H5              1_555   2_757 ........     #    8 Check
PLAT975_ALERT_2_C Check Calcd Resid. Dens.  0.90Ang From O25     .       0.53 eA-3
PLAT975_ALERT_2_C Check Calcd Resid. Dens.  0.87Ang From O39     .       0.49 eA-3
PLAT976_ALERT_2_C Check Calcd Resid. Dens.  0.89Ang From O39     .      -0.57 eA-3
PLAT976_ALERT_2_C Check Calcd Resid. Dens.  0.84Ang From O18     .      -0.50 eA-3
PLAT977_ALERT_2_C Check Negative Difference Density on H5        .      -0.40 eA-3
PLAT977_ALERT_2_C Check Negative Difference Density on H8        .      -0.39 eA-3
PLAT977_ALERT_2_C Check Negative Difference Density on H18       .      -0.45 eA-3
PLAT977_ALERT_2_C Check Negative Difference Density on H24       .      -0.35 eA-3
PLAT977_ALERT_2_C Check Negative Difference Density on H28       .      -0.31 eA-3
PLAT977_ALERT_2_C Check Negative Difference Density on H33       .      -0.38 eA-3
PLAT977_ALERT_2_C Check Negative Difference Density on H36A      .      -0.37 eA-3
PLAT977_ALERT_2_C Check Negative Difference Density on H39A      .      -0.46 eA-3
PLAT977_ALERT_2_C Check Negative Difference Density on H39B      .      -0.43 eA-3
```

Alert level G

```
PLAT002_ALERT_2_G Number of Distance or Angle Restraints on AtSite         19 Note
```

```
PLAT004_ALERT_5_G Polymeric Structure Found with Maximum Dimension          2 Info
PLAT007_ALERT_5_G Number of Unrefined Donor-H Atoms .............          23 Report
PLAT083_ALERT_2_G SHELXL Second Parameter in WGHT  Unusually Large       6.35 Why ?
PLAT142_ALERT_4_G s.u. on b - Axis Small or Missing .............     0.00010 Ang.
PLAT143_ALERT_4_G s.u. on c - Axis Small or Missing .............     0.00010 Ang.
PLAT169_ALERT_4_G The CIF-Embedded .res File Contains AFIX 1 Recds          1 Report
PLAT172_ALERT_4_G The CIF-Embedded .res File Contains DFIX Records          6 Report
PLAT173_ALERT_4_G The CIF-Embedded .res File Contains DANG Records          9 Report
PLAT176_ALERT_4_G The CIF-Embedded .res File Contains SADI Records          2 Report
PLAT303_ALERT_2_G Full Occupancy Atom H5        with # Connections       2.00 Check
PLAT779_ALERT_4_G Suspect or Irrelevant (Bond) Angle(s) in CIF ...      36.60 Deg.
              K1   -O5   -H5      2_747   1_555   1_555 ......     #   35 Check
PLAT794_ALERT_5_G Tentative Bond Valency for Au1       (III)     .       3.06 Info
PLAT860_ALERT_3_G Number of Least-Squares Restraints ............          21 Note
PLAT883_ALERT_1_G No Info/Value for _atom_sites_solution_primary .     Please Do !
PLAT912_ALERT_4_G Missing # of FCF Reflections Above STh/L=  0.600        206 Note
PLAT978_ALERT_2_G Number C-C Bonds with Positive Residual Density.          0 Info
```

---

0 **ALERT level A** = Most likely a serious problem - resolve or explain
11 **ALERT level B** = A potentially serious problem, consider carefully
20 **ALERT level C** = Check. Ensure it is not caused by an omission or oversight
17 **ALERT level G** = General information/check it is not something unexpected

2 ALERT type 1 CIF construction/syntax error, inconsistent or missing data
30 ALERT type 2 Indicator that the structure model may be wrong or deficient
4 ALERT type 3 Indicator that the structure quality may be low
9 ALERT type 4 Improvement, methodology, query or suggestion
3 ALERT type 5 Informative message, check

It is advisable to attempt to resolve as many as possible of the alerts in all categories. Often the minor alerts point to easily fixed oversights, errors and omissions in your CIF or refinement strategy, so attention to these fine details can be worthwhile. In order to resolve some of the more serious problems it may be necessary to carry out additional measurements or structure refinements. However, the purpose of your study may justify the reported deviations and the more serious of these should normally be commented upon in the discussion or experimental section of a paper or in the "special_details" fields of the CIF. checkCIF was carefully designed to identify outliers and unusual parameters, but every test has its limitations and alerts that are not important in a particular case may appear. Conversely, the absence of alerts does not guarantee there are no aspects of the results needing attention. It is up to the individual to critically assess their own results and, if necessary, seek expert advice.

Publication of your CIF in IUCr journals

A basic structural check has been run on your CIF. These basic checks will be run on all CIFs submitted for publication in IUCr journals (*Acta Crystallographica*, *Journal of Applied Crystallography*, *Journal of Synchrotron Radiation*); however, if you intend to submit to *Acta Crystallographica Section C* or *E* or *IUCrData*, you should make sure that full publication checks are run on the final version of your CIF prior to submission.

Publication of your CIF in other journals

Please refer to the *Notes for Authors* of the relevant journal for any special instructions relating to CIF submission.

PLATON version of 12/09/2022; check.def file version of 09/08/2022

Datablock cx2175 - ellipsoid plot

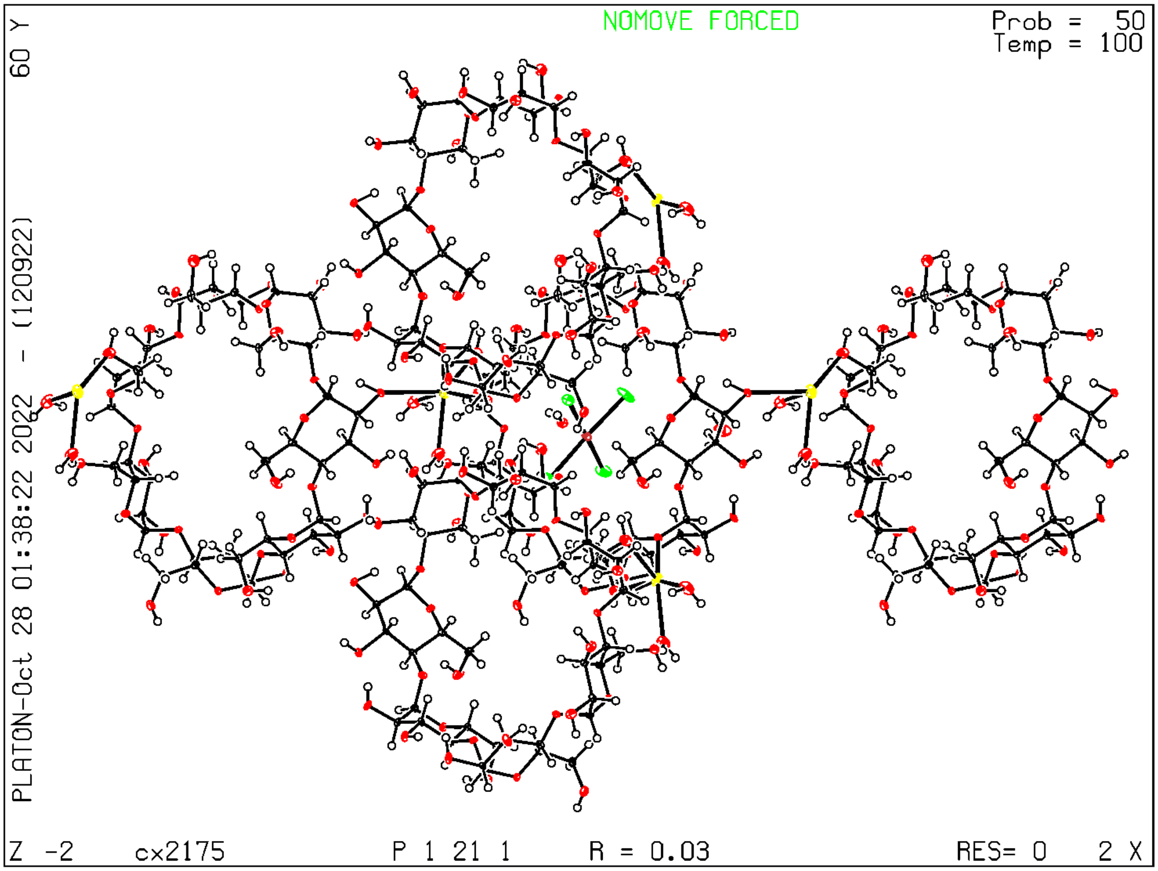

Supplement: Supplementary file 4 — Supplementary Data 1 Checkcif File for KAuBr4⊂β-CD Cocrystal [file 41467_2023_36591_MOESM4_ESM.pdf]
