# Supplementary material for: High-efficiency gold recovery by additive-induced supramolecular polymerization of β-cyclodextrin
Source: Nat Commun. 2023 Mar 9;14:1284. doi: 10.1038/s41467-023-36591-0 (PMC9998620; doi:10.1038/s41467-023-36591-0)

checkCIF/PLATON report

Structure factors have been supplied for datablock(s) gold7_cu1_auto

THIS REPORT IS FOR GUIDANCE ONLY. IF USED AS PART OF A REVIEW PROCEDURE FOR PUBLICATION, IT SHOULD NOT REPLACE THE EXPERTISE OF AN EXPERIENCED CRYSTALLOGRAPHIC REFEREE.

No syntax errors found.   CIF dictionary   Interpreting this report

Datablock: gold7_cu1_auto

```
Bond precision:   C-C = 0.0162 A           Wavelength=1.54184

Cell:          a=15.11370(18)     b=15.50618(18)     c=15.7047(2)
               alpha=88.8635(10)  beta=81.9417(10)   gamma=77.025(1)
Temperature:   100 K
```

	Calculated	Reported
Volume	3550.85(8)	3550.84(8)
Space group	P 1	P 1
Hall group	P 1	P 1
Moiety formula	2(C42 H70 O35), C12 H26 O3, Au Br4, ***(H2 O), H2 O0.50 [+ solv	Au Br4, 2(C42 H70 O35), 13.5(H2 O), C12 H26 O3, H2 O0.5
Sum formula	C96 H195 Au Br4 O87 [+ solvent]	C96 H195 Au Br4 O87
Mr	3258.10	3258.11
Dx,g cm-3	1.524	1.524
Z	1	1
Mu (mm-1)	4.205	4.205
F000	1686.0	1686.0
F000'	1684.76	
h,k,lmax	19,19,20	19,19,19
Nref	31028[15514]	27552
Tmin,Tmax	0.951,0.959	0.878,1.000
Tmin'	0.657	

```
Correction method= # Reported T Limits: Tmin=0.878 Tmax=1.000
AbsCorr = MULTI-SCAN

Data completeness= 1.78/0.89       Theta(max)= 80.214
```

R(reflections)= 0.0818(24664)

wR2(reflections)= 0.2238(27552)

S = 1.068 Npar= 1792

The following ALERTS were generated. Each ALERT has the format
test-name_ALERT_alert-type_alert-level.
Click on the hyperlinks for more details of the test.

Alert level B

```
PLAT035_ALERT_1_B _chemical_absolute_configuration Info  Not Given     Please Do !
PLAT245_ALERT_2_B U(iso) H77C      Smaller than U(eq) O77       by      0.052 Ang**2
PLAT416_ALERT_2_B Short Intra D-H..H-D       H24A      ..H62A     .       1.59 Ang.
                                                      x,y,z  =      1_555 Check
PLAT417_ALERT_2_B Short Inter D-H..H-D       H44       ..H62A     .       2.04 Ang.
                                                      x,y,z  =      1_555 Check
PLAT417_ALERT_2_B Short Inter D-H..H-D       H45       ..H75B     .       2.02 Ang.
                                                      x,y,z  =      1_555 Check
PLAT417_ALERT_2_B Short Inter D-H..H-D       H57       ..H61A     .       2.06 Ang.
                                                      x,y,z  =      1_555 Check
PLAT417_ALERT_2_B Short Inter D-H..H-D       H8        ..H36      .       1.99 Ang.
                                                      x,y,z  =      1_555 Check
PLAT417_ALERT_2_B Short Inter D-H..H-D       H43A      ..H81A     .       2.02 Ang.
                                                   x,-1+y,z  =      1_545 Check
PLAT417_ALERT_2_B Short Inter D-H..H-D       H58A      ..H76B     .       1.92 Ang.
                                                      x,y,z  =      1_555 Check
PLAT417_ALERT_2_B Short Inter D-H..H-D       H60A      ..H73B     .       1.93 Ang.
                                                x,-1+y,-1+z  =      1_544 Check
PLAT417_ALERT_2_B Short Inter D-H..H-D       H59A      ..H88A     .       2.00 Ang.
                                                    x,y,1+z  =      1_556 Check
PLAT417_ALERT_2_B Short Inter D-H..H-D       H67A      ..H75A     .       1.96 Ang.
                                                      x,y,z  =      1_555 Check
PLAT417_ALERT_2_B Short Inter D-H..H-D       H73A      ..H77C     .       2.02 Ang.
                                                      x,y,z  =      1_555 Check
PLAT417_ALERT_2_B Short Inter D-H..H-D       H76B      ..H88B     .       2.09 Ang.
                                                      x,y,z  =      1_555 Check
PLAT420_ALERT_2_B D-H Bond Without Acceptor  O52       --H52C     .     Please Check
PLAT420_ALERT_2_B D-H Bond Without Acceptor  O52       --H52D     .     Please Check
PLAT420_ALERT_2_B D-H Bond Without Acceptor  O57       --H57      .     Please Check
PLAT420_ALERT_2_B D-H Bond Without Acceptor  O61       --H61A     .     Please Check
PLAT420_ALERT_2_B D-H Bond Without Acceptor  O71       --H71A     .     Please Check
PLAT420_ALERT_2_B D-H Bond Without Acceptor  O75       --H75B     .     Please Check
PLAT420_ALERT_2_B D-H Bond Without Acceptor  O77       --H77A     .     Please Check
PLAT420_ALERT_2_B D-H Bond Without Acceptor  O77       --H77C     .     Please Check
PLAT420_ALERT_2_B D-H Bond Without Acceptor  O81       --H81A     .     Please Check
PLAT973_ALERT_2_B Check Calcd Positive Resid. Density on       Au1       1.68 eA-3
```

Alert level C

```
PLAT094_ALERT_2_C Ratio of Maximum / Minimum Residual Density ....       2.13 Report
PLAT213_ALERT_2_C Atom O65             has ADP max/min Ratio .....        3.4 prolat
PLAT213_ALERT_2_C Atom O66             has ADP max/min Ratio .....        3.6 prolat
PLAT213_ALERT_2_C Atom C58             has ADP max/min Ratio .....        3.7 prolat
PLAT213_ALERT_2_C Atom C34A            has ADP max/min Ratio .....        3.4 prolat
PLAT214_ALERT_2_C Atom O58       (Anion/Solvent) ADP max/min Ratio        4.7 prolat
```

```
PLAT220_ALERT_2_C NonSolvent   Resd 1  C   Ueq(max)/Ueq(min) Range       3.9 Ratio
PLAT220_ALERT_2_C NonSolvent   Resd 1  O   Ueq(max)/Ueq(min) Range       4.2 Ratio
PLAT221_ALERT_2_C Solv./Anion  Resd 2  O   Ueq(max)/Ueq(min) Range       4.7 Ratio
PLAT222_ALERT_3_C NonSolvent Resd 1  H   Uiso(max)/Uiso(min) Range       5.7 Ratio
PLAT223_ALERT_4_C Solv./Anion  Resd 2  H   Ueq(max)/Ueq(min) Range       6.5 Ratio
PLAT223_ALERT_4_C Solv./Anion  Resd16  H   Ueq(max)/Ueq(min) Range       9.3 Ratio
PLAT234_ALERT_4_C Large Hirshfeld Difference C35A     --C36      .      0.18 Ang.
PLAT234_ALERT_4_C Large Hirshfeld Difference O68      --C164     .      0.18 Ang.
PLAT234_ALERT_4_C Large Hirshfeld Difference C2       --C10      .      0.19 Ang.
PLAT234_ALERT_4_C Large Hirshfeld Difference C169     --C170     .      0.24 Ang.
PLAT241_ALERT_2_C High   'MainMol' Ueq as Compared to Neighbors of      C170 Check
PLAT242_ALERT_2_C Low    'MainMol' Ueq as Compared to Neighbors of       C60 Check
PLAT242_ALERT_2_C Low    'MainMol' Ueq as Compared to Neighbors of       C15 Check
PLAT244_ALERT_4_C Low    'Solvent' Ueq as Compared to Neighbors of       Au1 Check
PLAT260_ALERT_2_C Large Average Ueq of Residue Including       Au1     0.111 Check
PLAT260_ALERT_2_C Large Average Ueq of Residue Including       O75     0.174 Check
PLAT342_ALERT_3_C Low Bond Precision on  C-C Bonds ...............   0.01617 Ang.
PLAT414_ALERT_2_C Short Intra D-H..H-X       H22      ..H55      .      1.93 Ang.
                                                  x,y,z  =     1_555 Check
PLAT416_ALERT_2_C Short Intra D-H..H-D       H25      ..H36      .      1.95 Ang.
                                                  x,y,z  =     1_555 Check
PLAT417_ALERT_2_C Short Inter D-H..H-D       H51A     ..H56A     .      2.13 Ang.
                                                  x,y,z  =     1_555 Check
PLAT911_ALERT_3_C Missing FCF Refl Between Thmin & STh/L=    0.600          6 Report
PLAT971_ALERT_2_C Check Calcd Resid. Dens.  1.47Ang From Br             2.41 eA-3
PLAT976_ALERT_2_C Check Calcd Resid. Dens.  0.59Ang From O76     .     -0.63 eA-3
PLAT976_ALERT_2_C Check Calcd Resid. Dens.  0.58Ang From O76     .     -0.52 eA-3
PLAT976_ALERT_2_C Check Calcd Resid. Dens.  0.70Ang From O76     .     -0.50 eA-3
PLAT977_ALERT_2_C Check Negative Difference Density on H7        .     -0.46 eA-3
PLAT977_ALERT_2_C Check Negative Difference Density on H25       .     -0.37 eA-3
PLAT977_ALERT_2_C Check Negative Difference Density on H59A      .     -0.39 eA-3
PLAT977_ALERT_2_C Check Negative Difference Density on H76A      .     -0.34 eA-3
PLAT977_ALERT_2_C Check Negative Difference Density on H83       .     -0.32 eA-3
```

Alert level G

```
PLAT002_ALERT_2_G Number of Distance or Angle Restraints on AtSite        10 Note
PLAT003_ALERT_2_G Number of Uiso or Uij Restrained non-H Atoms ...         1 Report
PLAT007_ALERT_5_G Number of Unrefined Donor-H Atoms ..............        75 Report
PLAT042_ALERT_1_G Calc. and Reported MoietyFormula Strings  Differ    Please Check
PLAT072_ALERT_2_G SHELXL First  Parameter in WGHT  Unusually Large      0.13 Report
PLAT083_ALERT_2_G SHELXL Second Parameter in WGHT  Unusually Large     11.28 Why ?
PLAT154_ALERT_1_G The s.u.'s on the Cell Angles are Equal ..(Note)     0.001 Degree
PLAT169_ALERT_4_G The CIF-Embedded .res File Contains AFIX 1 Recds        21 Report
PLAT176_ALERT_4_G The CIF-Embedded .res File Contains SADI Records        11 Report
PLAT186_ALERT_4_G The CIF-Embedded .res File Contains ISOR Records         1 Report
PLAT187_ALERT_4_G The CIF-Embedded .res File Contains RIGU Records         1 Report
PLAT300_ALERT_4_G Atom Site Occupancy of O74        Constrained at       0.5 Check
PLAT300_ALERT_4_G Atom Site Occupancy of H74A       Constrained at       0.5 Check
PLAT300_ALERT_4_G Atom Site Occupancy of H74B       Constrained at       0.5 Check
PLAT300_ALERT_4_G Atom Site Occupancy of O77        Constrained at       0.5 Check
PLAT300_ALERT_4_G Atom Site Occupancy of H77A       Constrained at       0.5 Check
PLAT300_ALERT_4_G Atom Site Occupancy of H77B       Constrained at       0.5 Check
PLAT301_ALERT_3_G Main Residue  Disorder .............(Resd  1  )         5% Note
PLAT302_ALERT_4_G Anion/Solvent/Minor-Residue Disorder (Resd 13  )      100% Note
PLAT302_ALERT_4_G Anion/Solvent/Minor-Residue Disorder (Resd 16  )      100% Note
PLAT304_ALERT_4_G Non-Integer Number of Atoms in ..... (Resd 13  )      1.50 Check
```

```
PLAT304_ALERT_4_G Non-Integer Number of Atoms in ..... (Resd 16  )       2.50 Check
PLAT410_ALERT_2_G Short Intra H...H Contact  H24      ..H39      .       1.96 Ang.
                                                  x,y,z  =     1_555 Check
PLAT410_ALERT_2_G Short Intra H...H Contact  H24      ..H39A     .       2.09 Ang.
                                                  x,y,z  =     1_555 Check
PLAT410_ALERT_2_G Short Intra H...H Contact  H36A     ..H46      .       1.95 Ang.
                                                  x,y,z  =     1_555 Check
PLAT410_ALERT_2_G Short Intra H...H Contact  H46      ..H36B     .       2.06 Ang.
                                                  x,y,z  =     1_555 Check
PLAT416_ALERT_2_G Short Intra D-H..H-D       H44      ..H72A     .       1.69 Ang.
                                                  x,y,z  =     1_555 Check
PLAT417_ALERT_2_G Short Inter D-H..H-D       H56B     ..H74A     .       1.59 Ang.
                                                  x,y,z  =     1_555 Check
PLAT417_ALERT_2_G Short Inter D-H..H-D       H71B     ..H72B     .       1.89 Ang.
                                                  x,y,z  =     1_555 Check
PLAT605_ALERT_4_G Largest Solvent Accessible VOID in the Structure        215 A**3
PLAT721_ALERT_1_G Bond    Calc     0.85000, Rep     0.84000 Dev...       0.01 Ang.
              O85      -H85            1_555   1_555 ........    #  369 Check
PLAT791_ALERT_4_G Model has Chirality at C1A        (Sohnke SpGr)          R Verify
PLAT791_ALERT_4_G Model has Chirality at C3         (Sohnke SpGr)          R Verify
PLAT791_ALERT_4_G Model has Chirality at C4         (Sohnke SpGr)          S Verify
PLAT791_ALERT_4_G Model has Chirality at C5         (Sohnke SpGr)          S Verify
PLAT791_ALERT_4_G Model has Chirality at C6         (Sohnke SpGr)          S Verify
PLAT791_ALERT_4_G Model has Chirality at C7         (Sohnke SpGr)          S Verify
PLAT791_ALERT_4_G Model has Chirality at C8         (Sohnke SpGr)          R Verify
PLAT791_ALERT_4_G Model has Chirality at C9         (Sohnke SpGr)          S Verify
PLAT791_ALERT_4_G Model has Chirality at C11        (Sohnke SpGr)          R Verify
PLAT791_ALERT_4_G Model has Chirality at C12        (Sohnke SpGr)          S Verify
PLAT791_ALERT_4_G Model has Chirality at C13        (Sohnke SpGr)          S Verify
PLAT791_ALERT_4_G Model has Chirality at C14        (Sohnke SpGr)          R Verify
PLAT791_ALERT_4_G Model has Chirality at C17        (Sohnke SpGr)          S Verify
PLAT791_ALERT_4_G Model has Chirality at C18        (Sohnke SpGr)          S Verify
PLAT791_ALERT_4_G Model has Chirality at C19        (Sohnke SpGr)          R Verify
PLAT791_ALERT_4_G Model has Chirality at C22        (Sohnke SpGr)          S Verify
PLAT791_ALERT_4_G Model has Chirality at C25        (Sohnke SpGr)          R Verify
PLAT791_ALERT_4_G Model has Chirality at C29        (Sohnke SpGr)          S Verify
PLAT791_ALERT_4_G Model has Chirality at C30        (Sohnke SpGr)          S Verify
PLAT791_ALERT_4_G Model has Chirality at C31        (Sohnke SpGr)          R Verify
PLAT791_ALERT_4_G Model has Chirality at C43        (Sohnke SpGr)          S Verify
PLAT791_ALERT_4_G Model has Chirality at C44        (Sohnke SpGr)          R Verify
PLAT791_ALERT_4_G Model has Chirality at C45        (Sohnke SpGr)          S Verify
PLAT791_ALERT_4_G Model has Chirality at C46        (Sohnke SpGr)          S Verify
PLAT791_ALERT_4_G Model has Chirality at C47        (Sohnke SpGr)          R Verify
PLAT791_ALERT_4_G Model has Chirality at C58        (Sohnke SpGr)          S Verify
PLAT791_ALERT_4_G Model has Chirality at C59        (Sohnke SpGr)          R Verify
PLAT791_ALERT_4_G Model has Chirality at C60        (Sohnke SpGr)          S Verify
PLAT791_ALERT_4_G Model has Chirality at C61        (Sohnke SpGr)          S Verify
PLAT791_ALERT_4_G Model has Chirality at C62        (Sohnke SpGr)          R Verify
PLAT791_ALERT_4_G Model has Chirality at C65        (Sohnke SpGr)          S Verify
PLAT791_ALERT_4_G Model has Chirality at C66        (Sohnke SpGr)          R Verify
PLAT791_ALERT_4_G Model has Chirality at C67        (Sohnke SpGr)          S Verify
PLAT791_ALERT_4_G Model has Chirality at C68        (Sohnke SpGr)          S Verify
PLAT791_ALERT_4_G Model has Chirality at C69        (Sohnke SpGr)          R Verify
PLAT791_ALERT_4_G Model has Chirality at C72        (Sohnke SpGr)          S Verify
PLAT791_ALERT_4_G Model has Chirality at C73        (Sohnke SpGr)          R Verify
PLAT791_ALERT_4_G Model has Chirality at C74        (Sohnke SpGr)          S Verify
PLAT791_ALERT_4_G Model has Chirality at C75        (Sohnke SpGr)          S Verify
```

```
PLAT791_ALERT_4_G Model has Chirality at C76         (Sohnke SpGr)          R Verify
PLAT791_ALERT_4_G Model has Chirality at C79         (Sohnke SpGr)          S Verify
PLAT791_ALERT_4_G Model has Chirality at C81         (Sohnke SpGr)          R Verify
PLAT791_ALERT_4_G Model has Chirality at C98         (Sohnke SpGr)          S Verify
PLAT791_ALERT_4_G Model has Chirality at C99         (Sohnke SpGr)          R Verify
PLAT791_ALERT_4_G Model has Chirality at C100        (Sohnke SpGr)          S Verify
PLAT791_ALERT_4_G Model has Chirality at C101        (Sohnke SpGr)          S Verify
PLAT791_ALERT_4_G Model has Chirality at C102        (Sohnke SpGr)          R Verify
PLAT791_ALERT_4_G Model has Chirality at C105        (Sohnke SpGr)          R Verify
PLAT791_ALERT_4_G Model has Chirality at C106        (Sohnke SpGr)          S Verify
PLAT791_ALERT_4_G Model has Chirality at C107        (Sohnke SpGr)          S Verify
PLAT791_ALERT_4_G Model has Chirality at C108        (Sohnke SpGr)          R Verify
PLAT791_ALERT_4_G Model has Chirality at C109        (Sohnke SpGr)          S Verify
PLAT791_ALERT_4_G Model has Chirality at C113        (Sohnke SpGr)          S Verify
PLAT791_ALERT_4_G Model has Chirality at C114        (Sohnke SpGr)          R Verify
PLAT791_ALERT_4_G Model has Chirality at C115        (Sohnke SpGr)          S Verify
PLAT791_ALERT_4_G Model has Chirality at C116        (Sohnke SpGr)          S Verify
PLAT791_ALERT_4_G Model has Chirality at C117        (Sohnke SpGr)          R Verify
PLAT791_ALERT_4_G Model has Chirality at C127        (Sohnke SpGr)          R Verify
PLAT791_ALERT_4_G Model has Chirality at C128        (Sohnke SpGr)          S Verify
PLAT791_ALERT_4_G Model has Chirality at C129        (Sohnke SpGr)          S Verify
PLAT791_ALERT_4_G Model has Chirality at C130        (Sohnke SpGr)          R Verify
PLAT791_ALERT_4_G Model has Chirality at C131        (Sohnke SpGr)          S Verify
PLAT791_ALERT_4_G Model has Chirality at C134        (Sohnke SpGr)          S Verify
PLAT791_ALERT_4_G Model has Chirality at C135        (Sohnke SpGr)          R Verify
PLAT794_ALERT_5_G Tentative Bond Valency for Au1       (III)     .       3.03 Info
PLAT860_ALERT_3_G Number of Least-Squares Restraints ............         50 Note
PLAT883_ALERT_1_G No Info/Value for _atom_sites_solution_primary .     Please Do !
PLAT910_ALERT_3_G Missing # of FCF Reflection(s) Below Theta(Min).          3 Note
PLAT912_ALERT_4_G Missing # of FCF Reflections Above STh/L=  0.600        842 Note
PLAT978_ALERT_2_G Number C-C Bonds with Positive Residual Density.          0 Info
```

```
  0 ALERT level A = Most likely a serious problem - resolve or explain
 24 ALERT level B = A potentially serious problem, consider carefully
 36 ALERT level C = Check. Ensure it is not caused by an omission or oversight
101 ALERT level G = General information/check it is not something unexpected

  5 ALERT type 1 CIF construction/syntax error, inconsistent or missing data
 61 ALERT type 2 Indicator that the structure model may be wrong or deficient
  6 ALERT type 3 Indicator that the structure quality may be low
 87 ALERT type 4 Improvement, methodology, query or suggestion
  2 ALERT type 5 Informative message, check
```

It is advisable to attempt to resolve as many as possible of the alerts in all categories. Often the minor alerts point to easily fixed oversights, errors and omissions in your CIF or refinement strategy, so attention to these fine details can be worthwhile. In order to resolve some of the more serious problems it may be necessary to carry out additional measurements or structure refinements. However, the purpose of your study may justify the reported deviations and the more serious of these should normally be commented upon in the discussion or experimental section of a paper or in the "special_details" fields of the CIF. checkCIF was carefully designed to identify outliers and unusual parameters, but every test has its limitations and alerts that are not important in a particular case may appear. Conversely, the absence of alerts does not guarantee there are no aspects of the results needing attention. It is up to the individual to critically assess their own results and, if necessary, seek expert advice.

Publication of your CIF in IUCr journals

A basic structural check has been run on your CIF. These basic checks will be run on all CIFs submitted for publication in IUCr journals (*Acta Crystallographica*, *Journal of Applied Crystallography*, *Journal of Synchrotron Radiation*); however, if you intend to submit to *Acta Crystallographica Section C* or *E* or *IUCrData*, you should make sure that full publication checks are run on the final version of your CIF prior to submission.

Publication of your CIF in other journals

Please refer to the *Notes for Authors* of the relevant journal for any special instructions relating to CIF submission.

PLATON version of 12/09/2022; check.def file version of 09/08/2022

Datablock gold7_cu1_auto - ellipsoid plot

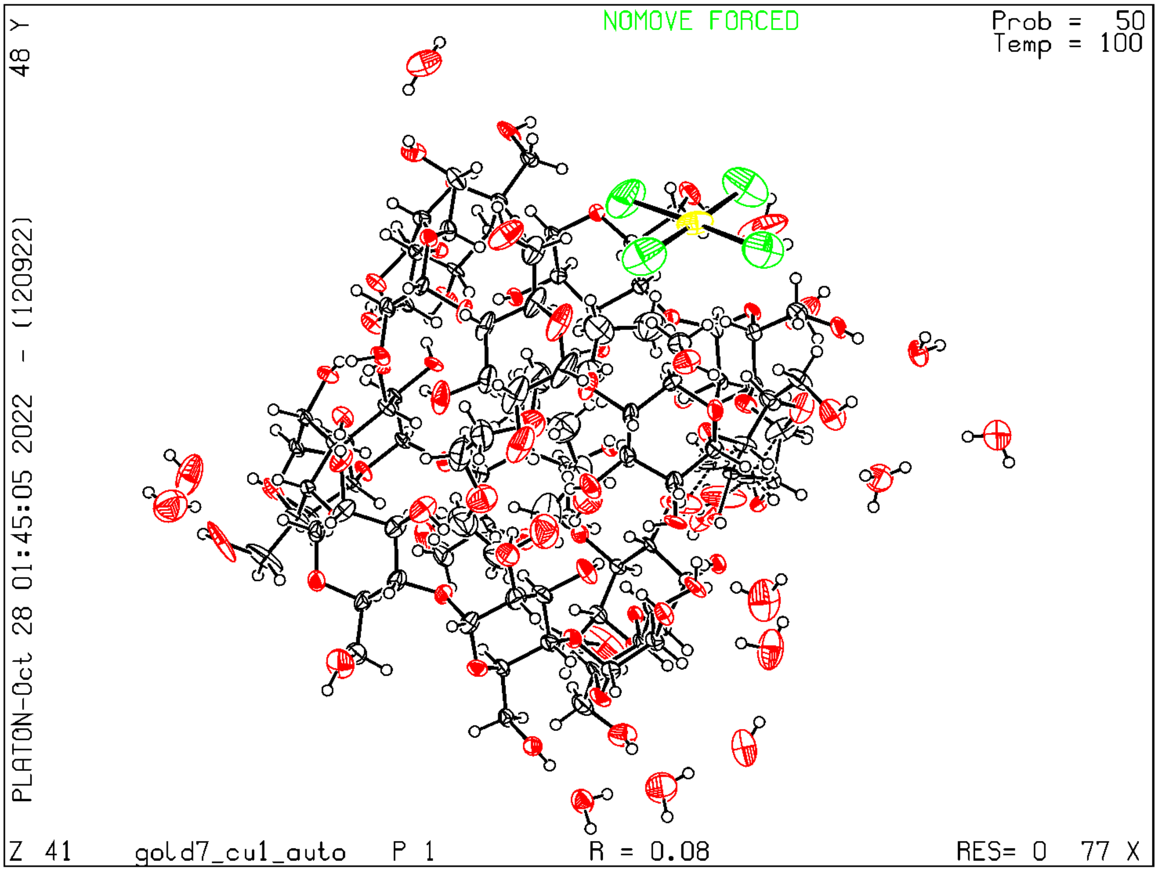

Supplement: Supplementary file 5 — Supplementary Data 2 Checkcif File for HAuBr4•DBC⊂2β-CD Cocrystal [file 41467_2023_36591_MOESM5_ESM.pdf]
