# Supplementary material for: High-efficiency gold recovery by additive-induced supramolecular polymerization of β-cyclodextrin
Source: Nat Commun. 2023 Mar 9;14:1284. doi: 10.1038/s41467-023-36591-0 (PMC9998620; doi:10.1038/s41467-023-36591-0)

checkCIF/PLATON report

Structure factors have been supplied for datablock(s) gold6_phase1_auto

THIS REPORT IS FOR GUIDANCE ONLY. IF USED AS PART OF A REVIEW PROCEDURE FOR PUBLICATION, IT SHOULD NOT REPLACE THE EXPERTISE OF AN EXPERIENCED CRYSTALLOGRAPHIC REFEREE.

No syntax errors found. CIF dictionary Interpreting this report

Datablock: gold6_phase1_auto

Bond precision:	C-C = 0.0326 A		Wavelength=0.71073
Cell:	a=15.1182(8)	b=15.3034(9)	c=15.5384(8)
	alpha=87.704(4)	beta=81.920(4)	gamma=76.368(5)
Temperature:	100 K		

	Calculated	Reported
Volume	3458.9(3)	3458.9(3)
Space group	P 1	P 1
Hall group	P 1	P 1
Moiety formula	2(C42 H70 O35), Au Br4, 2(C6 H14 O), 7(H2 O), H3 O [+ solvent]	Au Br4, 2(C42 H70 O35), 7(H2 O), H3 O, 2(C6 H14 O)
Sum formula	C96 H185 Au Br4 O80 [+ solvent]	C96 H185 Au Br4 O80
Mr	3136.02	3136.04
Dx,g cm-3	1.505	1.506
Z	1	1
Mu (mm-1)	2.322	2.322
F000	1620.0	1620.0
F000’	1617.90	
h,k,lmax	21,21,22	21,21,21
Nref	41874[20937]	27941
Tmin,Tmax	0.946,0.977	0.440,1.000
Tmin’	0.793	

Correction method= # Reported T Limits: Tmin=0.440 Tmax=1.000
AbsCorr = MULTI-SCAN

Data completeness= 1.33/0.67 Theta(max)= 30.409

R(reflections)= 0.1437(12437) wR2(reflections)= 0.3894(27941)

S = 1.017 Npar= 1710

The following ALERTS were generated. Each ALERT has the format
test-name_ALERT_alert-type_alert-level.
Click on the hyperlinks for more details of the test.

Alert level B

```
RINTA01_ALERT_3_B  The value of Rint is greater than 0.18
            Rint given   0.227
PLAT020_ALERT_3_B The Value of Rint is Greater Than 0.12 .........      0.227 Report
PLAT035_ALERT_1_B _chemical_absolute_configuration Info  Not Given     Please Do !
PLAT084_ALERT_3_B High wR2 Value (i.e. > 0.25) ...................       0.39 Report
PLAT201_ALERT_2_B Isotropic non-H Atoms in Main Residue(s) .......          2 Report
              O1         O16
PLAT342_ALERT_3_B Low Bond Precision on  C-C Bonds ...............    0.03257 Ang.
PLAT355_ALERT_3_B Long   O-H (X0.82,N0.98A)  O169      - H16C     .       1.09 Ang.
PLAT415_ALERT_2_B Short Inter D-H..H-X       H50       ..H62      .       1.99 Ang.
                                                 -1+x,y,z  =     1_455 Check
PLAT415_ALERT_2_B Short Inter D-H..H-X       H21A      ..H153     .       1.93 Ang.
                                                    x,y,z  =     1_555 Check
PLAT416_ALERT_2_B Short Intra D-H..H-D       H129      ..H153     .       1.76 Ang.
                                                    x,y,z  =     1_555 Check
PLAT416_ALERT_2_B Short Intra D-H..H-D       H75       ..H160     .       1.68 Ang.
                                                    x,y,z  =     1_555 Check
PLAT417_ALERT_2_B Short Inter D-H..H-D       H134      ..H151     .       1.93 Ang.
                                                  1+x,y,z  =     1_655 Check
PLAT417_ALERT_2_B Short Inter D-H..H-D       H47       ..H140     .       1.88 Ang.
                                              x,-1+y,-1+z  =     1_544 Check
PLAT417_ALERT_2_B Short Inter D-H..H-D       H47       ..H153     .       1.95 Ang.
                                                 x,y,-1+z  =     1_554 Check
PLAT417_ALERT_2_B Short Inter D-H..H-D       H53       ..H147     .       2.02 Ang.
                                                    x,y,z  =     1_555 Check
PLAT417_ALERT_2_B Short Inter D-H..H-D       H75       ..H135     .       2.06 Ang.
                                                 x,y,-1+z  =     1_554 Check
PLAT417_ALERT_2_B Short Inter D-H..H-D       H79       ..H139     .       2.08 Ang.
                                                 x,y,-1+z  =     1_554 Check
PLAT417_ALERT_2_B Short Inter D-H..H-D       H15E      ..H59      .       1.87 Ang.
                                                    x,y,z  =     1_555 Check
PLAT417_ALERT_2_B Short Inter D-H..H-D       H15E      ..H137     .       1.91 Ang.
                                                    x,y,z  =     1_555 Check
PLAT417_ALERT_2_B Short Inter D-H..H-D       H15G      ..H53      .       2.09 Ang.
                                                    x,y,z  =     1_555 Check
PLAT417_ALERT_2_B Short Inter D-H..H-D       H15G      ..H78      .       2.04 Ang.
                                                 -1+x,y,z  =     1_455 Check
PLAT417_ALERT_2_B Short Inter D-H..H-D       H15H      ..H78      .       1.91 Ang.
                                                 -1+x,y,z  =     1_455 Check
PLAT417_ALERT_2_B Short Inter D-H..H-D       H16E      ..H16I     .       1.96 Ang.
                                                  x,1+y,z  =     1_565 Check
PLAT417_ALERT_2_B Short Inter D-H..H-D       H16I      ..H131     .       1.87 Ang.
                                                    x,y,z  =     1_555 Check
PLAT420_ALERT_2_B D-H Bond Without Acceptor  O158      --H15F     .    Please Check
PLAT420_ALERT_2_B D-H Bond Without Acceptor  O169      --H16A     .    Please Check
```

```
PLAT420_ALERT_2_B D-H Bond Without Acceptor  O169     --H16B     .     Please Check
PLAT420_ALERT_2_B D-H Bond Without Acceptor  O169     --H16C     .     Please Check
PLAT420_ALERT_2_B D-H Bond Without Acceptor  O164     --H16F     .     Please Check
PLAT420_ALERT_2_B D-H Bond Without Acceptor  O164     --H16G     .     Please Check
PLAT420_ALERT_2_B D-H Bond Without Acceptor  O166     --H16H     .     Please Check
PLAT420_ALERT_2_B D-H Bond Without Acceptor  O167     --H16J     .     Please Check
PLAT420_ALERT_2_B D-H Bond Without Acceptor  O167     --H16K     .     Please Check
PLAT420_ALERT_2_B D-H Bond Without Acceptor  O74      --H74      .     Please Check
PLAT420_ALERT_2_B D-H Bond Without Acceptor  O143     --H143     .     Please Check
PLAT420_ALERT_2_B D-H Bond Without Acceptor  O153     --H153     .     Please Check
PLAT420_ALERT_2_B D-H Bond Without Acceptor  O156     --H156     .     Please Check
PLAT971_ALERT_2_B Check Calcd Resid. Dens.  1.16Ang From Br2A          2.65 eA-3
PLAT987_ALERT_1_B The Flack x is >> 0 -  Do a BASF/TWIN Refinement     Please Check
```

Alert level C

```
PLAT026_ALERT_3_C Ratio Observed / Unique Reflections (too) Low ..        45% Check
PLAT082_ALERT_2_C High R1 Value .................................       0.14 Report
PLAT094_ALERT_2_C Ratio of Maximum / Minimum Residual Density ....       2.05 Report
PLAT213_ALERT_2_C Atom O160            has ADP max/min Ratio .....        3.2 oblate
PLAT244_ALERT_4_C Low    'Solvent' Ueq as Compared to Neighbors of        Au1 Check
PLAT245_ALERT_2_C U(iso) H16C      Smaller than U(eq) O169      by      0.028 Ang**2
PLAT250_ALERT_2_C Large U3/U1 Ratio for Average U(i,j) Tensor ....        2.2 Note
PLAT250_ALERT_2_C Large U3/U1 Ratio for Average U(i,j) Tensor ....        2.6 Note
PLAT250_ALERT_2_C Large U3/U1 Ratio for Average U(i,j) Tensor ....        2.1 Note
PLAT313_ALERT_2_C Oxygen with Three Covalent Bonds (rare) ........       O169 Check
PLAT360_ALERT_2_C Short  C(sp3)-C(sp3) Bond  C84      - C86      .       1.42 Ang.
PLAT360_ALERT_2_C Short  C(sp3)-C(sp3) Bond  C115     - C116     .       1.43 Ang.
PLAT360_ALERT_2_C Short  C(sp3)-C(sp3) Bond  C11      - C12      .       1.43 Ang.
PLAT360_ALERT_2_C Short  C(sp3)-C(sp3) Bond  C23      - C24      .       1.42 Ang.
PLAT360_ALERT_2_C Short  C(sp3)-C(sp3) Bond  C30      - C48      .       1.38 Ang.
PLAT360_ALERT_2_C Short  C(sp3)-C(sp3) Bond  C62      - C63      .       1.43 Ang.
PLAT361_ALERT_2_C Long   C(sp3)-C(sp3) Bond  C15      - C21      .       1.67 Ang.
PLAT410_ALERT_2_C Short Intra H...H Contact  H8       ..H41      .       1.94 Ang.
                                                      x,y,z  =     1_555 Check
PLAT410_ALERT_2_C Short Intra H...H Contact  H9       ..H19      .       1.99 Ang.
                                                      x,y,z  =     1_555 Check
PLAT410_ALERT_2_C Short Intra H...H Contact  H27      ..H31      .       1.97 Ang.
                                                      x,y,z  =     1_555 Check
PLAT414_ALERT_2_C Short Intra D-H..H-X       H31      ..H49      .       1.90 Ang.
                                                      x,y,z  =     1_555 Check
PLAT414_ALERT_2_C Short Intra D-H..H-X       H71A     ..H72      .       1.95 Ang.
                                                      x,y,z  =     1_555 Check
PLAT414_ALERT_2_C Short Intra D-H..H-X       H77B     ..H78      .       1.96 Ang.
                                                      x,y,z  =     1_555 Check
PLAT415_ALERT_2_C Short Inter D-H..H-X       H14C     ..H59      .       2.09 Ang.
                                                      x,y,z  =     1_555 Check
PLAT415_ALERT_2_C Short Inter D-H..H-X       H28B     ..H135     .       2.13 Ang.
                                                      x,y,z  =     1_555 Check
PLAT416_ALERT_2_C Short Intra D-H..H-D       H5A      ..H132     .       1.97 Ang.
                                                      x,y,z  =     1_555 Check
PLAT417_ALERT_2_C Short Inter D-H..H-D       H75      ..H132     .       2.10 Ang.
                                                   x,y,-1+z  =     1_554 Check
PLAT417_ALERT_2_C Short Inter D-H..H-D       H80      ..H139     .       2.11 Ang.
                                                   x,y,-1+z  =     1_554 Check
PLAT910_ALERT_3_C Missing # of FCF Reflection(s) Below Theta(Min).          7 Note
PLAT911_ALERT_3_C Missing FCF Refl Between Thmin & STh/L=    0.600         53 Report
```

```
PLAT915_ALERT_3_C No Flack x Check Done: Low Friedel Pair Coverage         56 %
PLAT918_ALERT_3_C Reflection(s) with I(obs) much Smaller I(calc) .          4 Check
PLAT971_ALERT_2_C Check Calcd Resid. Dens.  1.12Ang From Br6A            1.73 eA-3
PLAT971_ALERT_2_C Check Calcd Resid. Dens.  0.42Ang From Br4A            1.64 eA-3
PLAT971_ALERT_2_C Check Calcd Resid. Dens.  1.29Ang From Br2A            1.64 eA-3
PLAT975_ALERT_2_C Check Calcd Resid. Dens.  0.66Ang From O49      .      0.94 eA-3
PLAT977_ALERT_2_C Check Negative Difference Density on H15        .     -0.41 eA-3
PLAT977_ALERT_2_C Check Negative Difference Density on H16C       .     -0.52 eA-3
PLAT977_ALERT_2_C Check Negative Difference Density on H16K       .     -0.31 eA-3
PLAT977_ALERT_2_C Check Negative Difference Density on H49        .     -0.38 eA-3
PLAT977_ALERT_2_C Check Negative Difference Density on H74        .     -0.51 eA-3
PLAT977_ALERT_2_C Check Negative Difference Density on H84        .     -0.31 eA-3
PLAT977_ALERT_2_C Check Negative Difference Density on H123       .     -0.36 eA-3
PLAT977_ALERT_2_C Check Negative Difference Density on H147       .     -0.39 eA-3
PLAT977_ALERT_2_C Check Negative Difference Density on H2A        .     -0.34 eA-3
```

Alert level G

```
PLAT002_ALERT_2_G Number of Distance or Angle Restraints on AtSite         20 Note
PLAT003_ALERT_2_G Number of Uiso or Uij Restrained non-H Atoms ...        182 Report
PLAT007_ALERT_5_G Number of Unrefined Donor-H Atoms .............          59 Report
PLAT033_ALERT_4_G Flack x Value Deviates > 3.0 * sigma from Zero .      0.049 Note
PLAT042_ALERT_1_G Calc. and Reported MoietyFormula Strings  Differ     Please Check
PLAT072_ALERT_2_G SHELXL First  Parameter in WGHT  Unusually Large       0.20 Report
PLAT169_ALERT_4_G The CIF-Embedded .res File Contains AFIX 1 Recds         13 Report
PLAT171_ALERT_4_G The CIF-Embedded .res File Contains EADP Records          3 Report
PLAT172_ALERT_4_G The CIF-Embedded .res File Contains DFIX Records          3 Report
PLAT174_ALERT_4_G The CIF-Embedded .res File Contains FLAT Records          1 Report
PLAT176_ALERT_4_G The CIF-Embedded .res File Contains SADI Records         11 Report
PLAT178_ALERT_4_G The CIF-Embedded .res File Contains SIMU Records          1 Report
PLAT186_ALERT_4_G The CIF-Embedded .res File Contains ISOR Records          9 Report
PLAT187_ALERT_4_G The CIF-Embedded .res File Contains RIGU Records          1 Report
PLAT302_ALERT_4_G Anion/Solvent/Minor-Residue Disorder (Resd  3  )        80% Note
PLAT302_ALERT_4_G Anion/Solvent/Minor-Residue Disorder (Resd  4  )        29% Note
PLAT302_ALERT_4_G Anion/Solvent/Minor-Residue Disorder (Resd  5  )        29% Note
PLAT410_ALERT_2_G Short Intra H...H Contact  H2B      ..H22       .       2.14 Ang.
                                                        x,y,z  =     1_555 Check
PLAT411_ALERT_2_G Short Inter H...H Contact  H18      ..H2A       .       2.09 Ang.
                                                        x,y,z  =     1_555 Check
PLAT412_ALERT_2_G Short Intra XH3 .. XHn     H15      ..H7AB      .       1.77 Ang.
                                                        x,y,z  =     1_555 Check
PLAT412_ALERT_2_G Short Intra XH3 .. XHn     H22      ..H29C      .       1.54 Ang.
                                                        x,y,z  =     1_555 Check
PLAT413_ALERT_2_G Short Inter XH3 .. XHn     H25      ..H29A      .       1.01 Ang.
                                                        x,y,z  =     1_555 Check
PLAT413_ALERT_2_G Short Inter XH3 .. XHn     H25      ..H29B      .       2.13 Ang.
                                                        x,y,z  =     1_555 Check
PLAT413_ALERT_2_G Short Inter XH3 .. XHn     H25      ..H29C      .       1.75 Ang.
                                                        x,y,z  =     1_555 Check
PLAT413_ALERT_2_G Short Inter XH3 .. XHn     H3AB     ..H25       .       1.95 Ang.
                                                        x,y,z  =     1_555 Check
PLAT432_ALERT_2_G Short Inter X...Y Contact  Br8A     ..C150      .       3.11 Ang.
                                                        x,y,z  =     1_555 Check
PLAT432_ALERT_2_G Short Inter X...Y Contact  O23      ..C3        .       2.96 Ang.
                                                      x,y,1+z  =     1_556 Check
PLAT432_ALERT_2_G Short Inter X...Y Contact  O29      ..C29       .       3.02 Ang.
                                                        x,y,z  =     1_555 Check
```

```
PLAT432_ALERT_2_G Short Inter X...Y Contact  C25       ..C29       .        2.64 Ang.
                                                        x,y,z  =      1_555 Check
PLAT605_ALERT_4_G Largest Solvent Accessible VOID in the Structure        290 A**3
PLAT720_ALERT_4_G Number of Unusual/Non-Standard Labels ..........         11 Note
PLAT790_ALERT_4_G Centre of Gravity not Within Unit Cell: Resd.  #          8 Note
              H2 O
PLAT790_ALERT_4_G Centre of Gravity not Within Unit Cell: Resd.  #         10 Note
              H2 O
PLAT790_ALERT_4_G Centre of Gravity not Within Unit Cell: Resd.  #         12 Note
              H2 O
PLAT790_ALERT_4_G Centre of Gravity not Within Unit Cell: Resd.  #         13 Note
              H3 O
PLAT791_ALERT_4_G Model has Chirality at C1          (Sohnke SpGr)          R Verify
PLAT791_ALERT_4_G Model has Chirality at C5          (Sohnke SpGr)          S Verify
PLAT791_ALERT_4_G Model has Chirality at C8          (Sohnke SpGr)          S Verify
PLAT791_ALERT_4_G Model has Chirality at C9          (Sohnke SpGr)          S Verify
PLAT791_ALERT_4_G Model has Chirality at C10         (Sohnke SpGr)          R Verify
PLAT791_ALERT_4_G Model has Chirality at C11         (Sohnke SpGr)          S Verify
PLAT791_ALERT_4_G Model has Chirality at C12         (Sohnke SpGr)          S Verify
PLAT791_ALERT_4_G Model has Chirality at C13         (Sohnke SpGr)          R Verify
PLAT791_ALERT_4_G Model has Chirality at C16         (Sohnke SpGr)          S Verify
PLAT791_ALERT_4_G Model has Chirality at C17         (Sohnke SpGr)          R Verify
PLAT791_ALERT_4_G Model has Chirality at C18         (Sohnke SpGr)          S Verify
PLAT791_ALERT_4_G Model has Chirality at C19         (Sohnke SpGr)          S Verify
PLAT791_ALERT_4_G Model has Chirality at C20         (Sohnke SpGr)          R Verify
PLAT791_ALERT_4_G Model has Chirality at C23         (Sohnke SpGr)          R Verify
PLAT791_ALERT_4_G Model has Chirality at C24         (Sohnke SpGr)          S Verify
PLAT791_ALERT_4_G Model has Chirality at C25         (Sohnke SpGr)          S Verify
PLAT791_ALERT_4_G Model has Chirality at C26         (Sohnke SpGr)          R Verify
PLAT791_ALERT_4_G Model has Chirality at C27         (Sohnke SpGr)          S Verify
PLAT791_ALERT_4_G Model has Chirality at C30         (Sohnke SpGr)          R Verify
PLAT791_ALERT_4_G Model has Chirality at C31         (Sohnke SpGr)          S Verify
PLAT791_ALERT_4_G Model has Chirality at C32         (Sohnke SpGr)          S Verify
PLAT791_ALERT_4_G Model has Chirality at C33         (Sohnke SpGr)          R Verify
PLAT791_ALERT_4_G Model has Chirality at C34         (Sohnke SpGr)          S Verify
PLAT791_ALERT_4_G Model has Chirality at C37         (Sohnke SpGr)          R Verify
PLAT791_ALERT_4_G Model has Chirality at C38         (Sohnke SpGr)          S Verify
PLAT791_ALERT_4_G Model has Chirality at C39         (Sohnke SpGr)          S Verify
PLAT791_ALERT_4_G Model has Chirality at C40         (Sohnke SpGr)          R Verify
PLAT791_ALERT_4_G Model has Chirality at C41         (Sohnke SpGr)          S Verify
PLAT791_ALERT_4_G Model has Chirality at C61         (Sohnke SpGr)          S Verify
PLAT791_ALERT_4_G Model has Chirality at C62         (Sohnke SpGr)          R Verify
PLAT791_ALERT_4_G Model has Chirality at C63         (Sohnke SpGr)          S Verify
PLAT791_ALERT_4_G Model has Chirality at C64         (Sohnke SpGr)          S Verify
PLAT791_ALERT_4_G Model has Chirality at C65         (Sohnke SpGr)          R Verify
PLAT791_ALERT_4_G Model has Chirality at C68         (Sohnke SpGr)          S Verify
PLAT791_ALERT_4_G Model has Chirality at C70         (Sohnke SpGr)          R Verify
PLAT791_ALERT_4_G Model has Chirality at C82         (Sohnke SpGr)          S Verify
PLAT791_ALERT_4_G Model has Chirality at C83         (Sohnke SpGr)          S Verify
PLAT791_ALERT_4_G Model has Chirality at C84         (Sohnke SpGr)          R Verify
PLAT791_ALERT_4_G Model has Chirality at C85         (Sohnke SpGr)          S Verify
PLAT791_ALERT_4_G Model has Chirality at C86         (Sohnke SpGr)          S Verify
PLAT791_ALERT_4_G Model has Chirality at C89         (Sohnke SpGr)          S Verify
PLAT791_ALERT_4_G Model has Chirality at C90         (Sohnke SpGr)          S Verify
PLAT791_ALERT_4_G Model has Chirality at C92         (Sohnke SpGr)          R Verify
PLAT791_ALERT_4_G Model has Chirality at C93         (Sohnke SpGr)          S Verify
PLAT791_ALERT_4_G Model has Chirality at C94         (Sohnke SpGr)          R Verify
```

```
PLAT791_ALERT_4_G Model has Chirality at C95         (Sohnke SpGr)          R Verify
PLAT791_ALERT_4_G Model has Chirality at C96         (Sohnke SpGr)          S Verify
PLAT791_ALERT_4_G Model has Chirality at C97         (Sohnke SpGr)          R Verify
PLAT791_ALERT_4_G Model has Chirality at C98         (Sohnke SpGr)          S Verify
PLAT791_ALERT_4_G Model has Chirality at C101        (Sohnke SpGr)          S Verify
PLAT791_ALERT_4_G Model has Chirality at C102        (Sohnke SpGr)          R Verify
PLAT791_ALERT_4_G Model has Chirality at C103        (Sohnke SpGr)          S Verify
PLAT791_ALERT_4_G Model has Chirality at C104        (Sohnke SpGr)          S Verify
PLAT791_ALERT_4_G Model has Chirality at C105        (Sohnke SpGr)          R Verify
PLAT791_ALERT_4_G Model has Chirality at C107        (Sohnke SpGr)          S Verify
PLAT791_ALERT_4_G Model has Chirality at C108        (Sohnke SpGr)          R Verify
PLAT791_ALERT_4_G Model has Chirality at C109        (Sohnke SpGr)          S Verify
PLAT791_ALERT_4_G Model has Chirality at C110        (Sohnke SpGr)          S Verify
PLAT791_ALERT_4_G Model has Chirality at C111        (Sohnke SpGr)          R Verify
PLAT791_ALERT_4_G Model has Chirality at C114        (Sohnke SpGr)          R Verify
PLAT791_ALERT_4_G Model has Chirality at C115        (Sohnke SpGr)          S Verify
PLAT791_ALERT_4_G Model has Chirality at C116        (Sohnke SpGr)          S Verify
PLAT791_ALERT_4_G Model has Chirality at C117        (Sohnke SpGr)          R Verify
PLAT791_ALERT_4_G Model has Chirality at C118        (Sohnke SpGr)          S Verify
PLAT791_ALERT_4_G Model has Chirality at C122        (Sohnke SpGr)          S Verify
PLAT791_ALERT_4_G Model has Chirality at C123        (Sohnke SpGr)          R Verify
PLAT791_ALERT_4_G Model has Chirality at C125        (Sohnke SpGr)          S Verify
PLAT791_ALERT_4_G Model has Chirality at C126        (Sohnke SpGr)          R Verify
PLAT791_ALERT_4_G Model has Chirality at C148        (Sohnke SpGr)          R Verify
PLAT791_ALERT_4_G Model has Chirality at C162        (Sohnke SpGr)          S Verify
PLAT860_ALERT_3_G Number of Least-Squares Restraints .............       2709 Note
PLAT912_ALERT_4_G Missing # of FCF Reflections Above STh/L=  0.600       4635 Note
PLAT941_ALERT_3_G Average HKL Measurement Multiplicity ...........        3.9 Low
PLAT978_ALERT_2_G Number C-C Bonds with Positive Residual Density.          0 Info
```

---

  0 **ALERT level A** = Most likely a serious problem - resolve or explain
 39 **ALERT level B** = A potentially serious problem, consider carefully
 45 **ALERT level C** = Check. Ensure it is not caused by an omission or oversight
109 **ALERT level G** = General information/check it is not something unexpected

  3 ALERT type 1 CIF construction/syntax error, inconsistent or missing data
 87 ALERT type 2 Indicator that the structure model may be wrong or deficient
 12 ALERT type 3 Indicator that the structure quality may be low
 90 ALERT type 4 Improvement, methodology, query or suggestion
  1 ALERT type 5 Informative message, check

It is advisable to attempt to resolve as many as possible of the alerts in all categories. Often the minor alerts point to easily fixed oversights, errors and omissions in your CIF or refinement strategy, so attention to these fine details can be worthwhile. In order to resolve some of the more serious problems it may be necessary to carry out additional measurements or structure refinements. However, the purpose of your study may justify the reported deviations and the more serious of these should normally be commented upon in the discussion or experimental section of a paper or in the "special_details" fields of the CIF. checkCIF was carefully designed to identify outliers and unusual parameters, but every test has its limitations and alerts that are not important in a particular case may appear. Conversely, the absence of alerts does not guarantee there are no aspects of the results needing attention. It is up to the individual to critically assess their own results and, if necessary, seek expert advice.

Publication of your CIF in IUCr journals

A basic structural check has been run on your CIF. These basic checks will be run on all CIFs submitted for publication in IUCr journals (*Acta Crystallographica*, *Journal of Applied Crystallography*, *Journal of Synchrotron Radiation*); however, if you intend to submit to *Acta Crystallographica Section C* or *E* or *IUCrData*, you should make sure that full publication checks are run on the final version of your CIF prior to submission.

Publication of your CIF in other journals

Please refer to the *Notes for Authors* of the relevant journal for any special instructions relating to CIF submission.

PLATON version of 12/09/2022; check.def file version of 09/08/2022

Datablock gold6_phase1_auto - ellipsoid plot

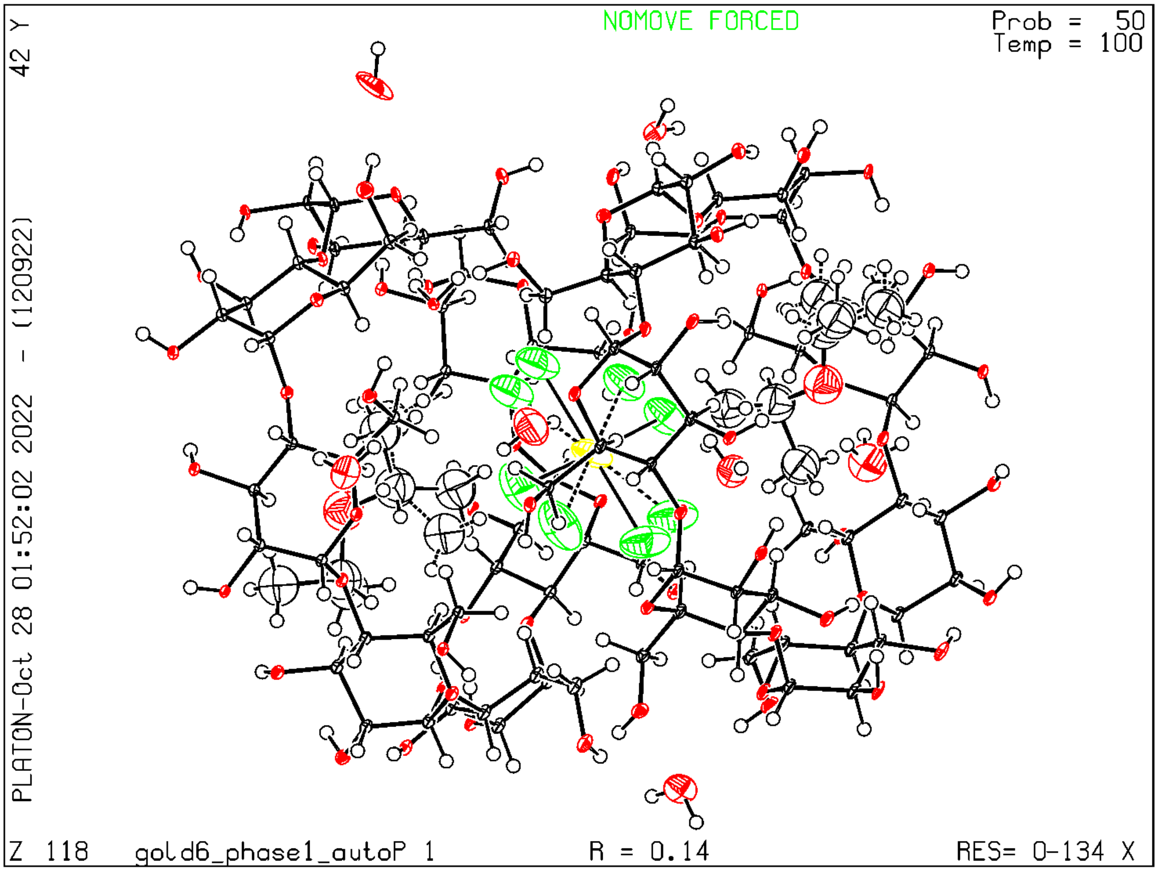

Supplement: Supplementary file 6 — Supplementary Data 3 Checkcif File for HAuBr4•2(iPr2O)⊂2β-CD Cocrystal (Cocrystal A) [file 41467_2023_36591_MOESM6_ESM.pdf]
