# Supplementary material for: High-efficiency gold recovery by additive-induced supramolecular polymerization of β-cyclodextrin
Source: Nat Commun. 2023 Mar 9;14:1284. doi: 10.1038/s41467-023-36591-0 (PMC9998620; doi:10.1038/s41467-023-36591-0)

checkCIF/PLATON report

You have not supplied any structure factors. As a result the full set of tests cannot be run.

THIS REPORT IS FOR GUIDANCE ONLY. IF USED AS PART OF A REVIEW PROCEDURE FOR PUBLICATION, IT SHOULD NOT REPLACE THE EXPERTISE OF AN EXPERIENCED CRYSTALLOGRAPHIC REFEREE.

No syntax errors found. CIF dictionary Interpreting this report

Datablock: gold6-phase2_auto

```
Bond precision:   C-C = 0.0177 A          Wavelength=0.71073

Cell:             a=15.7110(7)     b=24.3931(7)       c=18.9812(7)
                  alpha=90         beta=108.544(4)    gamma=90
Temperature:      100 K
```

	Calculated	Reported
Volume	6896.7(5)	6896.7(5)
Space group	P 21	P 1 21 1
Hall group	P 2yb	P 2yb
Moiety formula	4(C42 H70 O35), Au Br4, 44(H2 O), 2(H2.50 O) [+ solvent]	2(C42 H70 O35), 22(H2 O), H2.5 O, 0.5(Au Br4)
Sum formula	C168 H373 Au Br4 O186 [+ solvent]	C84 H186.50 Au0.50 Br2 O93
Mr	5886.26	2943.12
Dx,g cm-3	1.417	1.417
Z	1	2
Mu (mm-1)	1.228	1.228
F000	3088.0	3088.0
F000’	3087.08	
h,k,lmax	18,29,22	18,29,22
Nref	25241[12940]	25231
Tmin,Tmax	0.985,0.988	0.782,1.000
Tmin’	0.940	

```
Correction method= # Reported T Limits: Tmin=0.782 Tmax=1.000
AbsCorr = MULTI-SCAN

Data completeness= 1.95/1.00        Theta(max)= 25.348
```

```
R(reflections)= 0.1150( 18756)                          wR2(reflections)=
                                                        0.3237( 25231)
S = 1.208                    Npar= 1670
```

The following ALERTS were generated. Each ALERT has the format
test-name_ALERT_alert-type_alert-level.
Click on the hyperlinks for more details of the test.

Alert level B

```
RINTA01_ALERT_3_B  The value of Rint is greater than 0.18
            Rint given   0.182
PLAT020_ALERT_3_B The Value of Rint is Greater Than 0.12 .........      0.182 Report
PLAT035_ALERT_1_B _chemical_absolute_configuration Info  Not Given     Please Do !
PLAT245_ALERT_2_B U(iso) H17C      Smaller than U(eq) O175      by      0.078 Ang**2
PLAT260_ALERT_2_B Large Average Ueq of Residue Including       O10      0.363 Check
PLAT260_ALERT_2_B Large Average Ueq of Residue Including       O17      0.301 Check
PLAT415_ALERT_2_B Short Inter D-H..H-X       H58       ..H99      .       1.90 Ang.
                                       2-x,-1/2+y,2-z  =      2_747 Check
PLAT415_ALERT_2_B Short Inter D-H..H-X       H16T      ..H101     .       1.95 Ang.
                                                x,y,z  =      1_555 Check
PLAT417_ALERT_2_B Short Inter D-H..H-D       H55       ..H154     .       1.93 Ang.
                                        2-x,1/2+y,2-z  =      2_757 Check
PLAT417_ALERT_2_B Short Inter D-H..H-D       H57       ..H145     .       2.02 Ang.
                                                x,y,z  =      1_555 Check
PLAT417_ALERT_2_B Short Inter D-H..H-D       H61       ..H158     .       2.08 Ang.
                                                x,y,z  =      1_555 Check
PLAT417_ALERT_2_B Short Inter D-H..H-D       H10A      ..H20B     .       2.03 Ang.
                                                x,y,z  =      1_555 Check
PLAT417_ALERT_2_B Short Inter D-H..H-D       H11A      ..H20B     .       1.97 Ang.
                                                x,y,z  =      1_555 Check
PLAT417_ALERT_2_B Short Inter D-H..H-D       H16E      ..H134     .       1.99 Ang.
                                            1+x,y,1+z  =      1_656 Check
PLAT417_ALERT_2_B Short Inter D-H..H-D       H16G      ..H80      .       1.97 Ang.
                                       2-x,-1/2+y,2-z  =      2_747 Check
PLAT417_ALERT_2_B Short Inter D-H..H-D       H16I      ..H17F     .       1.82 Ang.
                                              x,y,1+z  =      1_556 Check
PLAT417_ALERT_2_B Short Inter D-H..H-D       H16I      ..H148     .       1.87 Ang.
                                                x,y,z  =      1_555 Check
PLAT417_ALERT_2_B Short Inter D-H..H-D       H16J      ..H17F     .       1.93 Ang.
                                              x,y,1+z  =      1_556 Check
PLAT417_ALERT_2_B Short Inter D-H..H-D       H16N      ..H17K     .       2.02 Ang.
                                                x,y,z  =      1_555 Check
PLAT417_ALERT_2_B Short Inter D-H..H-D       H16O      ..H17E     .       1.85 Ang.
                                                x,y,z  =      1_555 Check
PLAT417_ALERT_2_B Short Inter D-H..H-D       H16O      ..H60      .       1.92 Ang.
                                        2-x,1/2+y,2-z  =      2_757 Check
PLAT417_ALERT_2_B Short Inter D-H..H-D       H16P      ..H17E     .       1.92 Ang.
                                                x,y,z  =      1_555 Check
PLAT417_ALERT_2_B Short Inter D-H..H-D       H16P      ..H60      .       1.91 Ang.
                                        2-x,1/2+y,2-z  =      2_757 Check
PLAT417_ALERT_2_B Short Inter D-H..H-D       H16S      ..H17D     .       1.97 Ang.
                                                x,y,z  =      1_555 Check
PLAT417_ALERT_2_B Short Inter D-H..H-D       H16S      ..H130     .       1.91 Ang.
                                        1-x,1/2+y,1-z  =      2_656 Check
```

```
PLAT417_ALERT_2_B Short Inter D-H..H-D       H17F     ..H17O     .       1.87 Ang.
                                                   x,y,-1+z  =      1_554 Check
PLAT417_ALERT_2_B Short Inter D-H..H-D       H17G     ..H60      .       2.03 Ang.
                                                -1+x,y,-1+z  =      1_454 Check
PLAT417_ALERT_2_B Short Inter D-H..H-D       H17J     ..H72      .       2.02 Ang.
                                              2-x,1/2+y,1-z  =      2_756 Check
PLAT417_ALERT_2_B Short Inter D-H..H-D       H17K     ..H143     .       2.06 Ang.
                                                      x,y,z  =      1_555 Check
PLAT417_ALERT_2_B Short Inter D-H..H-D       H17L     ..H157     .       1.90 Ang.
                                                      x,y,z  =      1_555 Check
PLAT417_ALERT_2_B Short Inter D-H..H-D       H17M     ..H157     .       2.09 Ang.
                                                      x,y,z  =      1_555 Check
PLAT417_ALERT_2_B Short Inter D-H..H-D       H17B     ..H73      .       1.90 Ang.
                                                      x,y,z  =      1_555 Check
PLAT420_ALERT_2_B D-H Bond Without Acceptor  O10      --H10B     .    Please Check
PLAT420_ALERT_2_B D-H Bond Without Acceptor  O11      --H11A     .    Please Check
PLAT420_ALERT_2_B D-H Bond Without Acceptor  O11      --H11B     .    Please Check
PLAT420_ALERT_2_B D-H Bond Without Acceptor  O15      --H15G     .    Please Check
PLAT420_ALERT_2_B D-H Bond Without Acceptor  O15      --H15H     .    Please Check
PLAT420_ALERT_2_B D-H Bond Without Acceptor  O161     --H16D     .    Please Check
PLAT420_ALERT_2_B D-H Bond Without Acceptor  O164     --H16J     .    Please Check
PLAT420_ALERT_2_B D-H Bond Without Acceptor  O165     --H16L     .    Please Check
PLAT420_ALERT_2_B D-H Bond Without Acceptor  O166     --H16M     .    Please Check
PLAT420_ALERT_2_B D-H Bond Without Acceptor  O169     --H16T     .    Please Check
PLAT420_ALERT_2_B D-H Bond Without Acceptor  O172     --H17H     .    Please Check
PLAT420_ALERT_2_B D-H Bond Without Acceptor  O173     --H17J     .    Please Check
PLAT420_ALERT_2_B D-H Bond Without Acceptor  O174     --H17L     .    Please Check
PLAT420_ALERT_2_B D-H Bond Without Acceptor  O174     --H17M     .    Please Check
PLAT420_ALERT_2_B D-H Bond Without Acceptor  O17      --H17P     .    Please Check
PLAT420_ALERT_2_B D-H Bond Without Acceptor  O17      --H17Q     .    Please Check
PLAT420_ALERT_2_B D-H Bond Without Acceptor  O20      --H20A     .    Please Check
PLAT420_ALERT_2_B D-H Bond Without Acceptor  O58      --H58      .    Please Check
PLAT420_ALERT_2_B D-H Bond Without Acceptor  O130     --H130     .    Please Check
PLAT987_ALERT_1_B The Flack x is >> 0 -  Do a BASF/TWIN Refinement    Please Check
```

Alert level C

```
PLAT082_ALERT_2_C High R1 Value ..................................       0.12 Report
PLAT084_ALERT_3_C High wR2 Value (i.e. > 0.25) ...................       0.32 Report
PLAT090_ALERT_3_C Poor Data / Parameter Ratio (Zmax > 18) ........       7.75 Note
PLAT094_ALERT_2_C Ratio of Maximum / Minimum Residual Density ....       3.31 Report
PLAT220_ALERT_2_C NonSolvent   Resd 2  O   Ueq(max)/Ueq(min) Range        3.2 Ratio
PLAT222_ALERT_3_C NonSolvent Resd 1  H   Uiso(max)/Uiso(min) Range        4.5 Ratio
PLAT222_ALERT_3_C NonSolvent Resd 2  H   Uiso(max)/Uiso(min) Range        4.2 Ratio
PLAT234_ALERT_4_C Large Hirshfeld Difference O73      --C22      .       0.17 Ang.
PLAT234_ALERT_4_C Large Hirshfeld Difference O80      --C79      .       0.21 Ang.
PLAT234_ALERT_4_C Large Hirshfeld Difference O6       --C151     .       0.19 Ang.
PLAT234_ALERT_4_C Large Hirshfeld Difference O148     --C147     .       0.18 Ang.
PLAT234_ALERT_4_C Large Hirshfeld Difference C110     --C111     .       0.16 Ang.
PLAT260_ALERT_2_C Large Average Ueq of Residue Including      Au1      0.134 Check
PLAT260_ALERT_2_C Large Average Ueq of Residue Including      O11      0.213 Check
PLAT260_ALERT_2_C Large Average Ueq of Residue Including      O15      0.171 Check
PLAT260_ALERT_2_C Large Average Ueq of Residue Including      O20      0.159 Check
PLAT260_ALERT_2_C Large Average Ueq of Residue Including     O171      0.114 Check
PLAT260_ALERT_2_C Large Average Ueq of Residue Including     O172      0.141 Check
PLAT260_ALERT_2_C Large Average Ueq of Residue Including     O175      0.128 Check
PLAT260_ALERT_2_C Large Average Ueq of Residue Including     O178      0.104 Check
```

```
PLAT313_ALERT_2_C Oxygen with Three Covalent Bonds (rare) ........       O175 Check
PLAT342_ALERT_3_C Low Bond Precision on  C-C Bonds ...............    0.01774 Ang.
PLAT354_ALERT_3_C Short  O-H (X0.82,N0.98A)  O17      - H17Q     .       0.69 Ang.
PLAT410_ALERT_2_C Short Intra H...H Contact  H86      ..H90      .       1.95 Ang.
                                                  x,y,z  =      1_555 Check
PLAT414_ALERT_2_C Short Intra D-H..H-X       H118     ..H134     .       1.97 Ang.
                                                  x,y,z  =      1_555 Check
PLAT415_ALERT_2_C Short Inter D-H..H-X       H50      ..H154     .       2.00 Ang.
                                          2-x,1/2+y,2-z  =      2_757 Check
PLAT416_ALERT_2_C Short Intra D-H..H-D       H7A      ..H139     .       1.94 Ang.
                                                  x,y,z  =      1_555 Check
PLAT417_ALERT_2_C Short Inter D-H..H-D       H6A      ..H16H     .       2.14 Ang.
                                                  x,y,z  =      1_555 Check
PLAT417_ALERT_2_C Short Inter D-H..H-D       H145     ..H156     .       2.11 Ang.
                                          2-x,1/2+y,2-z  =      2_757 Check
PLAT417_ALERT_2_C Short Inter D-H..H-D       H15E     ..H16C     .       2.13 Ang.
                                         2-x,-1/2+y,1-z  =      2_746 Check
PLAT417_ALERT_2_C Short Inter D-H..H-D       H16H     ..H80      .       2.12 Ang.
                                         2-x,-1/2+y,2-z  =      2_747 Check
PLAT417_ALERT_2_C Short Inter D-H..H-D       H16P     ..H134     .       2.13 Ang.
                                          1-x,1/2+y,1-z  =      2_656 Check
PLAT417_ALERT_2_C Short Inter D-H..H-D       H16R     ..H77      .       2.12 Ang.
                                         2-x,-1/2+y,1-z  =      2_746 Check
PLAT790_ALERT_4_C Centre of Gravity not Within Unit Cell: Resd.  #          1 Note
              C42 H70 O35
```

Alert level G

```
PLAT002_ALERT_2_G Number of Distance or Angle Restraints on AtSite          5 Note
PLAT007_ALERT_5_G Number of Unrefined Donor-H Atoms .............          89 Report
PLAT012_ALERT_1_G No       _shelx_res_checksum Found in CIF ......     Please Check
PLAT014_ALERT_1_G No       _shelx_fab_checksum Found in CIF ......     Please Check
PLAT033_ALERT_4_G Flack x Value Deviates > 3.0 * sigma from Zero .      0.058 Note
PLAT042_ALERT_1_G Calc. and Reported MoietyFormula Strings  Differ     Please Check
PLAT045_ALERT_1_G Calculated and Reported Z Differ by a Factor ...      0.500 Check
PLAT072_ALERT_2_G SHELXL First  Parameter in WGHT  Unusually Large       0.20 Report
PLAT153_ALERT_1_G The s.u.'s on the Cell Axes   are Equal ..(Note)     0.0007 Ang.
PLAT169_ALERT_4_G The CIF-Embedded .res File Contains AFIX 1 Recds         43 Report
PLAT174_ALERT_4_G The CIF-Embedded .res File Contains FLAT Records          1 Report
PLAT176_ALERT_4_G The CIF-Embedded .res File Contains SADI Records          2 Report
PLAT178_ALERT_4_G The CIF-Embedded .res File Contains SIMU Records          2 Report
PLAT187_ALERT_4_G The CIF-Embedded .res File Contains RIGU Records          2 Report
PLAT300_ALERT_4_G Atom Site Occupancy of Au1        Constrained at        0.5 Check
PLAT300_ALERT_4_G Atom Site Occupancy of Br1        Constrained at        0.5 Check
PLAT300_ALERT_4_G Atom Site Occupancy of Br2        Constrained at        0.5 Check
PLAT300_ALERT_4_G Atom Site Occupancy of Br4        Constrained at        0.5 Check
PLAT300_ALERT_4_G Atom Site Occupancy of Br16       Constrained at        0.5 Check
PLAT300_ALERT_4_G Atom Site Occupancy of H17C       Constrained at        0.5 Check
PLAT302_ALERT_4_G Anion/Solvent/Minor-Residue Disorder (Resd  3  )       100% Note
PLAT304_ALERT_4_G Non-Integer Number of Atoms in ..... (Resd  3  )       2.50 Check
PLAT304_ALERT_4_G Non-Integer Number of Atoms in ..... (Resd 25  )       3.50 Check
PLAT606_ALERT_4_G Solvent Accessible VOID(S) in Structure ........          ! Info
PLAT721_ALERT_1_G Bond    Calc     0.83000, Rep     0.81490 Dev...       0.02 Ang.
              O10      -H10B            1_555   1_555 ........     #  351 Check
PLAT722_ALERT_1_G Angle   Calc      112.00, Rep      113.20 Dev...       1.20 Degree
              H10A    -O10     -H10B       1_555  1_555  1_555     #  601 Check
PLAT790_ALERT_4_G Centre of Gravity not Within Unit Cell: Resd.  #          3 Note
```

```
              Au Br4
PLAT790_ALERT_4_G Centre of Gravity not Within Unit Cell: Resd.  #         10 Note
              H2 O
PLAT790_ALERT_4_G Centre of Gravity not Within Unit Cell: Resd.  #         11 Note
              H2 O
PLAT790_ALERT_4_G Centre of Gravity not Within Unit Cell: Resd.  #         12 Note
              H2 O
PLAT790_ALERT_4_G Centre of Gravity not Within Unit Cell: Resd.  #         13 Note
              H2 O
PLAT790_ALERT_4_G Centre of Gravity not Within Unit Cell: Resd.  #         14 Note
              H2 O
PLAT790_ALERT_4_G Centre of Gravity not Within Unit Cell: Resd.  #         15 Note
              H2 O
PLAT790_ALERT_4_G Centre of Gravity not Within Unit Cell: Resd.  #         16 Note
              H2 O
PLAT790_ALERT_4_G Centre of Gravity not Within Unit Cell: Resd.  #         22 Note
              H2 O
PLAT790_ALERT_4_G Centre of Gravity not Within Unit Cell: Resd.  #         24 Note
              H2 O
PLAT790_ALERT_4_G Centre of Gravity not Within Unit Cell: Resd.  #         26 Note
              H2 O
PLAT791_ALERT_4_G Model has Chirality at C6          (Sohnke SpGr)          R Verify
PLAT791_ALERT_4_G Model has Chirality at C7          (Sohnke SpGr)          R Verify
PLAT791_ALERT_4_G Model has Chirality at C9          (Sohnke SpGr)          S Verify
PLAT791_ALERT_4_G Model has Chirality at C11         (Sohnke SpGr)          S Verify
PLAT791_ALERT_4_G Model has Chirality at C12         (Sohnke SpGr)          S Verify
PLAT791_ALERT_4_G Model has Chirality at C13         (Sohnke SpGr)          S Verify
PLAT791_ALERT_4_G Model has Chirality at C14         (Sohnke SpGr)          R Verify
PLAT791_ALERT_4_G Model has Chirality at C15         (Sohnke SpGr)          S Verify
PLAT791_ALERT_4_G Model has Chirality at C16         (Sohnke SpGr)          S Verify
PLAT791_ALERT_4_G Model has Chirality at C17         (Sohnke SpGr)          R Verify
PLAT791_ALERT_4_G Model has Chirality at C19         (Sohnke SpGr)          R Verify
PLAT791_ALERT_4_G Model has Chirality at C20         (Sohnke SpGr)          S Verify
PLAT791_ALERT_4_G Model has Chirality at C21         (Sohnke SpGr)          S Verify
PLAT791_ALERT_4_G Model has Chirality at C22         (Sohnke SpGr)          R Verify
PLAT791_ALERT_4_G Model has Chirality at C23         (Sohnke SpGr)          S Verify
PLAT791_ALERT_4_G Model has Chirality at C27         (Sohnke SpGr)          S Verify
PLAT791_ALERT_4_G Model has Chirality at C28         (Sohnke SpGr)          R Verify
PLAT791_ALERT_4_G Model has Chirality at C29         (Sohnke SpGr)          S Verify
PLAT791_ALERT_4_G Model has Chirality at C30         (Sohnke SpGr)          S Verify
PLAT791_ALERT_4_G Model has Chirality at C31         (Sohnke SpGr)          R Verify
PLAT791_ALERT_4_G Model has Chirality at C33         (Sohnke SpGr)          S Verify
PLAT791_ALERT_4_G Model has Chirality at C34         (Sohnke SpGr)          R Verify
PLAT791_ALERT_4_G Model has Chirality at C35         (Sohnke SpGr)          S Verify
PLAT791_ALERT_4_G Model has Chirality at C36         (Sohnke SpGr)          S Verify
PLAT791_ALERT_4_G Model has Chirality at C37         (Sohnke SpGr)          R Verify
PLAT791_ALERT_4_G Model has Chirality at C41         (Sohnke SpGr)          S Verify
PLAT791_ALERT_4_G Model has Chirality at C42         (Sohnke SpGr)          R Verify
PLAT791_ALERT_4_G Model has Chirality at C43         (Sohnke SpGr)          S Verify
PLAT791_ALERT_4_G Model has Chirality at C44         (Sohnke SpGr)          S Verify
PLAT791_ALERT_4_G Model has Chirality at C45         (Sohnke SpGr)          R Verify
PLAT791_ALERT_4_G Model has Chirality at C47         (Sohnke SpGr)          S Verify
PLAT791_ALERT_4_G Model has Chirality at C48         (Sohnke SpGr)          R Verify
PLAT791_ALERT_4_G Model has Chirality at C49         (Sohnke SpGr)          S Verify
PLAT791_ALERT_4_G Model has Chirality at C50         (Sohnke SpGr)          S Verify
PLAT791_ALERT_4_G Model has Chirality at C51         (Sohnke SpGr)          R Verify
PLAT791_ALERT_4_G Model has Chirality at C83         (Sohnke SpGr)          S Verify
```

```
PLAT791_ALERT_4_G Model has Chirality at C84         (Sohnke SpGr)          S Verify
PLAT791_ALERT_4_G Model has Chirality at C85         (Sohnke SpGr)          R Verify
PLAT791_ALERT_4_G Model has Chirality at C86         (Sohnke SpGr)          S Verify
PLAT791_ALERT_4_G Model has Chirality at C87         (Sohnke SpGr)          S Verify
PLAT791_ALERT_4_G Model has Chirality at C90         (Sohnke SpGr)          S Verify
PLAT791_ALERT_4_G Model has Chirality at C91         (Sohnke SpGr)          S Verify
PLAT791_ALERT_4_G Model has Chirality at C92         (Sohnke SpGr)          R Verify
PLAT791_ALERT_4_G Model has Chirality at C94         (Sohnke SpGr)          S Verify
PLAT791_ALERT_4_G Model has Chirality at C95         (Sohnke SpGr)          R Verify
PLAT791_ALERT_4_G Model has Chirality at C96         (Sohnke SpGr)          S Verify
PLAT791_ALERT_4_G Model has Chirality at C97         (Sohnke SpGr)          R Verify
PLAT791_ALERT_4_G Model has Chirality at C98         (Sohnke SpGr)          R Verify
PLAT791_ALERT_4_G Model has Chirality at C99         (Sohnke SpGr)          S Verify
PLAT791_ALERT_4_G Model has Chirality at C101        (Sohnke SpGr)          S Verify
PLAT791_ALERT_4_G Model has Chirality at C102        (Sohnke SpGr)          R Verify
PLAT791_ALERT_4_G Model has Chirality at C103        (Sohnke SpGr)          S Verify
PLAT791_ALERT_4_G Model has Chirality at C104        (Sohnke SpGr)          S Verify
PLAT791_ALERT_4_G Model has Chirality at C105        (Sohnke SpGr)          R Verify
PLAT791_ALERT_4_G Model has Chirality at C108        (Sohnke SpGr)          R Verify
PLAT791_ALERT_4_G Model has Chirality at C109        (Sohnke SpGr)          S Verify
PLAT791_ALERT_4_G Model has Chirality at C110        (Sohnke SpGr)          S Verify
PLAT791_ALERT_4_G Model has Chirality at C111        (Sohnke SpGr)          R Verify
PLAT791_ALERT_4_G Model has Chirality at C112        (Sohnke SpGr)          S Verify
PLAT791_ALERT_4_G Model has Chirality at C115        (Sohnke SpGr)          S Verify
PLAT791_ALERT_4_G Model has Chirality at C116        (Sohnke SpGr)          R Verify
PLAT791_ALERT_4_G Model has Chirality at C117        (Sohnke SpGr)          S Verify
PLAT791_ALERT_4_G Model has Chirality at C118        (Sohnke SpGr)          S Verify
PLAT791_ALERT_4_G Model has Chirality at C119        (Sohnke SpGr)          R Verify
PLAT791_ALERT_4_G Model has Chirality at C122        (Sohnke SpGr)          R Verify
PLAT791_ALERT_4_G Model has Chirality at C123        (Sohnke SpGr)          S Verify
PLAT791_ALERT_4_G Model has Chirality at C124        (Sohnke SpGr)          S Verify
PLAT791_ALERT_4_G Model has Chirality at C125        (Sohnke SpGr)          R Verify
PLAT791_ALERT_4_G Model has Chirality at C126        (Sohnke SpGr)          S Verify
PLAT791_ALERT_4_G Model has Chirality at C149        (Sohnke SpGr)          R Verify
PLAT860_ALERT_3_G Number of Least-Squares Restraints ............         41 Note
PLAT883_ALERT_1_G No Info/Value for _atom_sites_solution_primary .     Please Do !
```

```
   0 ALERT level A = Most likely a serious problem - resolve or explain
  52 ALERT level B = A potentially serious problem, consider carefully
  34 ALERT level C = Check. Ensure it is not caused by an omission or oversight
 109 ALERT level G = General information/check it is not something unexpected

  10 ALERT type 1 CIF construction/syntax error, inconsistent or missing data
  72 ALERT type 2 Indicator that the structure model may be wrong or deficient
   9 ALERT type 3 Indicator that the structure quality may be low
 103 ALERT type 4 Improvement, methodology, query or suggestion
   1 ALERT type 5 Informative message, check
```

It is advisable to attempt to resolve as many as possible of the alerts in all categories. Often the minor alerts point to easily fixed oversights, errors and omissions in your CIF or refinement strategy, so attention to these fine details can be worthwhile. In order to resolve some of the more serious problems it may be necessary to carry out additional measurements or structure refinements. However, the purpose of your study may justify the reported deviations and the more serious of these should normally be commented upon in the discussion or experimental section of a paper or in the "special_details" fields of the CIF. checkCIF was carefully designed to identify outliers and unusual parameters, but every test has its limitations and alerts that are not important in a particular case may appear. Conversely, the absence of alerts does not guarantee there are no aspects of the results needing attention. It is up to the individual to critically assess their own results and, if necessary, seek expert advice.

Publication of your CIF in IUCr journals

A basic structural check has been run on your CIF. These basic checks will be run on all CIFs submitted for publication in IUCr journals (*Acta Crystallographica*, *Journal of Applied Crystallography*, *Journal of Synchrotron Radiation*); however, if you intend to submit to *Acta Crystallographica Section C* or *E* or *IUCrData*, you should make sure that full publication checks are run on the final version of your CIF prior to submission.

Publication of your CIF in other journals

Please refer to the *Notes for Authors* of the relevant journal for any special instructions relating to CIF submission.

PLATON version of 12/09/2022; check.def file version of 09/08/2022

Datablock gold6-phase2_auto - ellipsoid plot

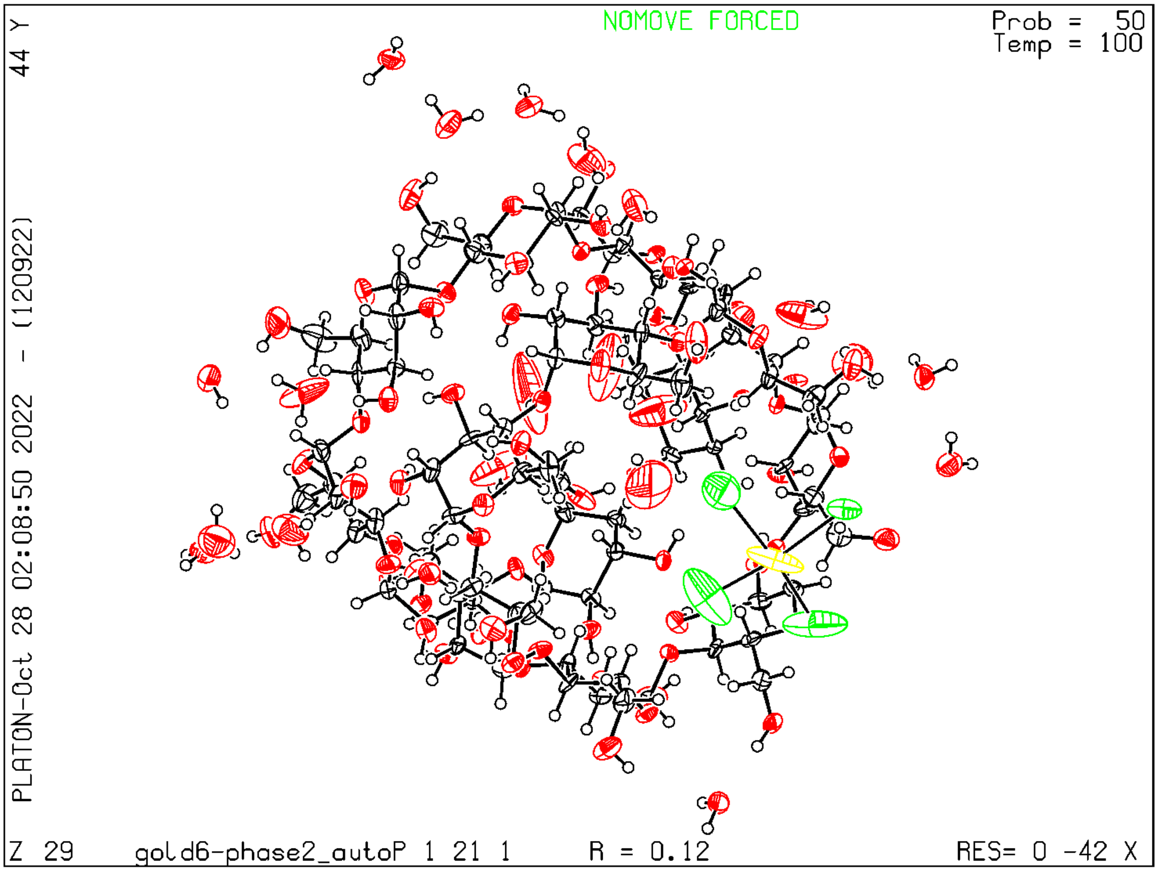

Supplement: Supplementary file 7 — Supplementary Data 4 Checkcif File for 0.5(HAuBr4)⊂2β-CD Cocrystal (Cocrystal B) [file 41467_2023_36591_MOESM7_ESM.pdf]
